# Multilevel social structure and diet shape the gut microbiota of the gelada monkey, the only grazing primate

**DOI:** 10.1186/s40168-018-0468-6

**Published:** 2018-05-05

**Authors:** Pål Trosvik, Eric J. de Muinck, Eli K. Rueness, Peter J. Fashing, Evan C. Beierschmitt, Kadie R. Callingham, Jacob B. Kraus, Thomas H. Trew, Amera Moges, Addisu Mekonnen, Vivek V. Venkataraman, Nga Nguyen

**Affiliations:** 1 Department of Biosciences, Centre for Ecological and Evolutionary Synthesis (CEES), University of Oslo, P.O. Box 1066, Oslo, Norway; 2 Department of Anthropology and Environmental Studies Program, California State University Fullerton, Fullerton, CA USA; 3 Department of Anthropology, University of California, Santa Barbara, Santa Barbara, CA USA; 4 Bwindi Gorilla Project, Bwindi Impenetrable National Park, Kanungu, Uganda; 5 Department of Anthropology, University of Wisconsin-Madison, Madison, WI USA; 6 Cleve Lodge, Minster Road, Ramsgate, Kent, CT12 4BA England; 7 Department of Biology, Bahir Dar University, Bahir Dar, Ethiopia; 8 Department of Zoological Sciences, Addis Ababa University, Addis Ababa, Ethiopia; 9 Department of Human Evolutionary Biology, Harvard University, Cambridge, MA USA

**Keywords:** Cellulolytic bacteria, Ecological specialist, Ethiopian highlands, GI microbiota, Graminivory, Multilevel society, Primates, Rumen

## Abstract

**Background:**

The gelada monkey (*Theropithecus gelada*), endemic to the Ethiopian highlands, is the only graminivorous primate, i.e., it feeds mainly on grasses and sedges. In spite of known dental, manual, and locomotor adaptations, the intestinal anatomy of geladas is similar to that of other primates. We currently lack a clear understanding of the adaptations in digestive physiology necessary for this species to subsist on a graminoid-based diet, but digestion in other graminivores, such as ruminants, relies heavily on the microbial community residing in the gastrointestinal (GI) system. Furthermore, geladas form complex, multilevel societies, making them a suitable system for investigating links between sociality and the GI microbiota.

**Results:**

Here, we explore the gastrointestinal microbiota of gelada monkeys inhabiting an intact ecosystem and document how factors like multilevel social structure and seasonal changes in diet shape the GI microbiota. We compare the gelada GI microbiota to those of other primate species, reporting a gradient from geladas to herbivorous specialist monkeys to dietary generalist monkeys and lastly humans, the ultimate ecological generalists. We also compare the microbiotas of the gelada GI tract and the sheep rumen, finding that geladas are highly enriched for cellulolytic bacteria associated with ruminant digestion, relative to other primates.

**Conclusions:**

This study represents the first analysis of the gelada GI microbiota, providing insights into the adaptations underlying graminivory in a primate. Our results also highlight the role of social organization in structuring the GI microbiota within a society of wild animals.

**Electronic supplementary material:**

The online version of this article (10.1186/s40168-018-0468-6) contains supplementary material, which is available to authorized users.

## Background

All animals are intimately associated with complex consortia of microbes inhabiting accessible body surfaces, and these microbial communities are instrumental to animal physiology and function [1]. The most densely populated part of the mammalian anatomy is the gastrointestinal (GI) tract, where the microbial cells are thought to outnumber host cells [2]. The GI tract is presumed sterile at birth, upon which colonization through exposure commences rapidly to form the GI microbiota. In addition to general exposure, factors such as diet and phylogeny have been found to be important determinants of GI microbiota composition, with GI bacterial communities highly co-evolved to specific lifestyles [3, 4]. The GI microbiota has been found to have plastic responses to changes in diet in humans [5], and in wild primate populations in response to habitat and seasonal variation [6–8]. A recent study even reported humanization of the primate GI microbiota as a result of captivity [9].

Animals are reliant on symbiotic bacteria for breaking down recalcitrant carbohydrates [10]. While ubiquitous, cellulose, the major structural component of plants, cannot be digested by vertebrates without the aid of protozoans or bacterial symbionts. Microbial digestion of cellulose can occur either in the vertebrate foregut (forestomach) or in the mid- or hind-gut (caecum or colon) [11]. Foregut fermentation has evolved only a few times in mammals (e.g., in the ancestors of the ruminants and colobine monkeys), while hindgut fermenting mammals form a comparatively diverse group that includes odd-toed ungulates, rodents, and rabbits, as well as several primate species. The ways in which the microbial systems that aid in the digestive process differ between fore- and hindgut fermenters have not been thoroughly investigated, although studies have found that the two groups tend to cluster separately in terms of their GI microbiomes [3, 4]. Furthermore, comparative meta-studies of these processes are hindered by a high degree of variation in the protocols used for describing complex microbial communities, which can make direct comparison between studies problematic [12].

**Fig. 1:**
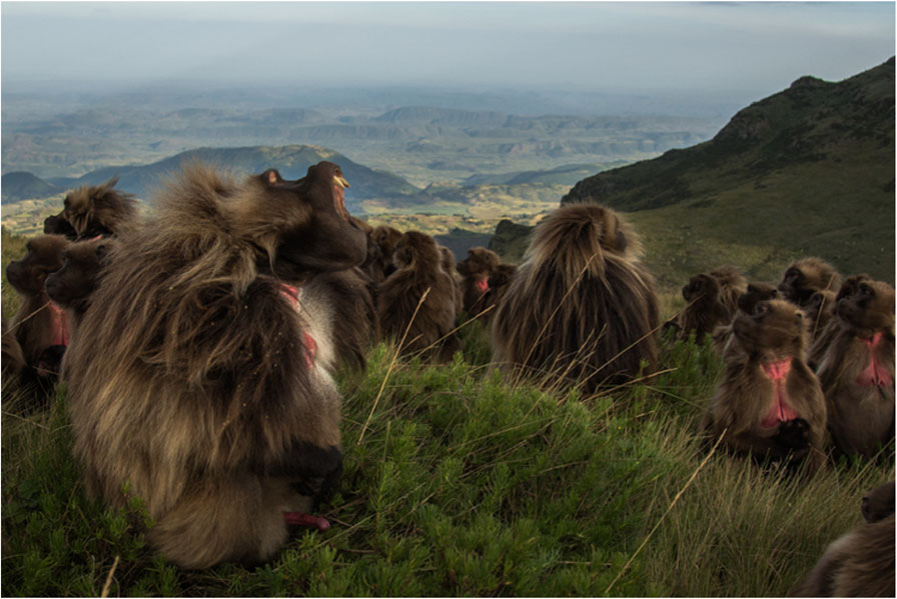
The Steelers band of geladas at Guassa. The great Rift Valley can be seen below in the distance. Photo courtesy of Jeffery T. Kerby

Among extant primates, only one species is an ecological specialist on graminoids (i.e., grasses and sedges): the gelada monkey (*Theropithecus gelada*) [13]. The gelada is the sole remaining species of a once widespread genus of grazing primates [14]. Today, geladas are endemic to the alpine grasslands of the Ethiopian Highlands where they are threatened by climate change and human encroachment [15]. Geladas have several morphological adaptations that help them subsist on this highly specialized diet, including reduced incisors and enlarged molars, as well as elongated, robust thumbs and reduced second fingers that form an effective pincer-like apparatus for harvesting graminoids [16, 17]. These peculiar adaptive traits of extant geladas (*T. gelada*) are also found in extinct *Theropithecus* from as early as 3.7 Ma, suggesting a long-standing reliance on herbaceous plants (graminoids and forbs) in this primate genus [18]. Moreover, geladas employ a characteristic shuffling gait that allows them to move around in a sitting position while harvesting food [19]. The lack of specialized anatomical features in the gelada GI tract suggests that the GI microbiota plays a pivotal role in digesting a high-fiber diet of recalcitrant carbohydrates, possibly through hindgut fermentation [16]. However, the way in which this is achieved, and to what extent the gelada GI microbiome resembles those of other primate species, including humans, as well as other non-primate graminivores, is not known. In fact, an in vitro study using gelada feces to inoculate grass for fermentation found the process to be unexpectedly inefficient [20]. Compared to other highland sites in Ethiopia where geladas occur, such as Simien Mountains National Park, our study site, the Guassa Plateau, represents a relatively intact ecosystem with little disturbance from humans and livestock [13]. This means that the Guassa geladas (Fig. 1) still adhere to what is thought to be their traditional diet, making Guassa an ideal location for studying the GI microbiome of a graminivorous primate in a natural ecological setting.

Geladas live in multilevel societies, which at the lowest tier consist of reproductive units of one or several closely related females with their young and one or a few males. Multiple reproductive units form a larger structure known as a band, which shares a common home range and can include hundreds of animals [21]. Host social behavior has been found to contribute to variation in the composition of the GI microbiota in humans and non-human primates [22–25]. Although most gelada social interactions occur within the reproductive units, a band of geladas travels, eats, and sleeps together, thus making them a suitable model system for investigating the effects of social interactions on the microbiome within the larger context of an animal society.

Here, we use deep 16S rRNA gene amplicon sequencing to analyze 316 fecal samples from 39 female geladas of reproductive age, belonging to eight distinct reproductive units from a single band. The band is one of several that reside on the Guassa Plateau, where the monkeys have been studied intensively for the past decade [26]. Much is therefore known about key life history variables such as group membership, age, health, and dietary habits across changing seasons, and we investigate how these factors work to structure the gelada GI microbiota. To shed light on the adaptations underlying primate graminivory, we compare the gelada GI microbiota to that of humans as well as to three other Ethiopian monkey species, the bamboo-feeding specialist Bale monkey (*Chlorocebus djamdjamensis*) [27], and two dietary generalist species, the vervet (*Chlorocebus pygerythrus*) and the grivet (*Chlorocebus aethiops*) [28]. We further compare the gelada monkey GI microbiota to the rumen microbiota of domestic sheep (*Ovis aries*), a highly specialized graminivorous foregut fermenter. Ours is the first study to describe the GI microbiota of a graminivorous primate, and our findings shed new light on the digestive adaptations underlying this unique dietary specialization. Our study also contributes to a growing body of literature on the links between social structure and the GI microbiota.

## Results

### Intrinsic structuring factors of the gelada GI microbiota

In all 316 gelada samples combined, we identified a total of 1624 different operational taxonomic units (OTUs) on the 97% sequence identity level. In general, low level taxonomic classification was poor with only 11% of OTUs classified to the genus level with a probability of 0.9 or higher. 48 OTUs (3.0% of total) were found in all 316 gelada samples, while 284 OTUs (12.4%) were common to at least 90% of samples, suggesting a limited core microbiome. Half of the 48 OTUs making up the core set were classified as *Bacteroidetes*, with 13 of these assigned to the class *Sphingobacteriia*. In addition, three of the core OTUs were classified as phylum *Verrucomicrobia class Subdivision 5*. A full list of the gelada core microbiota can be found in Additional file 1: Table S1. We also employed an alternative to traditional OTU clustering, the DADA2 algorithm that can resolve amplicon reads to the single nucleotide difference level [29], to evaluate whether finer scale resolution of the sequence data could help explain more of the variation between social groups.

We used three complementary dissimilarity metrics to assess the effects of several structuring factors on the gelada GI microbiota. Bray-Curtis distances are based on taxonomic (OTU assignment) and abundance information. The two variants of the UniFrac metric incorporate phylogenetic distances between OTUs; weighted UniFrac distances also incorporate abundance information, while unweighted UniFrac distances only consider phylogenetic similarity. The strongest structuring factor of the gelada microbiome was variation between individuals (*n* = 39 individuals, *p* < 0.001, PERMANOVA with Bray-Curtis distances). Individual identity explained 20.4% of the between-sample variation in geladas, indicating extensive sharing of the GI microbial community within the band. Individuals belonging to the same reproductive unit had significantly more similar GI microbiotas relative to individuals from different units (*p* < 0.001), with this factor explaining 5.8% of between-sample variation. Age and reproductive status both exerted marginal, though significant, effects on the gelada microbiota (Table 1), while parasitic tapeworm (*Taenia serialis*) disease status had no measurable effect. Using unweighted UniFrac distances as an alternative dissimilarity measure yielded similar results (Additional file 1: Table S2), though the age effect disappeared and there was a marginally significant effect of tapeworm infection when we used weighted UniFrac distances (Additional file 1: Table S3). Thus, the strongest intrinsic structuring factor of the gelada GI microbiota appears related to the social group, while the effects of age and tapeworm infection were marginal and inconsistent.

**Table 1:** PERMANOVA test results for effects on the GI microbiota using 1000 permutations and Bray-Curtis distances. The R-squared values indicate the amount of between-sample variation explained by each variable

Factor	*R* ^2^	*P* value	Groupings
Individual	0.204	0.001	Monkey #1–39 (*n* = 1–12; mean = 8)
Reproductive unit	0.058	0.001	Unit #1–8 (*n* = 8–84; mean = 40)
Season	0.035	0.001	Dry (*n* = 142), wet (*n* = 174)
Age	0.008	0.001	Prime (*n* = 246), old (*n* = 70)
Fecundity status	0.023	0.041	T1 (*n* = 27), T2 (*n* = 13), T3 (*n* = 32), PPA (*n* = 178), cycling (*n* = 66)
Coenurosis		n.s.	Swelling (*n* = 235), no swelling (*n* = 81)

PERMANOVA using the OTU table based on DADA2 sequence variants and Bray-Curtis distances found that reproductive unit explained 5.7% of between sample variation (*p* < 0.001). This result is similar to the one we got using 97% sequence identity OTU clustering (5.8% explained variation), suggesting that strain level differences in GI microbiota composition is not the driving force behind the observed social structure effect. UniFrac distances also gave very similar results (*R*^2^ = 0.052 and 0.055, for weighted and unweighted UniFrac, respectively; *p* < 0.001 for both tests).

### Seasonal effects

**Fig. 2:**
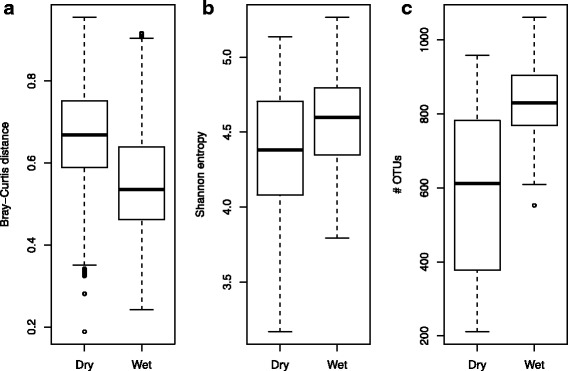
Differences in between-sample Bray-Curtis distances (**a**), Shannon entropy (**b**), and OTU richness (**c**) between gelada samples collected during the dry (*n* = 142) or the wet (*n* = 174) season. In all three cases, differences were highly significant (*p* <  0.001 for all comparisons, unpaired *t* tests). Each box represents the interquartile range, with the horizontal lines representing the medians and the whiskers representing 1.5 times the interquartile range. Points outside the whiskers represent outliers

Overall, the most abundant phylum in the gelada GI microbiota was *Bacteroidetes* (44.5%) (Additional file 1: Figure S1), followed by *Firmicutes* (34.6%) and *Spirochaetes* (4.5%). Samples collected during the dry and wet seasons differed significantly from one another (< 0.001, PERMANOVA with Bray-Curtis distances), with season of sampling explaining 3.5% of between-sample variation. The seasonal effect was also evident when using UniFrac distances (Additional file 1: Tables S2 and S3). We found that inter-individual variation in the GI microbiome was much higher during the dry than the wet season, i.e., between-sample Bray-Curtis distances were greater during the dry relative to the wet season (means of 0.67 vs. 0.56, respectively, *p* < 0.001, unpaired *t* test) (Fig. 2a). This result was maintained when using UniFrac distances (Additional file 1: Figure S2). The mean relative abundance of the phylum *Bacteroidetes* was significantly higher during the wet (47.3%) relative to the dry (42.2%) season (Additional file 1: Figure S1; *p* < 0.001, Wilcoxon rank sum test), while *Firmicutes* and *Spirochaetes* were more abundant during the dry season (36.3 vs. 32.6% and 5.3 vs. 3.6%, respectively; *p* < 0.001). Out of the 168 most prevalent OTUs, 103 had significant differential occurrence across seasons, with 93% of these enriched during the dry season. The affected OTUs were mostly classified as *Bacteroidetes* (44%) or *Firmicutes* (36%), while 9% were classified as phylum *Verrucomicrobia class Subdivision 5*. The full table can be found in supplementary information (Additional file 1: Table S4). Interestingly, of all the variables we investigated, only season had a significant effect on observed diversity, as measured both by Shannon entropy (Fig. 2b) and OTU richness (Fig. 2c), with elevated diversity levels during the wet season (*p* < 0.001 for both comparisons, unpaired *t* test). Indeed, the mean number of observed OTUs rose from 587 during the dry to 837 during the wet season, an increase of 42.6%.

### Comparison with humans and other non-human primates

**Fig. 3:**
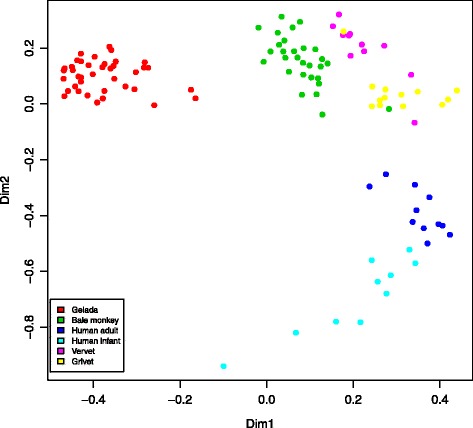
Non-metric multidimensional scaling of all primate samples based on the Bray-Curtis distance matrix. The plot shows the two main dimensions of variation, with plotted characters color coded according to sample type. Clustering according to samples type was highly significant, explaining 43% of between-sample variation (*p* < 0.001, PERMANOVA). The model stress value was 10.7. Gelada (*n* = 39), Bale moneky (*n* = 29), human adult (*n* = 11), human infant (*n* = 10), vervet (*n* = 11), grivet (*n* = 13)

**Fig. 4:**
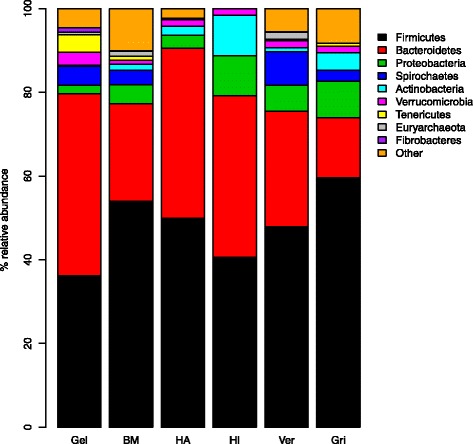
Mean relative abundances of the nine most prevalent phyla in the six categories of primate samples. Gel, gelada (*n* = 39); BM, Bale monkey (*n* = 29); HA, human adult (*n* = 11); HI, human infant (*n* = 10); Ver vervet (*n* = 11); Gri, grivet (*n* = 13). The category “Other” includes OTUs that could not be classified to the phylum level with a probability higher than 0.5

To gain insights into the adaptations of the GI microbiota underlying graminivory in a primate, we compared the gelada GI microbiota, based on one randomly selected fecal sample from each of the 39 individual geladas, with the GI microbiotas of Bale monkeys, vervets and grivets, as well as human adults and infants. In this dataset, we identified 2280 97% sequence identity OTUs. The primate species formed distinct clusters (Fig. 3) with the primary axis of variation distinguishing geladas from other primates and the secondary axis distinguishing humans from monkeys. Species explained 42.9% of the total variation in between-sample Bray-Curtis distances (*p* < 0.001, PERMANOVA) with the percentage increasing to 45.2% when considering human infants as a separate group (which will be the case for all upcoming comparisons). Using UniFrac as a distance measure yielded very similar results (Additional file 1: Figure S3). On the phylum level (Fig. 4), geladas had less *Firmicutes* and *Proteobacteria*, but more *Bacteroidetes* than the other monkeys (*p* < 0.001 for all comparisons, Wilcoxon rank sum test). While the mean relative abundance of *Firmicutes* was less than for human adults (*p* < 0.001), it was comparable to human infants, and the abundance of *Proteobacteria* was marginally less in geladas compared with human adults and infants (*p* = 0.054 and 0.057, respectively). The mean relative abundance of *Bacteroidetes* was not significantly different between geladas and human adults or infants. *Actinobacteria*, a relatively abundant phylum in all other primate groups (Fig. 4), was comparatively rare (0.24% mean relative abundance) in geladas (*p* < 0.001 for all comparisons). Geladas had highly elevated levels of *Fibrobacteres* (1.2%) relative to all other groups (*p* < 0.001 for all comparisons), and this pattern was also observed for *Verrucomicrobia* (*p* < 0.025 for all comparisons) and *Tenericutes* (*p* < 0.001 for all comparisons). *Spirochaetes* were relatively abundant in all monkey species but practically absent in both human adults and infants. Two hundred and nineteen OTUs (of a total of 2280) were exclusive to gelada monkeys. The majority of these were *Firmicutes*, but 15 were classified to phylum *Verrucomicrobia class Subdivision 5*, while 14 were phylum *Bacteroidetes* class *Sphingobacteriia*. The full list can be found in Supplementary information (Additional file 1: Table S5).

EdgeR exact tests found that out of the 458 most prevalent OTUs in the combined primate dataset, 146 were significantly enriched in gelada monkeys, while 211 were significantly underrepresented. Among the top ten OTUs enriched in geladas, including the top two, three OTUs were classified to the phylum *Verrucomicrobia class Subdivision 5*. Four of the top ten were classified as *Bacteroidetes*, putatively to the family *Sphingobacteriaceae* (albeit at low assignment probabilities: 0.05–0.19), and out of these four, three OTUs were found at mean relative abundances of > 1.5%. A full overview of the enrichment analysis, including taxonomic assignment probabilities, can be found in Supplementary information (Additional file 1: Table S6).

We also estimated the combined core microbiota of all three *Chlorocebus* species for comparison with the gelada core set. This analysis identified 86 OTUs that were present in all of the *Chlorocebus* monkey individuals (Additional file 1: Table S7). Fifty percent of the gelada core OTUs was classified as *Bacteroidetes*, with 27% further classified to the class *Sphingobacteriia* and family *Sphingobacteriaceae*. In contrast, *Bacteroidetes* constituted only 13% of the *Chlorocebus* core with only 2% classified as *Sphingobacteriaceae*. Not a single OTU in the C*hlorocebus* core was classified to phylum *Verrucomicrobia*, and the *Chlorocebus* core microbiota was generally dominated by *Firmicutes* (77%), which only constituted 33% of the gelada core set.

In terms of overall mean relative abundance, OTUs classified as *Sphingobacteriaceae* constituted 27.7% of the gelada microbiota compared to 4.5% and 3.2% in vervets and Bale monkeys, respectively, 0.6% in grivets, 0.5% in human adults, and < 0.1% in infants. It should be noted that these OTUs were classified with low confidence, even at the class level (mean assignment probability of 0.27). OTUs classified as *Verucomicrobia Subdivision 5* were also highly abundant in geladas at 3.1% mean relative abundance, more than twice that of any other monkey species. These OTUs were found at 0.3% mean relative abundance in human adults, while not a single one was found in the infants.

**Fig. 5:**
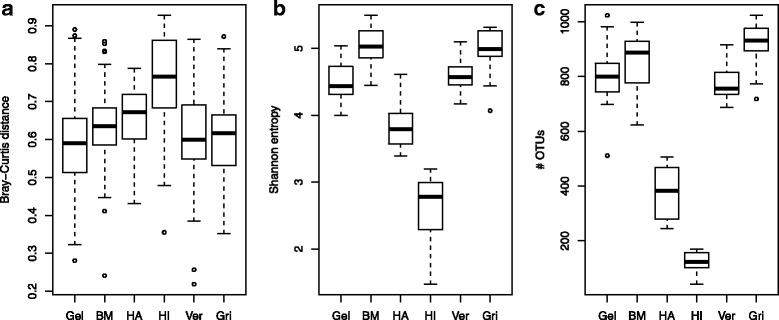
Differences in within-group Bray-Curtis distances (**a**), Shannon entropy (**b**), and OTU richness (**c**) among primate samples. **a** Inter-individual variation in geladas was significantly lower than in Bale monkeys and human adults and infants (*p* < 0.001, unpaired *t* test) and marginally lower than in vervets and grivets (*p* = 0.087 and 0.072, respectively). Infants had significantly higher variation than all other groups (*p* < 0.001 for all comparisons). **b** Shannon entropy was significantly lower in geladas than in Bale monkeys and grivets, and higher than in human adults and infants (*p* < 0.001 for all comparisons). **c** OTU richness was significantly lower in geladas than in Bale monkeys and grivets (*p* < 0.05 for both comparisons), and higher than in human adults and infants (*p* < 0.001 for both comparisons). Each box represents the interquartile range, with the horizontal lines representing the medians and the whiskers representing 1.5 times the interquartile range. Points outside the whiskers represent outliers. Gel, gelada (*n* = 39); BM, Bale monkey (*n* = 29); HA, human adult (*n* = 11); HI, human infant (*n* = 10); Ver, vervet (*n* = 11); Gri, grivet (*n* = 13)

Geladas had the lowest amount of inter-individual GI microbiota variation (Fig. 5a) with a mean Bray-Curtis distance of 0.59 which was significantly lower than in Bale monkeys and human adults and infants (*p* < 0.001, unpaired *t* tests) and marginally lower than in vervets and grivets (*p* = 0.087 and 0.072, respectively). Within-group Bray-Curtis distances were significantly elevated in both human groups relative to all four monkey species (*p* < 0.05), and inter-individual variation was particularly high in the infants (mean of 0.76). Similar patterns were also observed when using UniFrac distances as alternative dissimilarity metrics (Additional file 1: Figure S4). Diversity in the GI microbiomes of the monkey species was comparable both as measured by Shannon entropy (Fig. 5b) and OTU richness (Fig. 5c), but much reduced in humans relative to the monkeys. In particular, the number of OTUs observed in human adults was about half of what was observed in monkeys, and in infants the number was reduced nearly sevenfold.

### Comparison with the sheep rumen microbiota

**Fig. 6:**
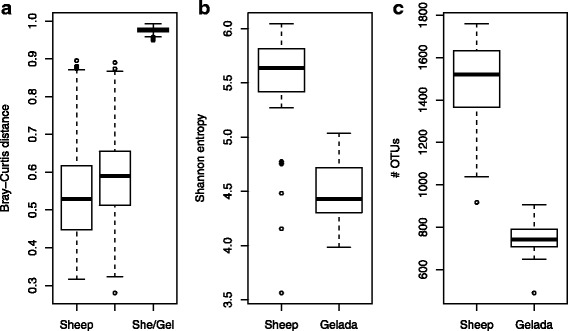
Differences in within-group Bray-Curtis distances (**a**), Shannon entropy (**b**), and OTU richness (**c**) between geladas (*n* = 39) and sheep (*n* = 29). Each box represents the interquartile range, with the horizontal lines representing the medians and the whiskers representing 1.5 times the interquartile range. Points outside the whiskers represent outliers. In **a**, the between-sample distances were significantly reduced in the sheep relative to the geladas (*p* < 0.001, unpaired *t* test), while the mean distance between geladas and sheep (She/Gel) was close to one. Diversity was significantly elevated in sheep relative to geladas (*p* <  0.001 for both comparisons, unpaired *t* tests)

**Fig. 7:**
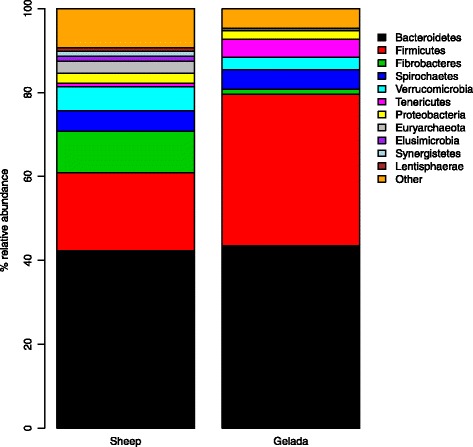
Mean relative abundance of the 11 most prevalent phyla in geladas (*n* = 39) and sheep (*n* = 29). The category “Other” includes OTUs that could not be classified to the phylum level with a probability higher than 0.5

We also compared the gelada GI microbiota with that of a non-primate domestic graminivore. This analysis focused on the 39 gelada samples from the previous analysis and 29 sheep rumen samples from domestic sheep housed at three different farms in central Norway. Comparing feces and rumen content is maybe not ideal, but the GI microbiota of the wild geladas at Guassa can only be studied through non-invasive sampling, while the rumen is the site of cellulose digestion in sheep. OTU clustering of this dataset resulted in the identification of 2979 OTUs on the 97% sequence identity level. Inter-individual variation was significantly lower in sheep than in geladas (*p* < 0.001, unpaired *t* test), while the mean Bray-Curtis distance between sheep and geladas was close to 1 (Fig. 6a), indicating little overlap between the GI microbiotas of the two species (Additional file 1: Figure S5). Using UniFrac distances as a dissimilarity metric produced similar results (Additional file 1: Figs. S6 and S7). In fact, only 17 OTUs, i.e., less than 0.6% of the total OTU number, were shared between all gelada and sheep samples, with these OTUs accounting for 4.9% of the total mean relative abundance in the gelada GI microbiota and 3.0% in the sheep rumen. Out of these 17 OTUs, 14 were classified as *Firmicutes*, many with poor classification accuracy. The final three OTUs included a poorly classified *Tenericutes*, a *Prevotella*, and a methanogenic archaeon. The full list can be found in Additional file 1: Table S8. With relaxed criteria, the number of shared OTUs increased (Additional file 1: Figure S8), with 162 OTUs found in at least 50% of samples from both geladas and sheep (Additional file 1: Table S9), with these OTUs collectively accounting for 15.9 and 7.2% of the total mean relative abundance in geladas and sheep, respectively. The majority (72%) of theses OTUs were *Firmicutes*, mostly classified to the families *Ruminococcaceae* (41), *Lachnospiraceae* (33), and *Clostridiaceae* (17). Only nine OTUs were classified as *Bacteroidetes* (two classified as family *Sphingobacteriaceae*), while nine were *Proteobacteria*, three were *Verrucomicrobia Subdivision 5*, and three were *Euryarchaeota*. Shannon entropy (Fig. 6b) and OTU richness (Fig. 6c) were highly elevated in sheep relative to geladas (*p* < 0.001 for both comparisons, unpaired *t* test), with the sheep rumen microbiota harboring, on average, nearly twice as many OTUs as the gelada GI microbiota (1475 vs. 746 OTUs). On the phylum level, there were substantial differences between the two species (Fig. 7). The mean relative abundance of *Firmicutes* was much higher in geladas than in sheep (36.1 vs. 18.6%; *p* < 0.001, Wilcoxon rank sum test), and this was also the case for *Tenericutes* (4.2 vs 0.9%; *p* < 0.01). Several phyla were found at significantly higher mean relative abundances in sheep relative to geladas, including *Verrucomicrobia* and *Euryarchaeota* (*p* < 0.001 for both comparisons). In particular, *Fibrobacteres* and *Lentisphaerae* were much more common in sheep (mean of 9.9 vs. 1.2%, and 0.8 vs. 0.1%, respectively; *p* < 0.001 for both comparisons), while *Elusimicrobia* occurred at a mean level 25 times that observed in geladas. The relatively rare phyla *Synergistetes*, *Chloroflexi*, and candidate division *SR1* were all recovered from sheep at mean relative abundances between 0.2 and 1.1%, and not a single read classified to any of these groups was found in the gelada samples. Among the 28 OTUs classified as *Fibrobacteres*, 8 were found exclusively in geladas while the remaining 20 were found only in sheep. The gelada OTUs formed a monophyletic group along with two of the sheep OTUs, the rest of which showed a higher degree of phylogenetic diversity (Additional file 1: Figure S9).

Overall, OTUs putatively classified to the family *Sphingobacteriaceae* were highly abundant in both geladas and sheep, at 27.7 and 19.7% mean relative abundance, respectively. On the other hand, diversity within this family was much higher in sheep with 180 OTUs versus 43 in geladas. Again, we would like to point out that these OTUs were classified with low confidence, even on the class level (*Sphingobacteriia*). OTUs classified as *Verrucomicrobia Subdivision 5* were also highly abundant in sheep at 5.0% mean relative abundance (3.1% in geladas).

We also compared sheep to the other primate in our study. Using OTU level Bray-Curtis distance as a dissimilarity measure, we did not find compelling evidence that the gelada GI microbiota resembled the sheep rumen microbiota more than those of the other monkey species. However, when using phylum level Bray-Curtis distances, the gelada GI microbiota was significantly more similar to sheep than to the microbiotas of any of the other primates in our study (Additional file 1: Figs. S10 and S11; *p* < 0.001 for all comparisons). When using UniFrac (OTU level) distances, geladas were found to be significantly more similar to sheep than to any other primate group (Additional file 1: Figure S12; *p* < 0.001 for all comparisons, unpaired *t* test). These results indicate that although geladas and sheep share few specific OTUs, the gelada GI microbiota is more phylogenetically more similar to sheep than any other primate species in this study.

### Functional profiling of microbiotas

Lastly, we estimated the bacterial and archaeal genes present in the metagenomes of all the animals in our study using the PICRUSt algorithm [30]. The accuracy of PICRUSt’s predicted metagenomes can, however, vary depending on the extent to which sequenced genomes for the OTUs in our samples are available in the reference genome database. The average Nearest Sequenced Taxon Index (NSTI) provides a measure of how well a microbiota can be matched to the reference database, with high scores indicating that few related references are available. NSTI values were highest in geladas (mean 0.294 ± 0.034 s.d.), and as expected, they were much lower for humans than for any of the monkey species (Additional file 1: Figure S13a). The difficulty of finding references for OTUs associated with the gelada GI microbiota in the current genome database was further reflected in the much lower mean number of assigned KEGG orthologs (KOs) in this species relative to the other animals we sampled (*p* < 0.001 for all comparisons, unpaired *t* test) (Additional file 1: Figure S13b). The number of KOs was also much lower in geladas than in sheep (*p* < 0.001), although the extremely high diversity of the rumen microbiota would probably contribute to a higher number of assigned KOs relative to geladas. Given these limitations, the results of our PICRUSt analysis (below) should be interpreted with caution. Furthermore, in the following analyses, counts of assigned functional classes are normalized to relative abundances to account for differential assignment efficiency.

**Fig. 8:**
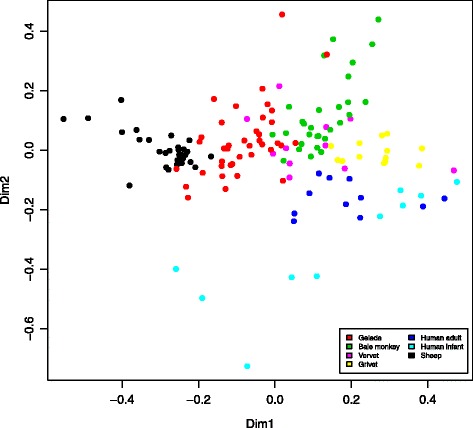
Non-metric multidimensional scaling of primates and sheep based on the Bray-Curtis distance matrix computed from relative abundance table of KEGG orthologs. The plot shows the two main dimensions of variation, with plotted characters color coded according to sample type. Clustering according to samples type was highly significant, explaining 56% of between-sample variation (*p* < 0.001, PERMANOVA). The model stress value was 10.5. Sheep (*n* = 29), gelada (*n* = 39), Bale monkey (*n* = 29), human adult (*n* = 11), human infant (*n* = 10), vervet (*n* = 11), and grivet (*n* = 13)

The functional profiles showed strong clustering by group (*R*^2^ = 0.54, *p* < 0.001, PERMANOVA with Bray-Curtis distances), with geladas being more similar to sheep than to any other primate group (Fig. 8, Additional file 1: Figure S14). In a number of functional categories, we saw differentiation in metabolic potential from ecological specialists (sheep, geladas, and Bale monkeys) to generalist monkeys and humans. For example, the proportion of metabolic capacity dedicated to the metabolism of several groups of amino acids followed a gradient from high in specialists to low in generalists (Additional file 1: Figure S15). Interestingly, we observed the same trend for degradation of limonene and pinene (Additional file 1: Figure S16). These substances belong to a class of hydrocarbons known as terpenes, the largest class of plant secondary metabolites [31]. We also looked for specific enrichment of the three main cellulolytic enzyme categories: β-1,4-endoglucanases (KEGG orthology K01179), β-1,4-exoglucanases (K01225), and β-glucosidases (K05350) [32]. β-1,4-endoglucanases were significantly enriched in sheep relative to primates (*p* < 0.001 for all comparisons, unpaired *t* test), and in geladas relative to humans and grivets (*p* < 0.001), but not to Bale monkeys and vervets (Additional file 1: Figure S17a). β-1,4-exoglucanases were enriched in geladas relative to sheep and humans (*p* < 0.001), but not relative to the other monkey species (Additional file 1: Figure S17b). β-glucosidases were enriched in geladas relative to sheep and all primate groups (*p* < 0.02) except Bale monkeys (Additional file 1: Figure S17c). We further compared the putative taxa that contributed cellulolytic functionality in geladas and sheep. At the family level, the main taxa contributing cellulolytic functions were *Ruminococcaceae*, *Clostridiaceae*, and *Lachnospiraceae* (Additional file 1: Table S10). Notably, producers of β-1,4-endoglucanases were dominated by those three families, but in sheep, 10.3% were contributed by *Prevotellaceae*. In addition to the three main families, producers of β-glucosidases also included considerable numbers of OTUs classified as family *Erysipelotrichaceae*.

## Discussion

Here, we present the first study of the microbiota of gelada monkeys, the only extant primate graminivores. We report consistent individual microbiota profiles among adult females belonging to eight reproductive units, with relatively strong effects of social group membership and seasonal feeding habits. We further describe a gradient in microbiota composition from herbivorous to omnivorous primates, and we identify bacterial groups associated with ruminant digestion that were highly enriched in geladas relative to other primates. We also identify putative metabolic pathways distinguishing grass feeding specialists from ecological generalists. From human studies, GI microbiomes are known to be highly individual [22], although temporal variation within an individual can be substantial [33]. A recent study compared the bacterial content in sequentially collected fecal samples from five healthy adult humans, finding that almost 75% of inter-sample variation in Bray-Curtis distances could be explained by sample origin [34]. In contrast, we found that individuality only explained 20.4% of between-sample variation within geladas at Guassa. Likely explanations for this observation are a highly uniform diet [13] and extensive sharing of GI microbes as a result of co-habitation by the many reproductive units within a band [21, 26]. Investigations into other similar systems like hamadryas baboons [35], snub-nosed monkeys [36], and elephants [37] will be needed to establish the degree to which individual microbiome signatures are blurred by living in a large multilevel society within which behaviors are highly synchronized.

We found a significant effect of social group membership on the GI microbiota in geladas. This finding supports the view that convergence in host GI microbial communities among the members of a social group is due in part to shared environment (including diet and social contact). Similar effects of social group membership on the GI microbiota have now also been documented in several well-studied primate species, including yellow baboons [24], chimpanzees [25, 38] and sifaka lemurs [39]. Tung et al. [24] compared two neighboring groups of baboons, finding that group membership explained 18.6% of the total variation in Bray-Curtis distances between samples. They further found that animals with a closer grooming partnership had more similar GI microbiotas and that this result was independent of kinship. Degnan et al. [38] similarly compared chimpanzees belonging to two different social groups with adjacent territories, finding distinct GI microbiome signatures between groups and no discernible effect of genetic relatedness. Moeller et al. [25] found that social interactions affected the diversity of the GI microbiota in a group of chimpanzees, with more diverse GI microbiotas during periods of increased social interaction. Springer et al. [39] studied two neighboring groups of Verreaux’s sifakas, finding that group provenance explained 11.4–15.4% of GI microbiota variation, depending on the distance measure used. In our study, social group membership, although highly significant, accounted for only 5.8% of inter-sample variability. This may be explained by the fact that gelada reproductive units are not analogous to the discrete social groups formed by other primates. While groups of baboons, chimps, and sifakas have their own territories, all eight gelada reproductive units in this study are part of a single band, traveling together and sharing a common food supply [13, 26]. Thus, it is to be expected that the reproductive unit signature in geladas would be weaker than in other social primates. Although we are unable, at present, to evaluate the extent to which kinship, shared diet, or social contact contribute to the social unit signature in the gelada GI microbiota, future studies of geladas and other social animals should endeavor to address this particular issue. This would allow us to draw more definitive conclusions about the specific factors driving observed GI microbiota differences between animal social groups.

Our finding of a putative age effect on the composition of the gelada GI microbiota is consistent with previously documented impacts of aging on the GI microbiota in humans [40, 41]. Studies of wild primates have also documented significant, although subtle, differences in GI microbiota composition between juvenile and adult animals [39, 42]. All individuals sampled in this study were of reproductive age, although the age range varied by at least 10 years in some cases, and it should be pointed out that the observed effect was marginal and varied depending on the dissimilarity metric used for comparison. Aging is known to impact physiological traits important to the GI microbiota such as digestive function [43] and immune function [44], although it is not known whether the older monkeys in this study were of an age when these effects may be expected to be significant. A previous study of gelada chewing efficiency found that older individuals exhibited greater dental wear and were expected to avoid tough graminoids [17]. Thus, age effects on the GI microbiota may also reflect differences in dietary patterns.

We also observed a marginal though significant and consistent effect of reproductive status on the gelada GI microbiome. During a normal pregnancy, the human body goes through extensive hormonal, immunological, and metabolic changes [45, 46], and substantial remodeling of the GI microbiome has been documented from the first to the third trimester [47]. In contrast, a recent study did not find any effect of reproductive state on the GI microbiome in sifakas [39]. Our results raise the possibility that host reproductive status may, in some instances, influence the composition of the GI microbiota.

There is some evidence that intestinal parasites can affect the composition of the GI microbiota in wild animals [48], presumably through direct biotic interactions as well as indirectly through effects on the immune system. Compared to their healthy counterparts, geladas at Guassa with visible tapeworm (*Taenia serialis*) infection of the skin suffer from reduced survival and reproductive rates [26]. Tapeworm infection could potentially influence the GI microbiota by modulating immune function, but we did not observe a consistent effect in our study.

Seasonal variation in GI microbiota composition has been documented in humans [49, 50] and other primates [6, 8, 39], as well as in wild mice [51]. This variation can be explained by differential availability of food items associated with variation in ecological conditions. During the dry season, geladas increase consumption of underground storage organs like roots, tubers, and corms, while during the wet season, their diets are more dominated by green plants [13]. The elevated between-sample distances observed during the dry season (Fig. 2a) may thus be attributed to less uniform foraging patterns, perhaps indicative of subtle niche partitioning, that included fewer green plants, more underground food items, and more opportunistic feeding. Elevated GI microbiota diversity during the wet season (Fig. 2b, c) may reflect the need for a more diverse GI microbiota associated with a diet richer in plant fiber [3].

The effects on the GI microbiome of factors like dietary differences, genetic background, and geographical isolation can be difficult to disentangle. There is evidence to suggest that in hominids components of the GI microbiota have cospeciated with their hosts [52], indicating that microbiome-host relationships can be conserved at the species level over evolutionary time. On the other hand, there is ample evidence that the GI microbiota is highly responsive to dietary change [5, 53]. Hale et al. [54] investigated nine species of colobine monkeys held in captivity at five different locations, finding that their GI microbiotas clustered according to diet/location rather than to phylogeny. Further, Clayton et al. [9] found that the GI microbiotas of primates in a zoo diverged from their wild relatives to become more human-like, demonstrating strong environmental forcing of the GI microbial ecosystem. In our study, the four monkey populations all originate from distinct geographical locations in Ethiopia, with the three *Chlorocebus* species forming a phylogenetically close group. The gelada GI microbiota was clearly distinct from those of the other primates, with Bale monkeys being the most similar to geladas (Fig. 3). The Bale monkeys in this study subsist mainly on the shoots and leaves of bamboo (*Arundinaria alpina*), a species in the family Poaceae which also includes grasses and sedges, although Bale monkeys are not graminivores because they are not grazers [27]. Nevertheless, geladas and Bale monkeys may both be considered ecological specialists with Poaceae as a major dietary component, and the Bale monkey GI microbiota was clearly distinct from that of vervets and grivet. Vervets and grivets are ecological generalists with varied diets of fruit, leaves, and seeds [28]. In addition, some populations raid a variety of human crops [55, 56]. Thus, it is perhaps not surprising that these two species showed the closest similarity to human adults. Human adults in western countries generally have varied diets and lifestyles, which may explain the higher between-sample distances within this group (Fig. 5a). Human infants are known to have dynamic GI microbiotas with a high degree of inter-individual variation [57] which is reflected in the extremely high between-sample distances (Fig. 5a) and the subsequent diffuse clustering (Fig. 3) in this group. This phenomenon has been reported in a previous study [22] and may mean that infants are subject to microbial colonization through random exposure, while adults have stronger selection against opportunistic colonization. Ley et al. [3] documented a diversity gradient from herbivores to omnivores to carnivores, with the highest diversity in herbivores. In agreement with these results, humans harbored much less diverse microbiotas relative to monkeys (Fig. 5b, c), with OTU diversity greatly reduced in human infants relative to human adults, also in keeping with previous work [22].

Baboons, terrestrial, sexually-dimorphic monkeys found throughout Africa, are the closest living relatives of geladas [58] yet are ecological generalists feeding on a variety of fruits, seeds, and leaves. Tung et al. [24] used shotgun metagenomic sequencing to characterize the GI microbiome of two groups of yellow baboons (*Papio cynocephalus*) at Amboseli, Kenya. The phylum level composition of the Amboseli baboon GI microbiota differs markedly from that presented here for geladas. Most notably, *Bacteroidetes*, the dominant phylum in geladas (mean relative abundance of 44.5%), were found to, on average, constitute 7.3% of the total microbiota diversity in baboons. Also, while *Firmicutes*, *Spirochaetes*, and *Tenericutes* were found at similar levels in geladas and baboons, levels of *Actinobacteria* and *Proteobacteria* were much higher in baboons than in geladas. In our study, the two latter phyla were also found at lower levels in geladas relative to all the other primate groups, suggesting that this may be a distinguishing trait of the gelada GI microbiota. Finally, unlike in geladas, OTUs classified either as *Fibrobacteres* and *Verrucomicrobia* were not found at appreciable levels in baboons. The absence of *Fibrobacteres* in baboons may be a reflection of dietary differences between these closely related primates, but any comparison between our study and the study by Tung et al. should be interpreted with caution since widely different techniques were used for characterizing the microbiotas.

Colobus monkeys are folivores that rely on foregut fermentation [59], and two studies have described the GI microbiota of three different species of colobus monkeys, in the wild, using high-throughput sequencing [60, 61]. In both of these studies, the GI microbiotas were found to be dominated by *Firmicutes* (65–80% relative abundance), and neither study found appreciable levels of *Fibrobacteres* (< 0.01% relative abundance). Thus, colobus monkeys appear substantially different from the geladas and sheep characterized here. It should be noted, however, that both colobus studies were done using 454 pyrosequencing, targeting a different 16S rRNA gene region (V1-V3) than in our study (V4), and any direct comparison with our study should be regarded with caution.

The site of cellulose digestion in the body likely impacts the structure and composition of the GI microbiota. Indeed, a study of the GI microbiotas of 60 mammalian species found that herbivorous species (which make up 80% of extant mammals) clustered into two distinct groups representing hind-gut fermenters and fore-gut fermenters [3]. The results of our study are broadly consistent with this finding. We found limited overlap between geladas and sheep in terms of OTU content (Fig. 6a), indicating that graminoid digestion in primates and ruminants rely on quite different microbial systems. It should be noted that the comparison made in this study was between gelada fecal samples and rumen content from sheep. This comparison is perhaps not ideal, but we can only access the GI microbiota of the wild Guassa geladas by analyzing fecal samples collected non-invasively. We chose to use sheep rumen content since this is the site of cellulose digestion in this species. Furthermore, the microbes of the rumen serve as an important source of protein for ruminants, and as rumen fluid leaves the rumen, much of the microbial content is digested in a stomach chamber known as the abomasum [62]. Thus, fecal material may provide an especially poor representation of the microbial community responsible for digestive function in ruminants. The geladas and domestic sheep also came from very distinct geographical environments with access to quite different graminoids for food. While factors like these are sure to affect the types of bacteria found in the microbiotas of the sheep and geladas in this study, we believe that the striking differences we observed were more likely a result of their divergent adaptive processes for digesting herbaceous vegetation. The 17 OTUs found in all gelada and sheep samples account for substantial proportions of their respective microbiotas (4.9% for geladas and 3.0% for sheep). Taxonomic classification of these 17 OTUs was generally poor, with only 5 classified to the genus level with an assignment probability of > 0.80. One of these five OTUs had 99% sequence identity to both *Pseduobutyrovibrio ruminis* and *Pseudobutyrovibrio xylanivorans*, bacterial species associated with ruminant digestion [63].

We found that several microbial phyla are highly enriched in geladas relative to the other primates we sampled, which suggests that these bacteria may be critical for the degradation of cellulose and other resistant starches in the graminoids that make up the bulk of the geladas’ diet. It is noteworthy that OTUs classified to the phylum *Fibrobacteres* constituted 1.2% of the mean relative abundance in geladas (Fig. 4). This is 10× the abundance of *Fibrobacteres* observed in Bale monkeys, 20× compared to vervets and grivets, 100× the abundance in adult humans, and nearly 10,000× that in human infants. *Fibrobacteres* is an underexplored phylum that is strongly associated with cellulose degradation in ruminants [64], and they made up nearly 10% of the total sheep rumen microbiota in this study (Fig. 7). Interestingly, of the 28 OTUs classified as *Fibrobacteres* in the gelada/sheep data set, 20 were found in sheep while 8 were found in geladas, without a single OTU shared between the two species (Additional file 1: Figure S4). In general, nearly 150 OTUs were shared between at least half the members of each group, while more than 350 were shared between at least 10%, indicating that particular *Fibrobacteres* phylotypes are highly specialized to their host species.

The members of the family *Sphingobacteriaceae* have been isolated from environments as diverse as Arctic soil [65], wood [66], animal guts [67], and feces [68]. These bacteria are known for degrading recalcitrant plant substances [65, 67, 69], and a recent study of the dynamics of rice straw degradation in the cow rumen found that OTUs classified as *Sphingobacteriaceae* were prominent in the rumen fluid during the process [70]. Thus, it is not surprising that these bacteria should be associated with sheep and plant-eating primates, and the fact that they were so highly enriched in geladas relative to the other monkey species, and even sheep, highlights the role of *Sphingobacteriaceae* in the geladas’ unique adaptation to graminivory among primates. The poor classification accuracy achieved for these OTUs demonstrates, however, how little we still know about the contribution of this large and diverse group of bacteria, within the phylum *Bacteroidetes*, to the digestion of plant materials in animal hosts.

16S rRNA sequences classifying to *Verrucomicrobia Subdivision 5* have been found in marine environments [71] and animal digestive systems, including primates [72] and ruminants [73]. However, despite being widespread, this group remains poorly described [74]. Our results suggest that members of *Verrucomicrobia Subdivision 5* may be important contributors to digestive function in both sheep and plant-eating primates.

PICRUSt analysis found that of all the primates in our study, geladas were the most similar to sheep in terms of the composition of KEGG orthologs in the predicted metagenomes. This is not surprising given that the GI microbiotas of geladas and sheep were the most similar. The results of our PICRUSt analysis should, however, be interpreted with caution given the very high NSTI values obtained for geladas. Prediction accuracy has been shown to drop quite sharply with increasing NSTI [30], and thus, it is possible that PICRUSt was unable to produce a realistic representation of the gelada GI metagenome. This result further underlines the uniqueness of the microbial community that forms part of the gelada digestive system. It may be worth mentioning that we observed an apparent gradient in enrichment of KOs dedicated to amino acid metabolism going from graminoid-feeding specialists to ecological generalists. This observation may reflect an increase need for scavenging and synthesizing amino acids in the GI microbiota of animals with reduced protein intake, though additional research will be needed to more thoroughly test this idea.

Our study, while contributing to an emerging literature about the microbiomes of wild animals, also draws attention to the fact that we still know relatively little about many bacterial and archaeal lineages. For example, while there are many recognized and well described species of *Sphingobacteriaceae*, there has been relatively little focus on the members of this bacterial group that are associated with animal GI systems. Moreover, the genus *Fibrobacter*, widely recognized as a key player in ruminant digestion, currently only includes two recognized species, while *Verrucomicrobia Subdivision 5* still lacks a described species. Large-scale efforts to describe genomic diversity across all bacterial and archaeal lineages, based on genome assemblies from shotgun metagenomics data, represent valuable contributions that will help fill in the gaps in our understanding of the natural world of microbes [75]. However, increased efforts toward cultivating species that are not necessarily of medical importance, including those associated with wild animals, will ultimately be needed in order to more fully understand the physiology of the organisms that currently make up the microbial dark matter [76]. These efforts will also strengthen future studies using metagenomics techniques and help us gain a better understanding of important natural processes like cellulose degradation.

## Conclusions

Here, we have presented the first study of the GI microbiota of the gelada monkey, the sole surviving species in the *Theropithecus* lineage and the only living primate graminivore. Although geladas are listed as a species of least concern by the IUCN, their rapidly dwindling Afroalpine grassland habitats are under increasing pressure from massive human population growth, agriculture, livestock grazing, and climate change across the Ethiopian Highlands [15]. Thus, it is timely that we gain insight into the digestive system that enables these monkeys to subsist on a graminoid-based diet. This study extends our knowledge about the GI microbiota adaptations required for graminivory, as well as the behavioral and environmental factors that help shape the GI micriobiota of these unique social primates.

## Methods

### Study site

The Guassa Plateau (10° 15′–10° 27′ N; 39° 45′–39° 49′ E) covers 111 km^2^ along the western edge of the Great Rift Valley in the central Ethiopian Highlands at altitudes ranging from 3200 to 3600 m.a.s.l. Guassa is characterized by intact Afroalpine vegetation consisting largely of tall graminoids, forbs, and shrubs [13]. Rainfall averages 1650 ± 243 mm annually with the wet season lasting from July–October and the dry season from November–June [13, 17]. Mean daily low temperatures average 4.3 ± 0.5 °C while mean daily high temperatures average 17.8 ± 0.3 °C^12^. Guassa is home to several gelada bands, including one consisting of ~ 220 members (Steelers Band), which has been followed by researchers on a near-daily basis since January 2007. The area is protected by an ancient indigenous conservation system, and it is also home to a large and varied carnivore community, including leopards (*Panthera pardus*), spotted hyenas (*Crocuta crocuta*), Ethiopian wolves (*Canis simensis*), African wolves (*Canis lupaster*) and servals (*Leptailurus serval*) [77].

### Sampling

We collected 316 gelada monkey fecal samples during two 6-week periods, one in the dry season (March–April 2016, *n* = 142) and the other in the wet season (September–October 2016, *n* = 174). Thirty-nine individually recognized animals, all females of reproductive age, were sampled, with each animal sampled 1–12 times at a mean of eight samples per individual. All individuals belonged to one of eight different reproductive units from a single band. The median unit size was six females (range two to nine), and the number of samples collected from each unit ranged from 8 to 84 (mean = 40). Nine of the 39 individuals sampled were classified as old, meaning that they were adults at the time when monitoring of individually known geladas began 10 years earlier. From these monkeys, we obtained a total of 70 samples, while the remaining 246 samples were from individuals that were classified as prime, meaning that they reached sexual maturity during the study period.

Gelada females go through a 6-month gestation period [78], and each fecal sample was classified according to the reproductive status of the defecating individual at the time of collection. Individuals were classified either as pregnant in the first (*n* = 27), second (*n* = 13), or third (*n* = 32) trimester, cycling (i.e., reproductively receptive, *n* = 66) or going through postpartum amenorrhea (i.e., postnatal infertility, *n*=178). Eleven of the females had large subcutaneous swellings (coenurosis, *n* = 81) caused by the parasitic tapeworm *Taenia serialis* (see Nguyen et al. [26] for more details), while the remaining 28 animals (*n* = 235) did not show visible symptoms. For each gelada fecal sample, approximately 2–3 g of feces was transferred to a 15 ml sterile plastic vial with ~ 6–7 ml 96% ethanol, immediately upon defecation. Sample tubes were then stored at ambient temperature at the field research camp prior to transport to the University of Oslo, Norway, for further analysis.

We collected 29 fecal samples from a Bale monkey population in Odobullu Forest, an intact bamboo forest with minimal human disturbance in the Bale mountains of southern Ethiopia [27]. These samples were collected from October 2013 to May 2014, encompassing both wet and dry periods. We also sampled a vervet population (11 samples) living in the rural area of Sof Omar directly east of the Bale mountains, and a grivet population (13 samples) from the area around the city of Awassa west of the Bale mountains. Both Sof Omar and Awassa represent habitats with a high degree of human influence, including agriculture, livestock grazing, and tourism (A. Mekonnen, pers. observ.). The Awassa samples were collected in April 2016, while Sof Omar samples were collected in July of the same year. As with the gelada samples, Bale monkey, vervet, and grivet fecal samples were stored in tubes with 96% ethanol prior to transport to Oslo for further processing and analysis.

Human fecal samples were obtained from 11 adult volunteers (30–40 years old) and 10 infants of approximately 1 year of age in Oslo, Norway. All samples were collected within a 1-week period and frozen directly upon collection pending further processing. At the time of sampling, none of the human volunteers were using antibiotics or were known to suffer from any serious disease or immunocompromised status.

Sheep rumen content samples were obtained from 29 freshly slaughtered animals at Furuseth AS slaughterhouse outside of Oslo in mid-January 2016. The sheep were approximately 1 year old and came from three different farms in the Gudbrandsdalen area in central Norway. Norwegian sheep farming relies on extensive use of natural open range pastures during spring and summer, where nearly half of the total annual feed is consumed. Rumen samples were frozen on dry ice directly upon collection and transported to a storage facility at the University of Oslo.

### DNA extraction and sequencing

DNA extraction from all samples was carried out with the PowerSoil 96 well DNA isolation kit (MO BIO Laboratories Inc., Carlsbad, CA, USA), per instructions provided by the manufacturer. Library preparation for Illumina sequencing of the V4 region of the 16S rRNA gene was carried out according to de Muinck et al. [34]. Sequencing was carried out on an Illumina HiSeq 2500 apparatus (Illumina, San Diego, CA, USA) using the 2x250PE rapid run mode and 10% PhiX spike-in. For the geladas, the mean per sample read number was 170,362 (± 75,585 s.d.) after quality trimming, paired read merging, and chimera removal. The corresponding numbers were 137,277 (± 31,096) for Bale monkeys, 132,994 (± 24,038) for vervets, 133,241 (± 29,109) for grivets, 151,282 (± 26,206) for human adults, 170,492 (± 34,179) for human infants, and 151,589 (± 32,949) for sheep.

### Data processing and statistical analyses

Low quality reads were trimmed and Illumina adapters were removed using Trimmomatic v0.36 [79] with default settings. Reads mapping to the PhiX genome (NCBI id: NC_001422.1) were removed using BBMap v36.02 [80]. De-multiplexing of data based on the dual index sequences was carried out using custom scripts [34]. Internal barcodes and spacers were removed using cutadapt v1.4.1 [81], and paired reads were merged using FLASH v1.2.11 [82] with default settings.

Further processing of sequence data was carried out using a combination of vsearch v2.0.3 [83] and usearch v9.2.64 [84]. Specifically, dereplication was performed with the “derep_fulllength” function in vsearch with the minimum unique group size set to 2. Operational taxonomic unit (OTU) clustering, chimera removal, taxonomic assignment, and OTU table building were carried out using the uparse pipeline [85] in usearch. OTU clustering was carried out in one step sing the entire data set. Taxonomic assignment to the genus level was done against the RDP-15 training set. OTUs with a domain-level assignment probability < 0.95 were removed as likely artifacts. OTUs classified as chloroplast 16S rRNA genes were also excluded from further analysis. Classification to the species level was done by BLASTing [86] against the GenBank 16S rRNA gene database (query coverage ≥ 99%, *e*-value < 1e-125). Between-sample differences in sequencing library size were normalized by common scaling [87]. This entails multiplying all OTU counts for a given library with the ratio of the smallest library size in the entire data set to the size of the individual library. This procedure replaces rarefying (i.e., random sub-sampling to the lowest number of reads) as it produces the library scaling one would achieve by averaging over an infinite number of repeated sub-samplings. Library size scaling was carried out using a smallest library size of 50,662 reads. After scaling, the data were filtered to retain only OTUs with at least 0.01% relative abundance in at least one sample (i.e., at least five reads), in order to eliminate OTU artifacts. This filtering step was done separately depending on the comparisons being made (e.g., geladas vs. sheep, sheep vs. all primates etc.).

All statistical tests were done in R [88]. We used Permutational Multivariate Analysis of Variance Using Distance Matrices (PERMANOVA) to evaluate the effects of a number of important extrinsic and intrinsic variables on the gelada GI microbiota, including (a) social unit membership (1–8), (b) age group, (c) reproductive condition, (d) parasitic tapeworm disease status, as well as (e) season of sample collection. PERMANOVA tests were carried out using the “adonis” function in the “vegan” package, using Bray-Curtis or UniFrac dissimilarities and 1000 permutations. To account for pseudoreplication, the individual identifiers of the monkeys were included as a blocking variable in the “strata” argument of the “adonis” function. This was done for all PERMANOVA tests except the one for individual effects. Non-metric multidimensional scaling (NMDS) of Bray Curtis distance matrices was carried out using the “isoMDS” function in the “MASS” package. Exact tests for differences in means between two groups of negative binomially distributed counts were carried out using the edgeR package [89]. Although originally developed for analysis of differential expression in RNA sequencing experiments, this method has been shown to perform well in identifying enriched OTUs in 16S rRNA amplicon sequencing experiments as well [87]. In order to focus on the most prevalent OTUs, the exact test comparing geladas to other primates was carried out on OTUs that were found at ≥ 0.1% relative abundance in ≥ 10 individuals (the sample size of the smallest group that was included in the comparison) in the combined data set. For the test comparing gelada in the dry and wet seasons, we included OTUs that were found at ≥ 0.1% relative abundance in ≥ 79 samples (25% of the total gelada sample number). An OTU was considered enriched if both the *p* value and false discovery rate were < 0.01. UniFrac distances were computed using the “GUniFrac” function in the “GUniFrac” package [90]. For constructing the phylogenies upon which the Unifrac distances were based, sequences were aligned using MUSCLE [91] and a neighbor joining tree [92] was constructed using MEGA v7.0.26 [93]. The phylogeny of OTUs classified to the phylum Fibrobacteres was constructed in the same fashion. Metagenomes were predicted using Phylogenetic Investigation of Communities by Reconstruction of Unobserved States (PICRUSt 1.1.1) [30] after normalizing for 16S rRNA gene copy number. Oligotyping was done using the DADA2 R-package [29] with default parameters. DADA2 recognized 58,584 sequence variants, and for analysis of the data, the abundance table was filtered to retain only sequence variants that were observed with at least 50 reads in at least one sample. Finally, all comparisons above the OTU level were done excluding OTUs that could not be classified to the phylum level with an assignment probability over 0.5.

## Additional file

Additional file 1: The document contains Figs. S1-S17 and Tables S1-S10. (PDF 502 kb)
