# Supplementary material for: Multilevel social structure and diet shape the gut microbiota of the gelada monkey, the only grazing primate
Source: Microbiome. 2018 May 5;6:84. doi: 10.1186/s40168-018-0468-6 (PMC5935910; doi:10.1186/s40168-018-0468-6)
Supplement: Supplementary file 1 — The document contains Figs. S1-S17 and Tables S1-S10. (PDF 502 kb) [file 40168_2018_468_MOESM1_ESM.pdf]

Multilevel social structure and diet shape the gut microbiota of the gelada monkey, the only grazing primate

Pål Trosvik[1*], Eric J. de Muinck[1], Eli K. Rueness[1], Peter J. Fashing[2], Evan C. Beierschmitt[3], Kadie R. Callingham[4], Jacob B. Kraus[5], Thomas H. Trew[6], Amera Moges[7], Addisu Mekonnen[1,8], Vivek V. Venkataraman[9], Nga Nguyen[2]

Supplementary information: Supplementary Figures 1-17, Supplementary Tables 1-10.

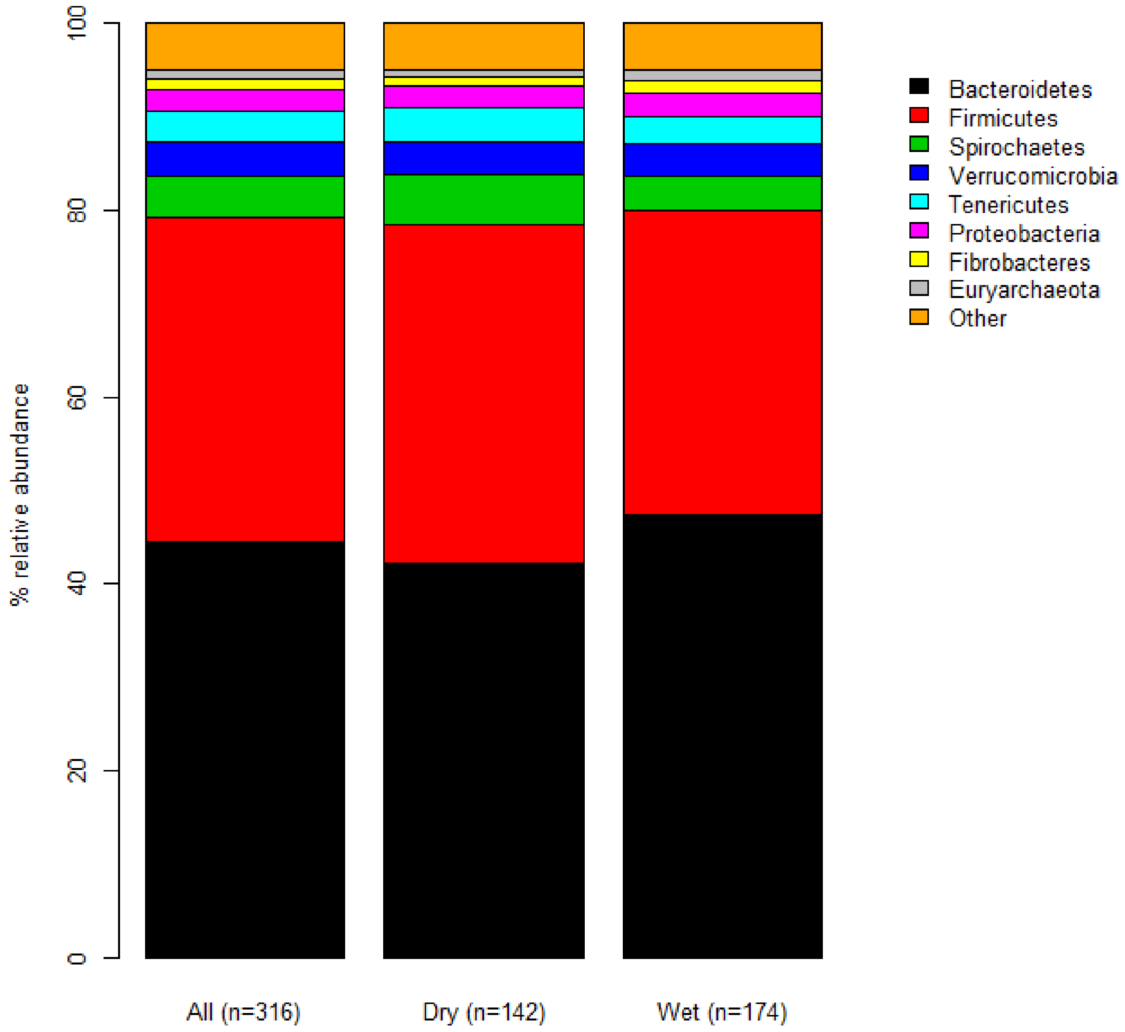

Figure S1. Relative abundances of the eight most prevalent phyla in the gelada samples. Data are shown for all samples combined, as well as split into samples collected during the dry or wet season. The category "Other" includes OTUs that could not be classified to the phylum level with a probability higher than 0.5.

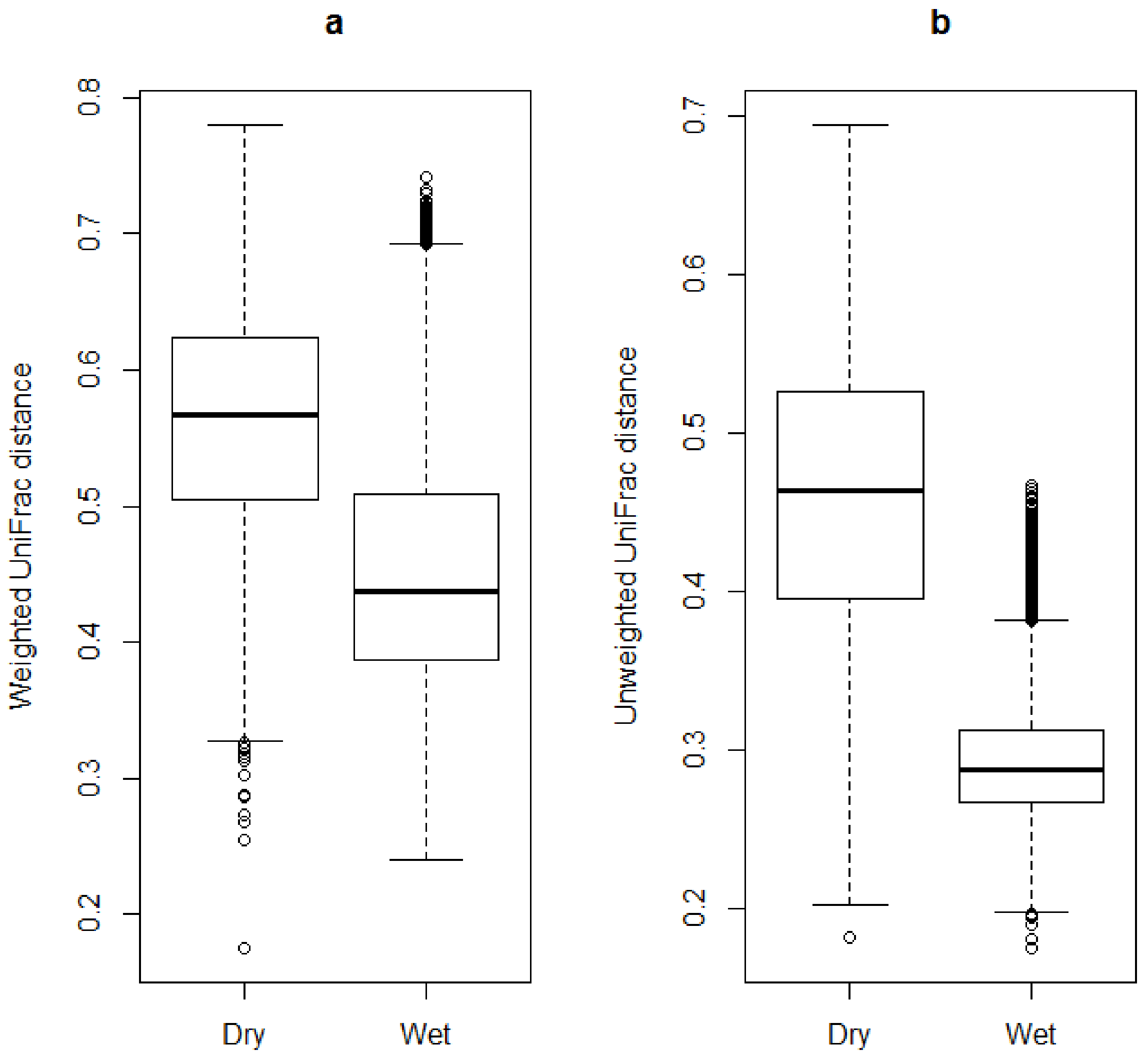

Figure S2. Between-sample weighted (a) and unweighted (b) UniFrac distances in gelada samples collected during the dry (n=142) or the wet (n=174) season. Each box represents the interquartile range, with the horizontal lines representing the medians and the whiskers representing 1.5 times the interquartile range. Points outside the whiskers represent outliers. For both comparisons the difference in mean distance was highly significant ($t<<0.001$ for both comparisons, unpaired t-tests).

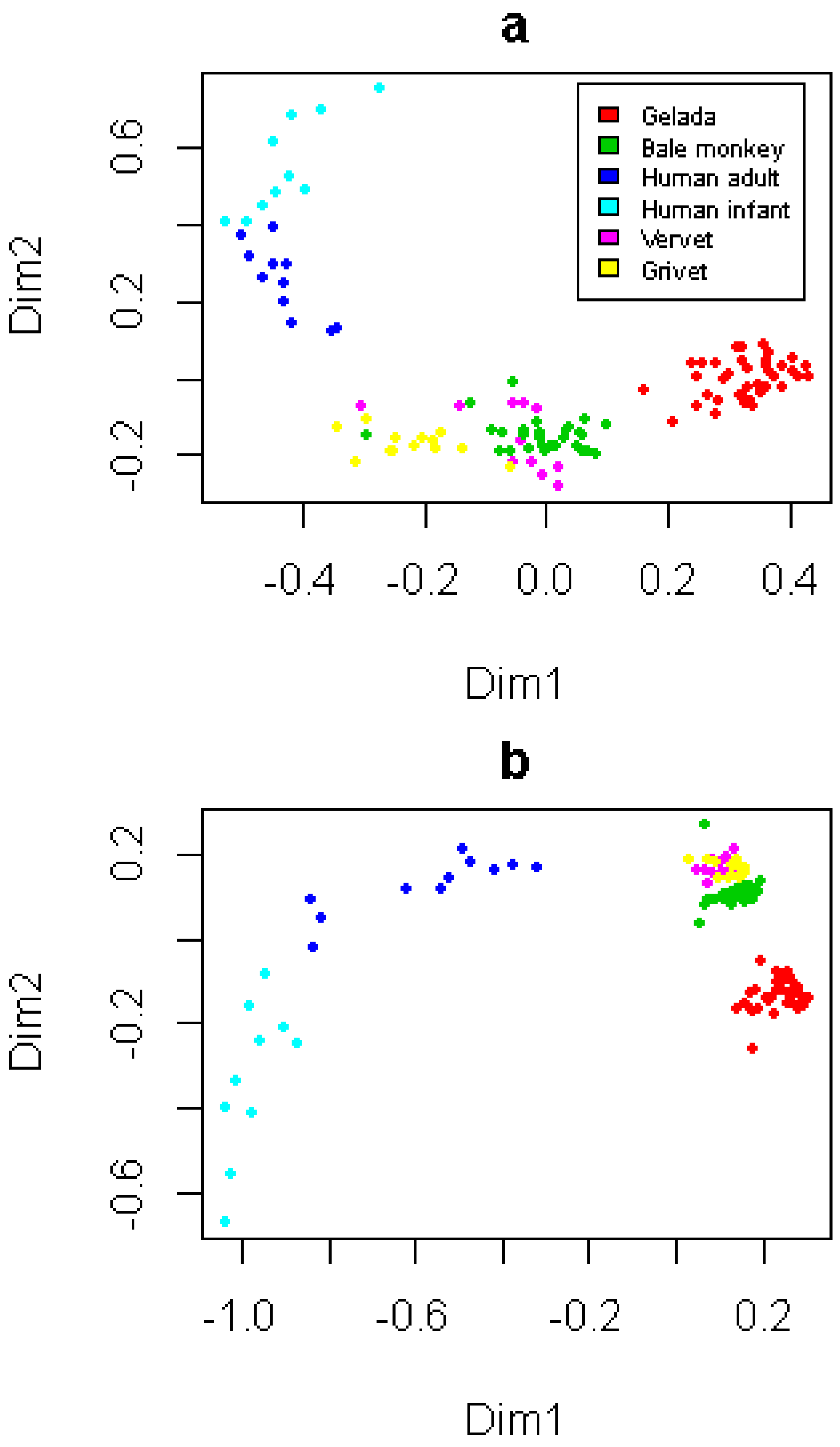

Figure S3. Non-metric multidimensional scaling of all primate samples based on weighted (a) and unweighted (b) UniFrac distances. The plot shows the two main dimensions of variation, with plotted characters color coded according to sample type. Clustering according to samples type was highly significant, explaining 46.2% and 63.1% of between-sample variation, respectively ($p<<0.001$ for both tests, PERMANOVA). The model stress values were 9.26 and 5.32, respectively. Gelada (n=39), Bale monkey (n=29), human adult (n=11), human infant (n=10), vervet (n=11), grivet (n=13).

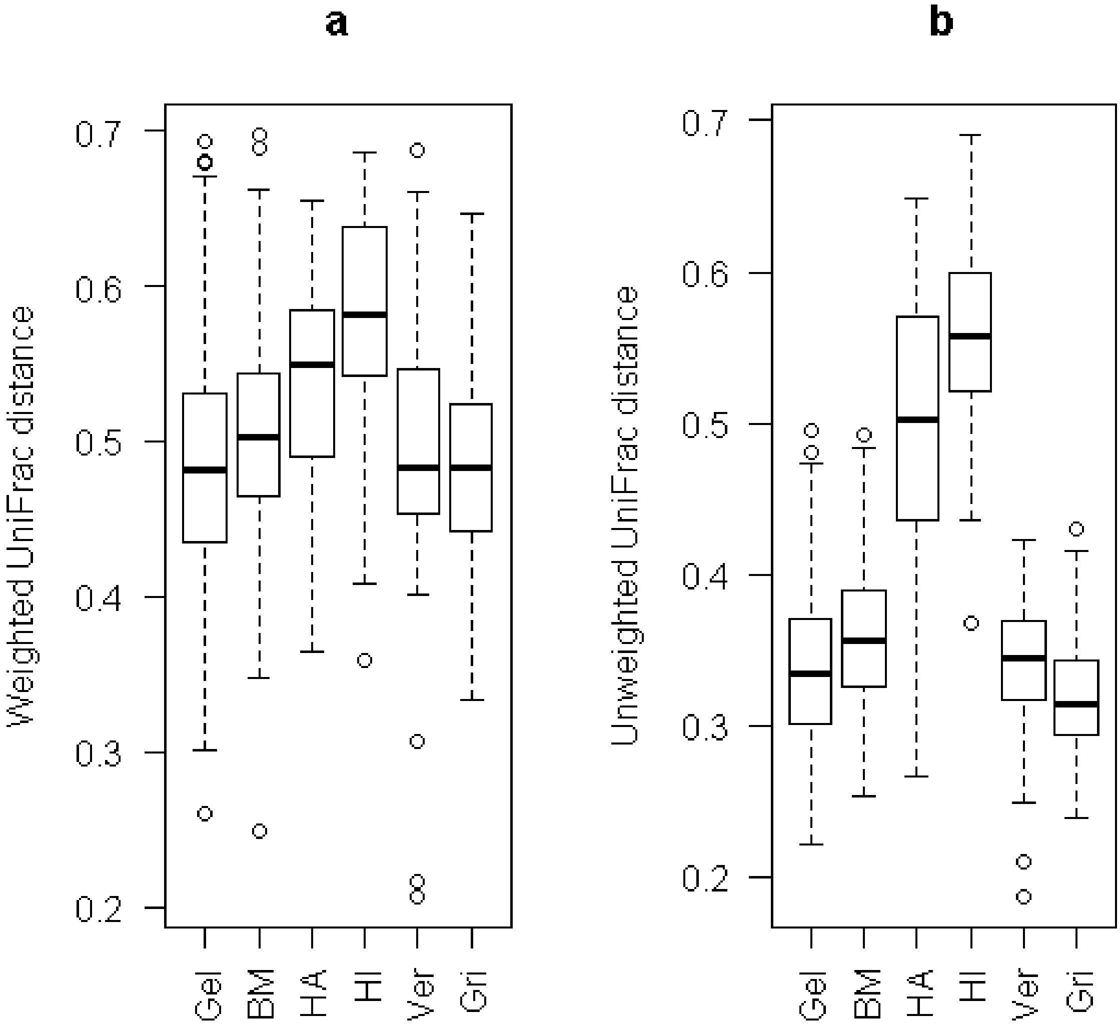

Figure S4. Differences in weighted (a) and unweighted (b) UniFrac distances within each primate group. Each box represents the interquartile range, with the horizontal lines representing the medians and the whiskers representing 1.5 times the interquartile range. Points outside the whiskers represent outliers. For both metrics the mean within-group distance was significantly less in geladas than Bale monkeys, human adults and infants ($p<0.001$ for all comparisons, unpaired t-tests), but not from vervets and grivets. Also, for both metrics mean distances were higher within human adults and infants relative to all monkey species ($p<0.001$ for all comparisons). Gel=gelada (n=39), BM=Bale monkey (n=29), HA=human adult (n=11), HI=human infant (n=10), Ver=vervet (n=11), Gri=grivet (n=13).

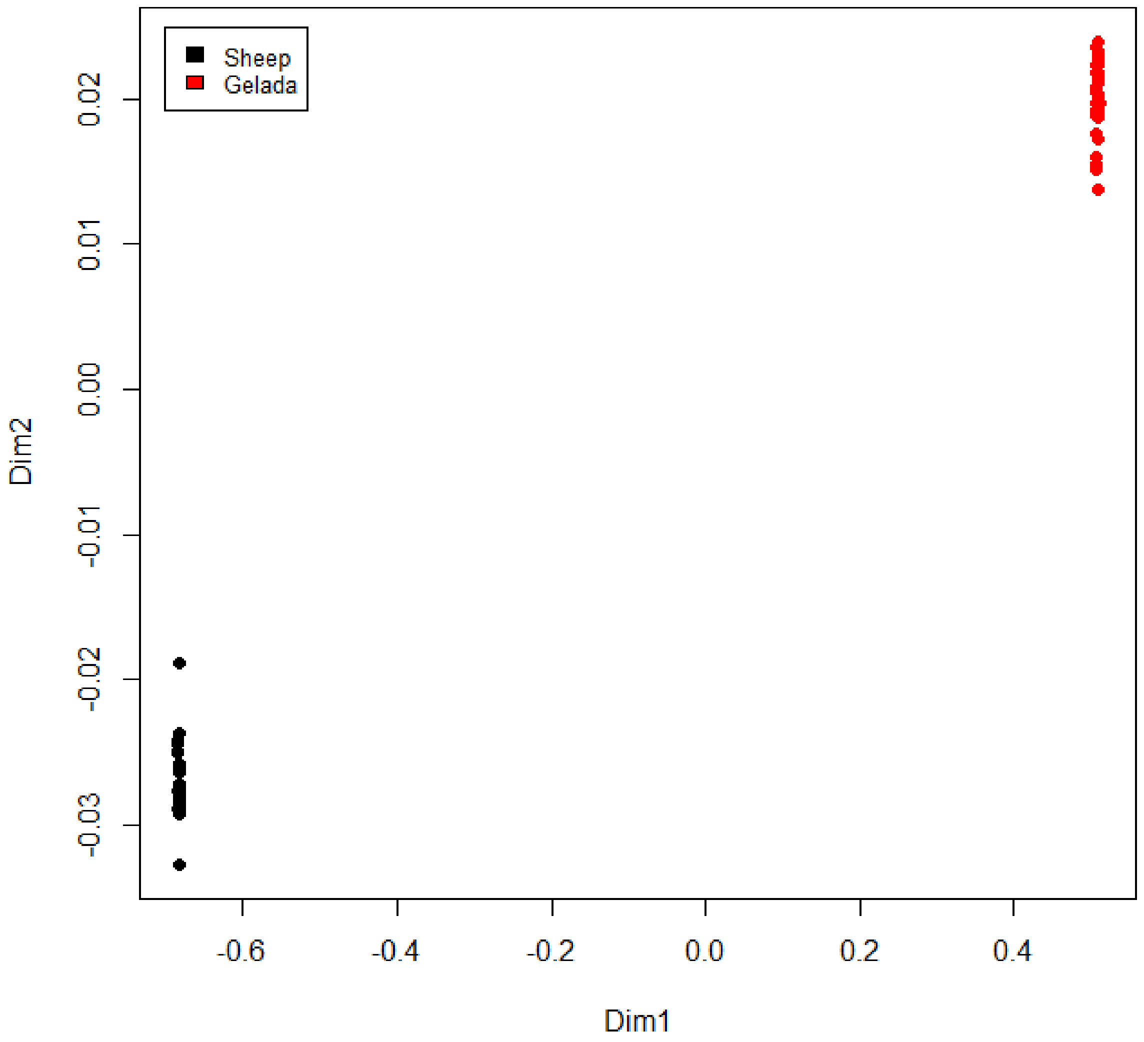

Figure S5. Non-metric multidimensional scaling of gelada (n=39) and sheep (n=29) samples based on the Bray-Curtis distance matrix. The plot shows the two main dimensions of variation, with plotted characters color-coded according to sample type. Clustering according to samples type was highly significant, explaining 48% of between-sample variation (p<<0.001, PERMANOVA). The model stress was 0.17.

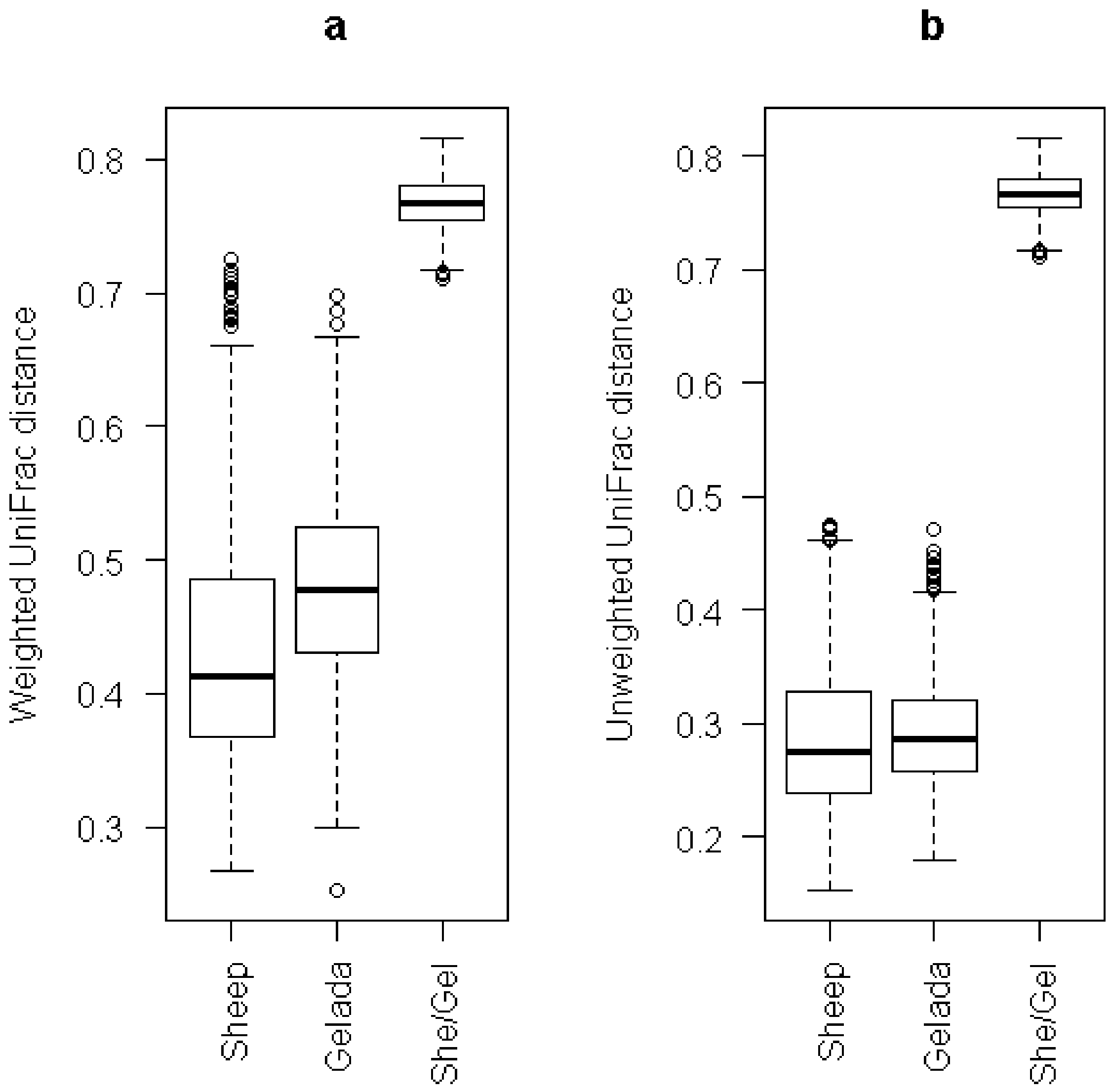

Figure S6. Weighted (a) and unweighted (b) UniFrac distances within sheep (n=29) and geladas (n=39), as well as distances between sheep and gelada samples (She/Gel). Each box represents the interquartile range, with the horizontal lines representing the medians and the whiskers representing 1.5 times the interquartile range. Points outside the whiskers represent outliers. In panel (a) the mean distance within the sheep group was significantly less than within the geladas, while it was less within both groups than between the groups ($p<0.001$ for all comparisons, unpaired t-tests). In panel (b) the mean distance was less within both groups than between the groups ($p<0.001$), but within-group distances did not differ significantly between sheep and geladas.

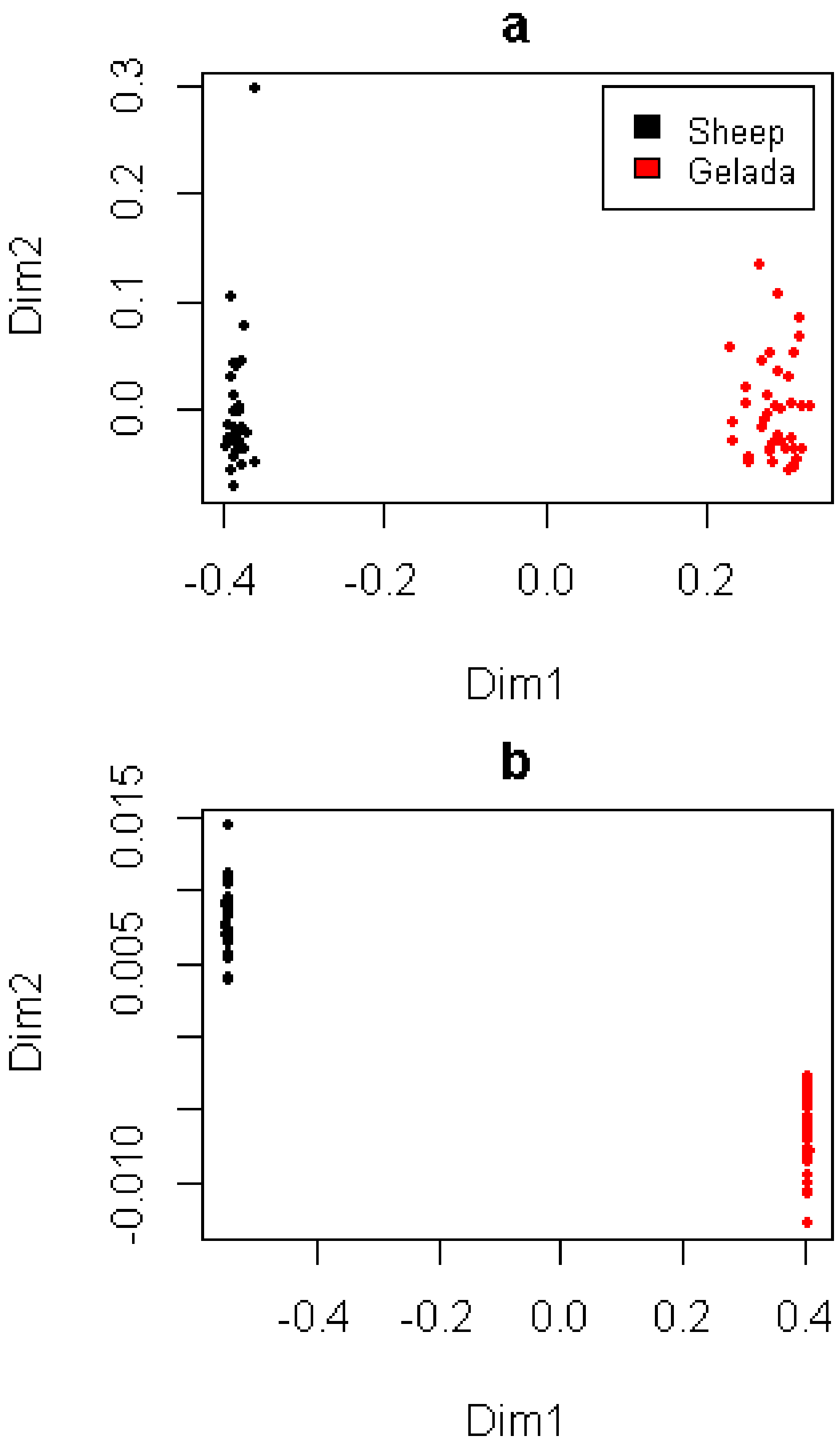

Figure S7. Non-metric multidimensional scaling of gelada (n=39) and sheep (n=29) samples based on weighted (a) and unweighted (b) UniFrac distances. The plot shows the two main dimensions of variation, with plotted characters color-coded according to sample type. Clustering according to samples type was highly significant, explaining 46.3% and 76.3% of between-sample variation, respectively ($p<<0.001$ for both tests, PERMANOVA). The model stress values were 3.94 and 0.22, respectively.

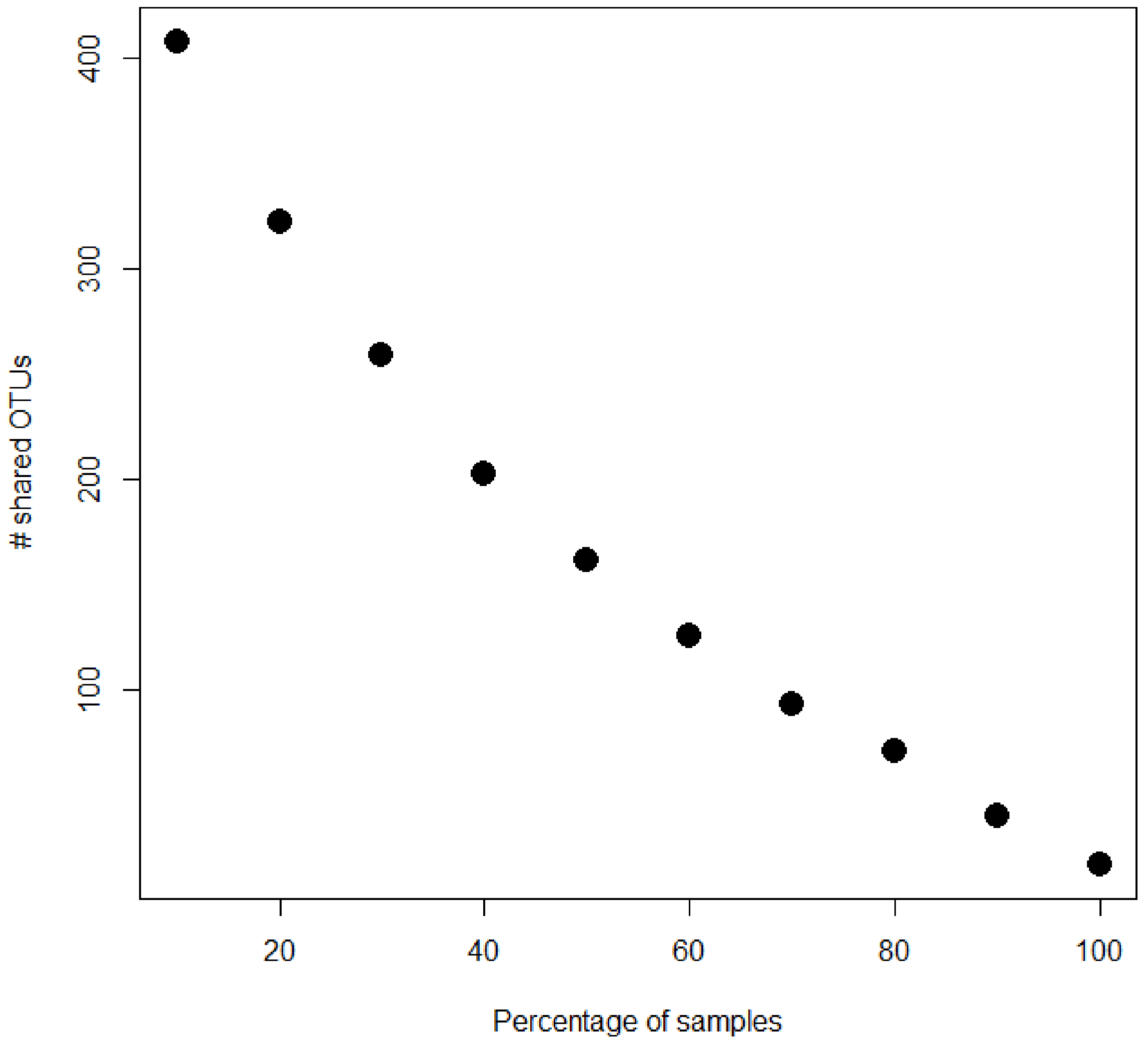

Figure S8. The number of OTUs shared by at least the percentage of samples, from both gelada monkeys and sheep, indicated on the x-axis. E.g. the dot in the top left corner indicates the number of OTUs observed in at least 10% of the geladas and at least 10% of the sheep.

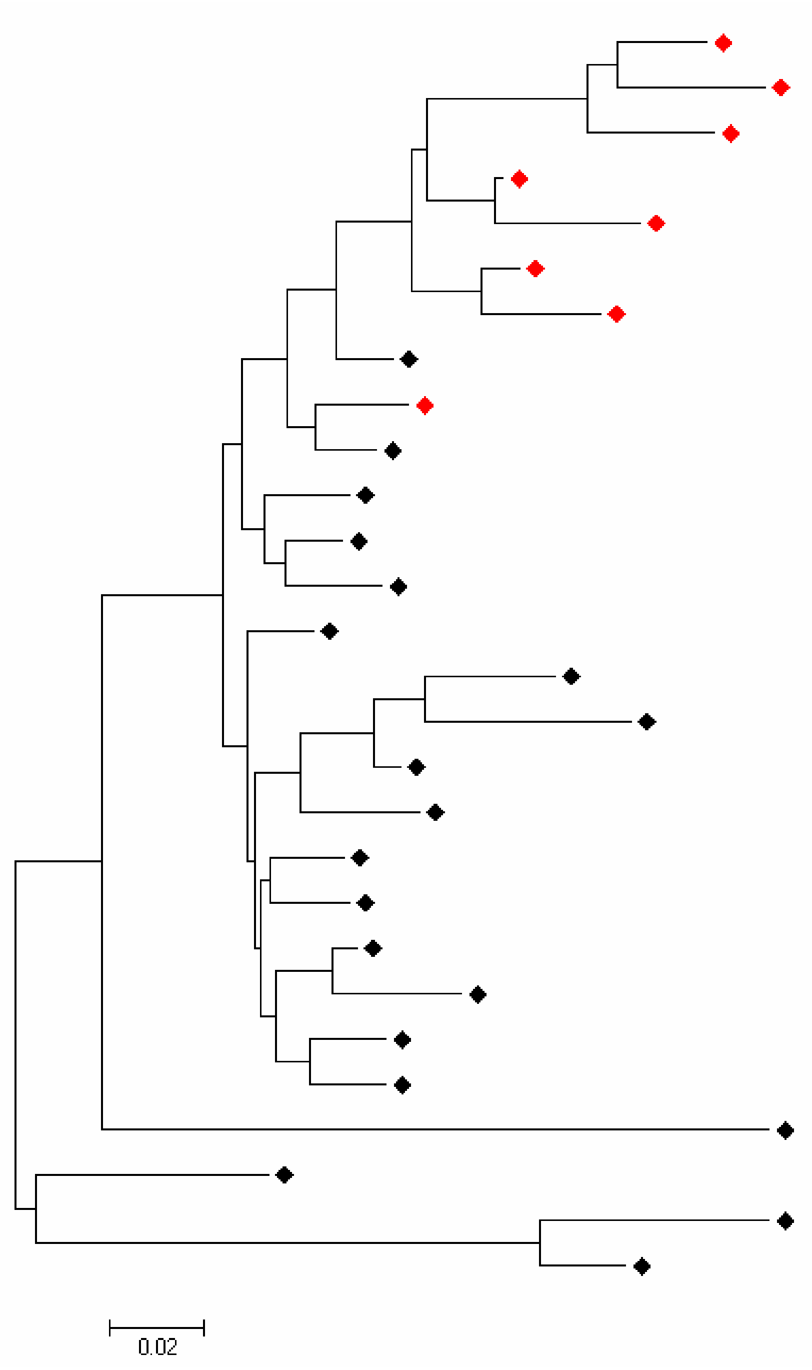

Figure S9. Phylogeny of the 28 OTUs classified as Fibrobacteres. The evolutionary history was inferred using the Neighbor-Joining method. The evolutionary distances are in the units of the number of base substitutions per site. Estimating the phylogeny using the Maximum Likelihood or Minimum Evolution method resulted in identical tree topologies. Red and black diamonds are OTUs found exclusively in geladas and sheep, respectively.

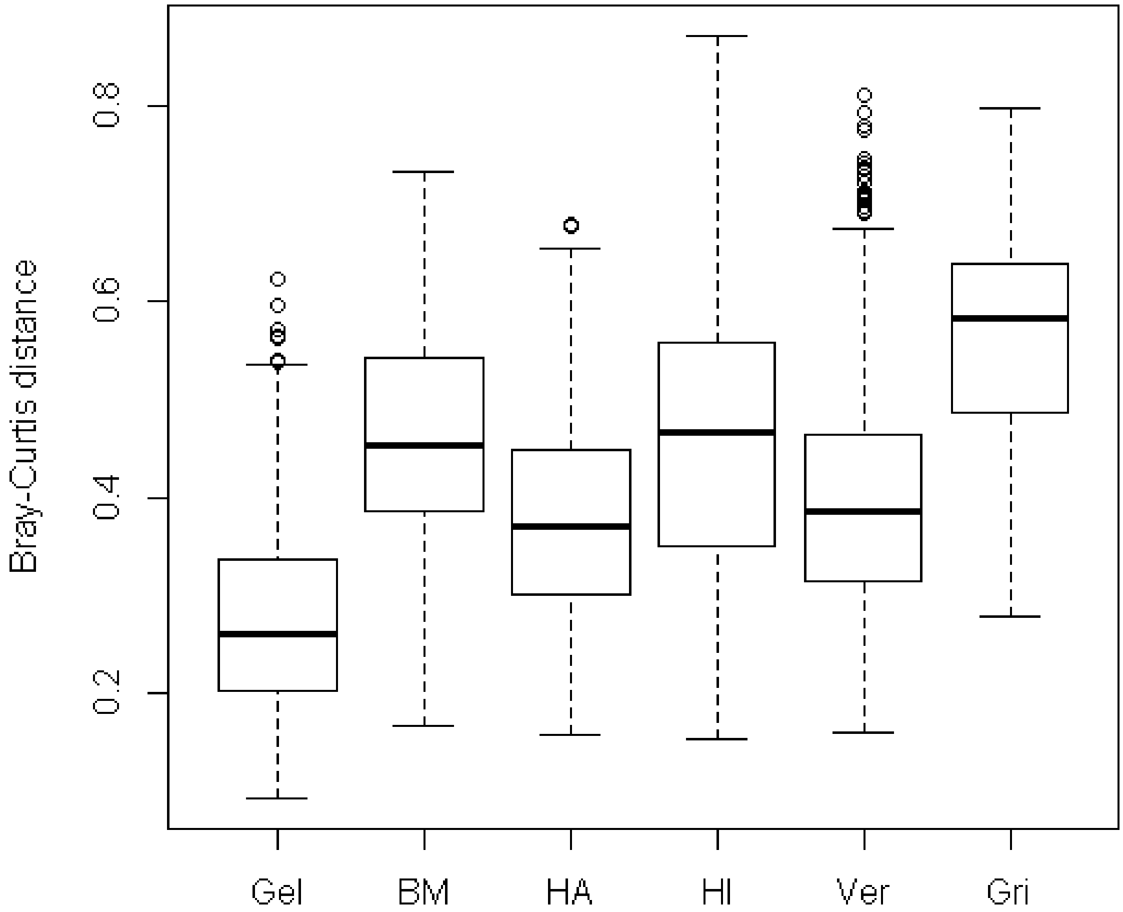

Figure S10. Phylum level Bray-Curtis distances between sheep (n=29) and all primate groups for OTUs that could be classified to the phylum level at a probability of at least 0.5 (90.2% of all OTUs). For all comparisons the mean distance is less for geladas than the other groups (p<0.001, unpaired t-tests). The analysis only included OTUs that could be assigned to a phylum with a probability >0.5. Each box represents the interquartile range, with the horizontal lines representing the medians and the whiskers representing 1.5 times the interquartile range. Points outside the whiskers represent outliers. Gel=gelada (n=39), BM=Bale monkey (n=29), HA=human adult (n=11), HI=human infant (n=10), Ver=vervet (n=11), Gri=grivet (n=13).

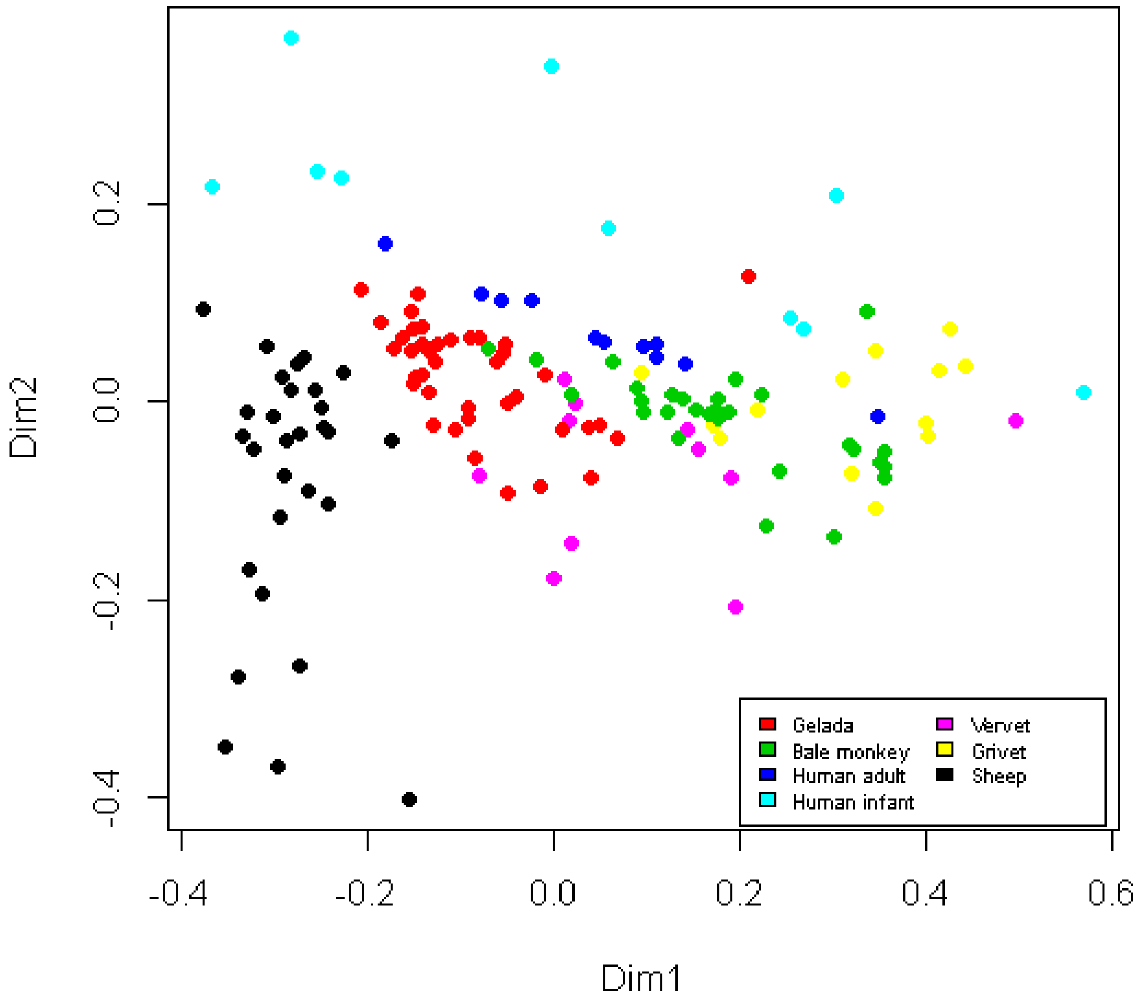

Figure S11. Non-metric multidimensional scaling of primate and sheep samples based on phylum level Bray-Curtis distances for OTUs that could be classified to the phylum level at a probability of at least 0.5 (90.2% of all OTUs). The plot shows the two main dimensions of variation, with plotted characters color-coded according to sample type. The model stress value was 8.4. The analysis only included OTUs that could be assigned to a phylum with a probability >0.5. Gelada (n=39), Bale monkey (n=29), human adult (n=11), human infant (n=10), vervet (n=11), grivet (n=13), sheep (n=29).

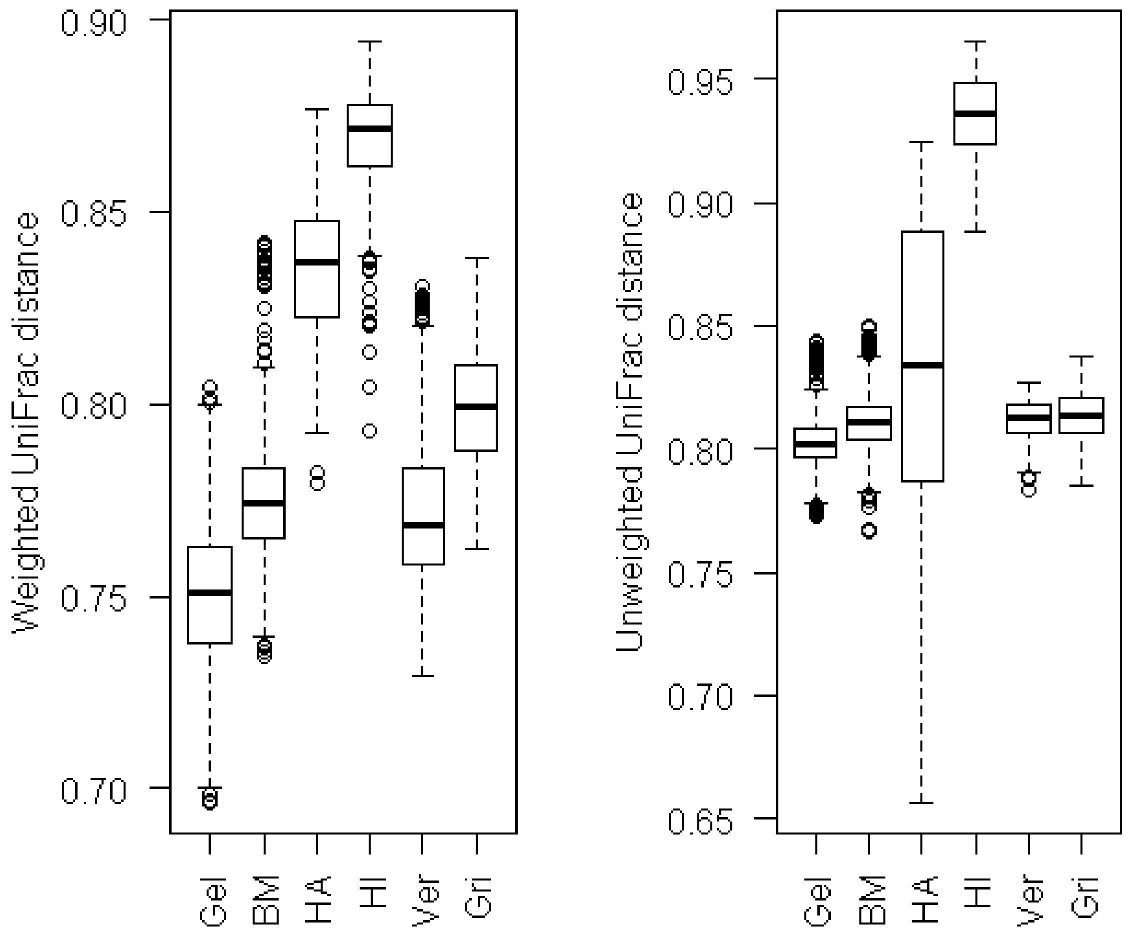

Figure S12. Weighted (a) and unweighted (b) UniFrac distances between sheep (n=29) and all the primates in our study. In both panels the mean distance is significantly less for geladas than for any other primate grouping (p<0.001 for all comparisons, unpaired t-tests). In each panel the mean distance is higher in human adults than all the monkey species, and highest in human infants relative to all other groups (p<0.001 for all comparisons). Each box represents the interquartile range, with the horizontal lines representing the medians and the whiskers representing 1.5 times the interquartile range. Points outside the whiskers represent outliers. Gel=gelada (n=39), BM=Bale monkey (N=29), HA=human adult (n=11), HI=human infant (n=10), Ver=vervet (n=11), Gri=grivet (n=13).

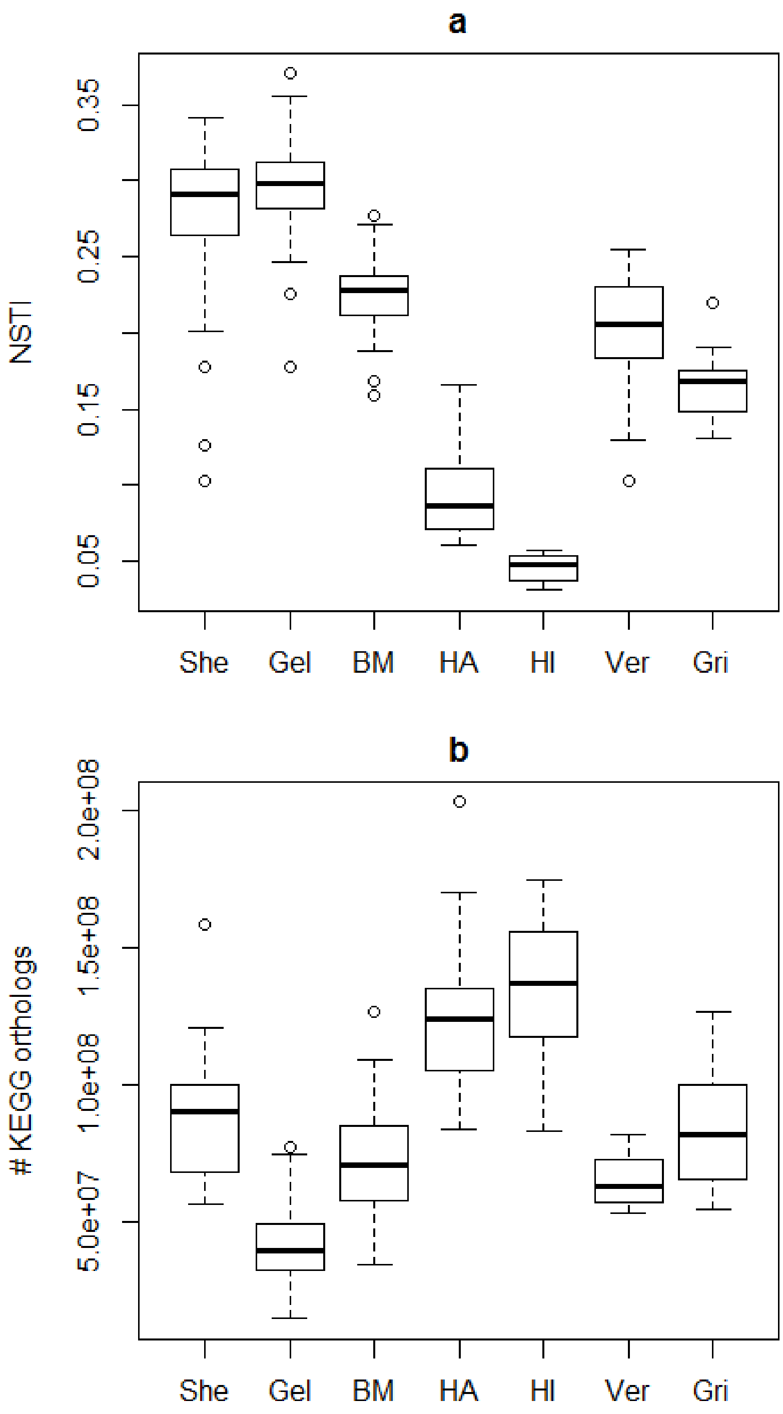

Figure S13. (a) Nearest Sequenced Taxon Index (NSTI) and (b) total number of assigned KEGG orthologs from PICRUSt analysis. The mean NSTI for geladas is significantly higher than for the other primates ($p<0.001$). The mean number of KEGG orthologs is significantly lower for geladas than the other groups ($p<0.001$)Each box represents the interquartile range, with the horizontal lines representing the medians and the whiskers representing 1.5 times the interquartile range. Points outside the whiskers represent outliers. She=sheep (n=29), Gel=gelada (n=39), BM=Bale monkey (N=29), HA=human adult (n=11), HI=human infant (n=10), Ver=vervet (n=11), Gri=grivet (n=13).

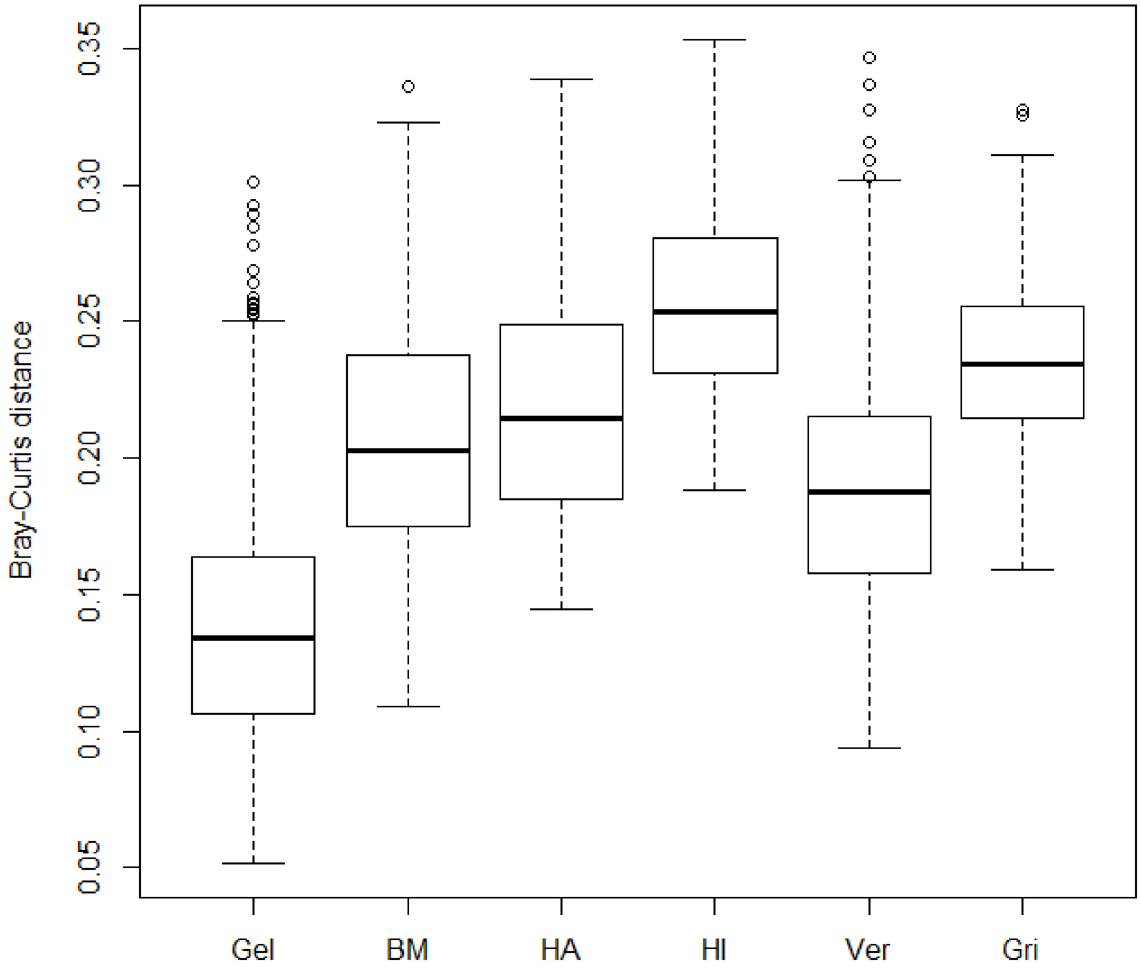

Figure S14. Bray-Curtis distances between sheep (n=29) and all primate groups based on the KEGG ortholog relative abundance table. For all comparisons the mean distance is less for geladas than the other groups ($p<0.001$, unpaired t-tests). Each box represents the interquartile range, with the horizontal lines representing the medians and the whiskers representing 1.5 times the interquartile range. Points outside the whiskers represent outliers. Gel=gelada (n=39), BM=Bale monkey (n=29), HA=human adult (n=11), HI=human infant (n=10), Ver=vervet (n=11), Gri=grivet (n=13).

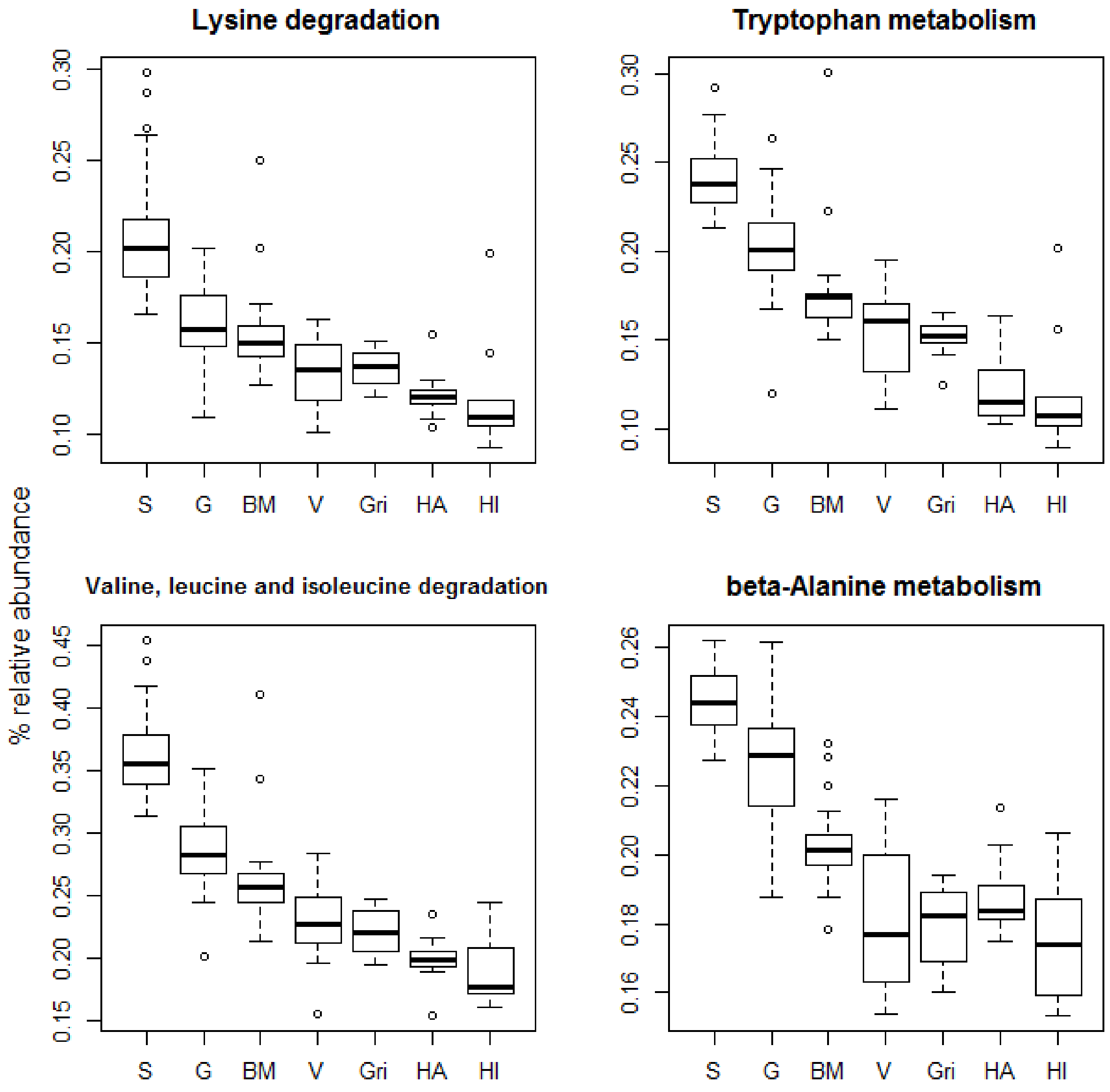

Figure S15. Examples of KEGG pathways, involving amino acid metabolism, that show a gradient from grass feeding specialists to dietary generalists. The y-axes indicate the percent relative abundance of KEGG orthologs dedicated to the functional category shown above each plot. Each box represents the interquartile range, with the horizontal lines representing the medians and the whiskers representing 1.5 times the interquartile range. Points outside the whiskers represent outliers. S=sheep (n=29), G=gelada (n=39), BM=Bale monkey (N=29), V=vervet (n=11), Gri=grivet (n=13), HA=human adult (n=11), HI=human infant (n=10).

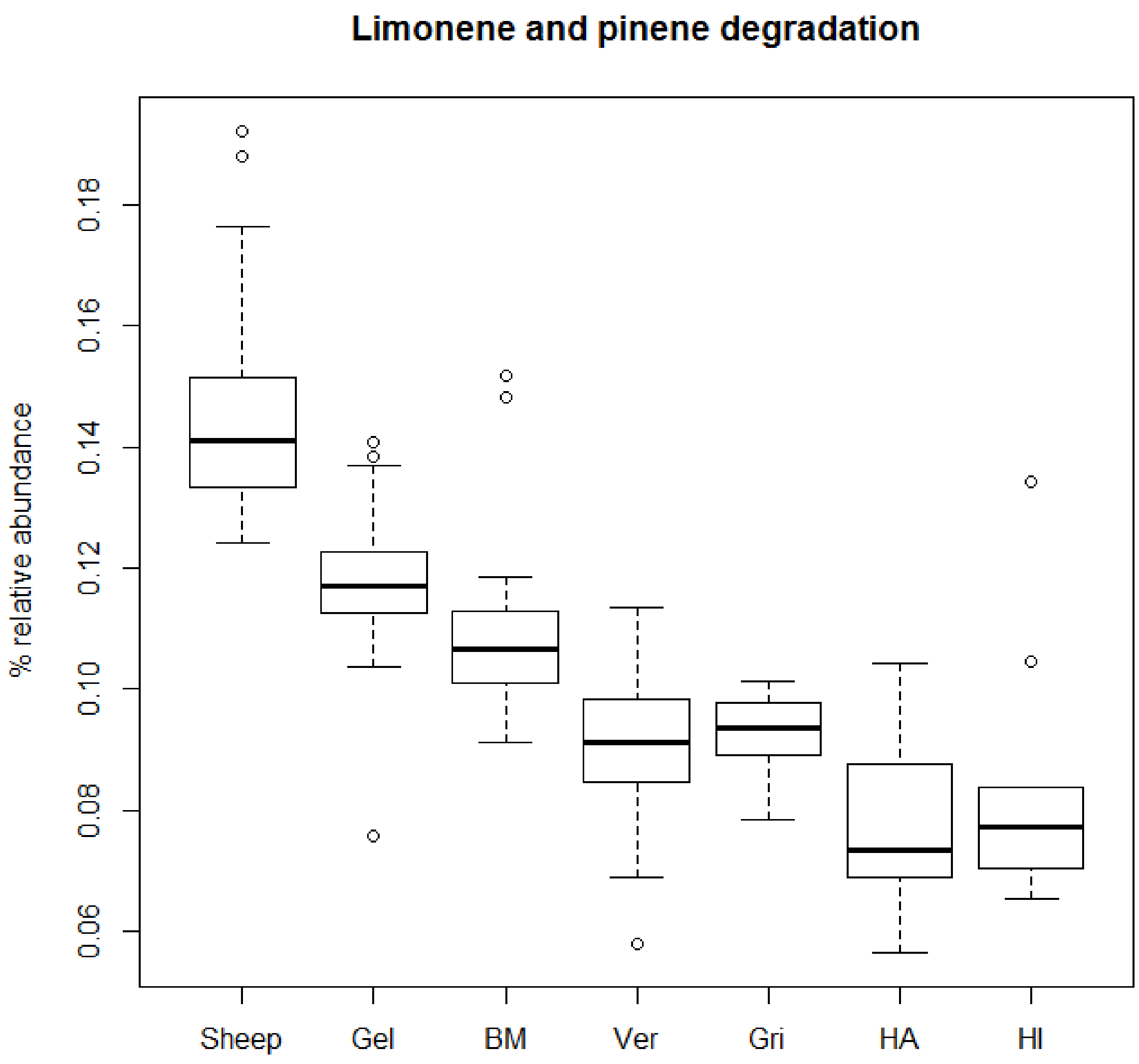

Figure S16. Percent relative abundance of KEGG orthologs dedicated to degradation of limonene and pinene. Each box represents the interquartile range, with the horizontal lines representing the medians and the whiskers representing 1.5 times the interquartile range. Points outside the whiskers represent outliers. S=heep (n=29) , G=gelada (n=39), BM=Bale monkey (N=29), V=vervet (n=11), G=grivet (n=13), HA=human adult (n=11), HI=human infant (n=10).

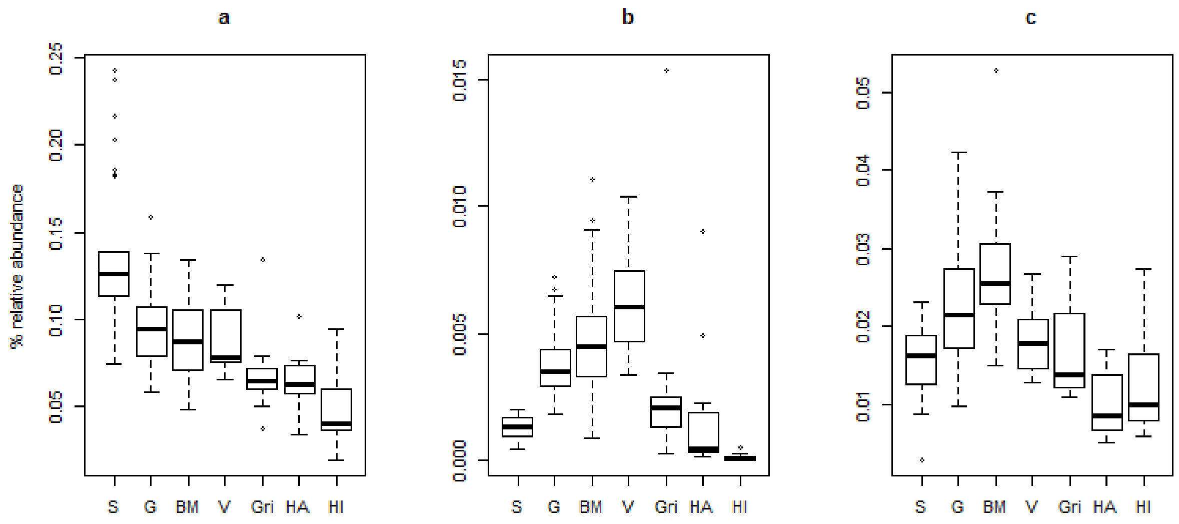

Figure S17. Percent relative abundance of KEGG orthologs for (a) ß-1,4-endoglucanases, (b) ß-1,4-exoglucanases, and (c) beta-glucosidases. ß-1,4-endoglucanases were significantly enriched in sheep relative to all other groups, and in geladas relative to grivets and humans. β-1,4-exoglucanases were enriched in geladas relative to sheep and humans ($p<0.001$), but not relative to other monkey species. Beta-glucosidases were enriched in geladas relative to sheep and all primate groups ($p<0.02$) except Bale monkeys. Each box represents the interquartile range, with the horizontal lines representing the medians and the whiskers representing 1.5 times the interquartile range. Points outside the whiskers represent outliers. S=sheep (n=29), G=gelada (n=39), BM=Bale monkey (N=29), V=vervet (n=11), Gri=grivet (n=13), HA=human adult (n=11), HI=human infant (n=10).

Table S1

Putative taxonomy of OTUs found in all gelada samples (core microbiota). Taxonomic assignment probabilities are in parenthesis.

d:Bacteria(1.0000),p:Bacteroidetes(0.9802),c:Sphingobacteriia(0.5845),o:Sphingobacteriales(0.3031),f:Sphingobacteriaceae(0.1525),g:Parapedobacter(0.0143);
d:Bacteria(1.0000),p:Bacteroidetes(0.9802),c:Sphingobacteriia(0.6471),o:Sphingobacteriales(0.3394),f:Sphingobacteriaceae(0.1861),g:Pedobacter(0.0167);
d:Bacteria(1.0000),p:Bacteroidetes(0.9938),c:Bacteroidia(0.8562),o:Bacteroidales(0.5540),f:Porphyromonadaceae(0.1525),g:Paludibacter(0.0295);
d:Bacteria(1.0000),p:Bacteroidetes(0.9566),c:Sphingobacteriia(0.2725),o:Sphingobacteriales(0.1218),f:Sphingobacteriaceae(0.0454),g:Solitalea(0.0119);
d:Bacteria(1.0000),p:Firmicutes(0.9987),c:Clostridia(0.9954),o:Clostridiales(0.9954),f:Clostridiaceae_1(0.8778),g:Clostridium_sensu_stricto(0.7126);
d:Bacteria(1.0000),p:Firmicutes(0.6840),c:Clostridia(0.3342),o:Clostridiales(0.1579),f:Clostridiaceae_4(0.0296),g:Thermotalea(0.0094);
d:Bacteria(1.0000),p:Bacteroidetes(0.8620),c:Sphingobacteriia(0.2725),o:Sphingobacteriales(0.1218),f:Sphingobacteriaceae(0.0454),g:Pedobacter(0.0094);
d:Bacteria(1.0000),p:Bacteroidetes(0.9329),c:Sphingobacteriia(0.2725),o:Sphingobacteriales(0.1218),f:Sphingobacteriaceae(0.0454),g:Parapedobacter(0.0119);
d:Bacteria(1.0000),p:Firmicutes(0.8147),c:Clostridia(0.5219),o:Clostridiales(0.1579),f:Ruminococcaceae(0.0668),g:Intestinimonas(0.0119);
d:Bacteria(1.0000),p:Firmicutes(0.5785),c:Erysipelotrichia(0.2725),o:Erysipelotrichales(0.1218),f:Erysipelotrichaceae(0.0454),g:Clostridium_XVIII(0.0119);
d:Bacteria(0.9753),p:Bacteroidetes(0.5785),c:Sphingobacteriia(0.0315),o:Sphingobacteriales(0.0123),f:Sphingobacteriaceae(0.0042),g:Sphingobacterium(0.0034);
d:Bacteria(1.0000),p:Fibrobacteres(0.9991),c:Fibrobacteria(0.9704),o:Fibrobacterales(0.9597),f:Fibrobacteraceae(0.9109),g:Fibrobacter(0.8502);
d:Bacteria(1.0000),p:Bacteroidetes(0.9907),c:Sphingobacteriia(0.6471),o:Sphingobacteriales(0.3394),f:Sphingobacteriaceae(0.1861),g:Pedobacter(0.0167);
d:Bacteria(1.0000),p:Spirochaetes(0.8857),c:Spirochaetia(0.7097),o:Spirochaetales(0.3775),f:Spirochaetaceae(0.2263),g:Treponema(0.0094);
d:Bacteria(1.0000),p:Bacteroidetes(0.9093),c:Bacteroidia(0.3967),o:Bacteroidales(0.1942),f:Bacteroidaceae(0.0296),g:Bacteroides(0.0094);
d:Bacteria(1.0000),p:Firmicutes(0.8857),c:Clostridia(0.7097),o:Clostridiales(0.3031),f:Ruminococcaceae(0.1097),g:Intestinimonas(0.0119);
d:Bacteria(1.0000),p:Verrucomicrobia(0.8620),c:Subdivision5(0.6471),g:Subdivision5_genera_incertae_sedis(0.0409);
d:Bacteria(1.0000),p:Bacteroidetes(0.9899),c:Bacteroidia(0.5845),o:Bacteroidales(0.3031),f:Prevotellaceae(0.0883),g:Paraprevotella(0.0143);
d:Bacteria(1.0000),p:Bacteroidetes(0.9329),c:Sphingobacteriia(0.3967),o:Sphingobacteriales(0.1942),f:Sphingobacteriaceae(0.0883),g:Parapedobacter(0.0119);
d:Bacteria(1.0000),p:Spirochaetes(0.9993),c:Spirochaetia(0.9559),o:Spirochaetales(0.9037),f:Spirochaetaceae(0.8778),g:Treponema(0.5327);
d:Bacteria(1.0000),p:Firmicutes(0.9566),c:Clostridia(0.5845),o:Clostridiales(0.3031),f:Clostridiales_Incertae_Sedis_XIII(0.0883),g:Anaerovorax(0.0167);
d:Bacteria(1.0000),p:Bacteroidetes(0.9914),c:Bacteroidia(0.5845),o:Bacteroidales(0.3031),f:Porphyromonadaceae(0.1525),g:Paludibacter(0.0191);
d:Bacteria(1.0000),p:Firmicutes(0.8620),c:Clostridia(0.6471),o:Clostridiales(0.2305),f:Ruminococcaceae(0.1097),g:Clostridium_IV(0.0191);
d:Bacteria(1.0000),p:Bacteroidetes(0.9985),c:Bacteroidia(0.9631),o:Bacteroidales(0.9317),f:Prevotellaceae(0.6553),g:Prevotella(0.4804);
d:Bacteria(1.0000),p:Tenericutes(0.8147),c:Mollicutes(0.5219),o:Acholeplasmatales(0.2305),f:Acholeplasmataceae(0.1097),g:Acholeplasma(0.0191);
d:Bacteria(1.0000),p:Firmicutes(0.7674),c:Clostridia(0.2725),o:Clostridiales(0.1218),f:Lachnospiraceae(0.0454),g:Anaerostipes(0.0119);
d:Bacteria(1.0000),p:Bacteroidetes(0.9984),c:Bacteroidia(0.9937),o:Bacteroidales(0.9926),f:Prevotellaceae(0.6179),g:Prevotella(0.3760);
d:Bacteria(1.0000),p:Bacteroidetes(0.9999),c:Bacteroidia(0.9994),o:Bacteroidales(0.9994),f:Prevotellaceae(0.9935),g:Prevotella(0.9935);
d:Bacteria(1.0000),p:Bacteroidetes(0.9802),c:Sphingobacteriia(0.3342),o:Sphingobacteriales(0.1579),f:Sphingobacteriaceae(0.0668),g:Solitalea(0.0143);
d:Bacteria(1.0000),p:Bacteroidetes(0.8147),c:Bacteroidia(0.0345),o:Bacteroidales(0.0136),f:Rikenellaceae(0.0061),g:Alistipes(0.0043);
d:Bacteria(1.0000),p:Firmicutes(0.9946),c:Clostridia(0.8931),o:Clostridiales(0.7305),f:Ruminococcaceae(0.3872),g:Papillibacter(0.0523);
d:Bacteria(1.0000),p:Tenericutes(0.1875),c:Mollicutes(0.0345),o:Anaeroplasmatales(0.0136),f:Anaeroplasmataceae(0.0061),g:Asteroleplasma(0.0043);
d:Bacteria(1.0000),p:Bacteroidetes(0.8147),c:Sphingobacteriia(0.0315),o:Sphingobacteriales(0.0123),f:Sphingobacteriaceae(0.0042),g:Sphingobacterium(0.0034);
d:Bacteria(1.0000),p:Bacteroidetes(0.9093),c:Sphingobacteriia(0.2725),o:Sphingobacteriales(0.1218),f:Sphingobacteriaceae(0.0454),g:Pedobacter(0.0094);
d:Bacteria(1.0000),p:Bacteroidetes(0.9986),c:Bacteroidia(0.9931),o:Bacteroidales(0.9862),f:Prevotellaceae(0.7676),g:Prevotella(0.3238);
d:Bacteria(1.0000),p:Bacteroidetes(0.9566),c:Sphingobacteriia(0.3342),o:Sphingobacteriales(0.1579),f:Sphingobacteriaceae(0.0668),g:Pedobacter(0.0119);
d:Bacteria(1.0000),p:Firmicutes(0.9962),c:Clostridia(0.9299),o:Clostridiales(0.8337),f:Ruminococcaceae(0.5055),g:Clostridium_IV(0.0523);
d:Bacteria(1.0000),p:Firmicutes(0.9989),c:Clostridia(0.9777),o:Clostridiales(0.9693),f:Ruminococcaceae(0.8943),g:Anaerotruncus(0.5327);
d:Bacteria(0.9753),p:Bacteroidetes(0.7911),c:Bacteroidia(0.0285),o:Bacteroidales(0.0111),f:Porphyromonadaceae(0.0037),g:Dysgonomonas(0.0031);
d:Bacteria(1.0000),p:Bacteroidetes(0.9802),c:Sphingobacteriia(0.3342),o:Sphingobacteriales(0.1579),f:Sphingobacteriaceae(0.0668),g:Solitalea(0.0143);
d:Bacteria(1.0000),p:Firmicutes(0.9962),c:Clostridia(0.9023),o:Clostridiales(0.7746),f:Eubacteriaceae(0.3068),g:Eubacterium(0.0751);
d:Bacteria(1.0000),p:Firmicutes(0.9907),c:Clostridia(0.8470),o:Clostridiales(0.5099),f:Ruminococcaceae(0.2665),g:Pseudobacteroides(0.0215);
d:Bacteria(1.0000),p:Firmicutes(0.9987),c:Negativicutes(0.9741),o:Selenomonadales(0.9672),f:Acidaminococcaceae(0.8861),g:Phascolarctobacterium(0.3760);
d:Bacteria(1.0000),p:Firmicutes(0.9999),c:Clostridia(0.9947),o:Clostridiales(0.9947),f:Lachnospiraceae(0.9770),g:Roseburia(0.8502);
d:Bacteria(1.0000),p:Bacteroidetes(0.8620),c:Bacteroidia(0.4593),o:Bacteroidales(0.2305),f:Bacteroidaceae(0.0140),g:Bacteroides(0.0060);
d:Bacteria(1.0000),p:Firmicutes(0.9914),c:Clostridia(0.8562),o:Clostridiales(0.5540),f:Ruminococcaceae(0.3068),g:Pseudobacteroides(0.0295);
d:Bacteria(1.0000),p:Verrucomicrobia(0.9093),c:Subdivision5(0.7723),g:Subdivision5_genera_incertae_sedis(0.0637);
d:Bacteria(1.0000),p:Verrucomicrobia(0.7674),c:Subdivision5(0.3967),g:Subdivision5_genera_incertae_sedis(0.0167);

Table S2

PERMANOVA test results for effects on the GI microbiota using unweighted UniFrac distances

Factor	R-squared	P-value	Groupings
Individual	0.15	0.001	Monkey #1-39 (n=1-12; mean=8)
Reproductive unit	0.04	0.001	Unit #1-8 (n=8-84; mean=40)
Season	0.124	0.001	Dry (n=142), Wet (n=174)
Age	0.004	0.001	Prime (n=246), Old (n=70)
Fecundity status	0.041	0.001	T1 (n=27), T2 (n=13), T3 (n=32), PPA (n=178), Cycling (n=66)
Coenurosis		n.s.	Swelling (n=235), No swelling (n=81)

Table S3

PERMANOVA test results for effects on the GI microbiota using weighted UniFrac distances.

Factor	R-squared	P-value	Groupings
Individual	0.182	0.001	Monkey #1-39 (n=1-12; mean=8)
Reproductive unit	0.05	0.001	Unit #1-8 (n=8-84; mean=40)
Season	0.045	0.001	Dry (n=142), Wet (n=174)
Age		n.s.	Prime (n=246), Old (n=70)
Fecundity status	0.024	0.001	T1 (n=27), T2 (n=13), T3 (n=32), PPA (n=178), Cycling (n=66)
Coenurosis	0.008	0.001	Swelling (n=235), No swelling (n=81)

Table S4

OTUs with differential occurrence in the dry relative to the wet season (logFC=log fold change, FDR=false discovery rate)

Taxonomic assignment (probability of correct assignment in parentheses)	logFC	PValue	FDR
d:Bacteria(1.0000),p:Bacteroidetes(0.9992),c:Bacteroidia(0.8470),o:Bacteroidales(0.5099),f:Bacteroidaceae(0.1097),g:Bacteroides(0.0191);	2.858893	1.25E-28	2.02E-26
d:Bacteria(1.0000),p:Tenericutes(0.1875),c:Mollicutes(0.0345),o:Anaeroplasmatales(0.0136),f:Anaeroplasmataceae(0.0061),g:Asteroleplasma(0.0043);	3.358813	3.03E-27	2.46E-25
d:Bacteria(1.0000),p:Bacteroidetes(0.9566),c:Sphingobacteriia(0.3342),o:Sphingobacteriales(0.1579),f:Sphingobacteriaceae(0.0668),g:Pedobacter(0.0119);	2.19776	7.34E-26	3.96E-24
d:Bacteria(1.0000),p:Bacteroidetes(0.9899),c:Bacteroidia(0.5845),o:Bacteroidales(0.3031),f:Prevotellaceae(0.0883),g:Paraprevotella(0.0143);	2.275645	1.20E-24	4.85E-23
d:Bacteria(1.0000),p:Firmicutes(0.8620),c:Clostridia(0.4593),o:Clostridiales(0.2305),f:Lachnospiraceae(0.0454),g:Parasporobacterium(0.0119);	2.414641	1.12E-22	3.64E-21
d:Bacteria(1.0000),p:Tenericutes(0.1875),c:Mollicutes(0.0345),o:Anaeroplasmatales(0.0136),f:Anaeroplasmataceae(0.0061),g:Asteroleplasma(0.0043);	2.130666	3.70E-21	1.00E-19
d:Bacteria(0.9753),p:Bacteroidetes(0.7911),c:Bacteroidia(0.0285),o:Bacteroidales(0.0111),f:Porphyromonadaceae(0.0037),g:Dysgonomonas(0.0031);	2.316026	1.47E-20	3.41E-19
d:Bacteria(1.0000),p:Bacteroidetes(0.8620),c:Sphingobacteriia(0.2725),o:Sphingobacteriales(0.1218),f:Sphingobacteriaceae(0.0454),g:Pedobacter(0.0094);	1.769521	3.71E-20	7.52E-19
d:Bacteria(1.0000),p:Firmicutes(0.9938),c:Clostridia(0.8654),o:Clostridiales(0.5981),f:Lachnospiraceae(0.3068),g:Cellulosilyticum(0.0751);	1.998259	1.10E-19	1.99E-18
d:Bacteria(1.0000),p:Firmicutes(0.9991),c:Clostridia(0.9449),o:Clostridiales(0.8617),f:Eubacteriaceae(0.5804),g:Eubacterium(0.3760);	1.979935	2.33E-19	3.77E-18
d:Bacteria(1.0000),p:Bacteroidetes(0.9938),c:Bacteroidia(0.8562),o:Bacteroidales(0.5540),f:Porphyromonadaceae(0.1525),g:Paludibacter(0.0295);	1.897839	5.57E-19	8.20E-18
d:Bacteria(1.0000),p:Bacteroidetes(0.9802),c:Sphingobacteriia(0.6471),o:Sphingobacteriales(0.3394),f:Sphingobacteriaceae(0.1861),g:Pedobacter(0.0167);	1.435311	2.80E-18	3.78E-17
d:Bacteria(1.0000),p:Firmicutes(0.9914),c:Clostridia(0.8562),o:Clostridiales(0.5540),f:Ruminococcaceae(0.3068),g:Pseudobacteroides(0.0295);	1.311853	6.36E-17	7.92E-16
d:Bacteria(1.0000),p:Verrucomicrobia(0.7674),c:Subdivision5(0.3967),g:Subdivision5_genera_incertae_sedis(0.0167);	1.314828	7.07E-17	8.18E-16
d:Bacteria(1.0000),p:Firmicutes(0.9999),c:Clostridia(0.9937),o:Clostridiales(0.9926),f:Clostridiaceae_1(0.9701),g:Clostridium_sensu_stricto(0.9701);	1.957695	1.15E-15	1.25E-14
d:Bacteria(1.0000),p:Firmicutes(0.6840),c:Clostridia(0.1500),o:Clostridiales(0.0673),f:Lachnospiraceae(0.0061),g:Lachnospira(0.0043);	1.856679	2.17E-15	2.19E-14
d:Bacteria(1.0000),p:Firmicutes(0.9970),c:Clostridia(0.9207),o:Clostridiales(0.8197),f:Eubacteriaceae(0.4275),g:Eubacterium(0.1672);	1.376929	1.18E-14	1.12E-13
d:Bacteria(1.0000),p:Bacteroidetes(0.9802),c:Sphingobacteriia(0.3342),o:Sphingobacteriales(0.1579),f:Sphingobacteriaceae(0.0668),g:Solitalea(0.0143);	1.698392	1.39E-14	1.25E-13
d:Bacteria(1.0000),p:Firmicutes(0.9962),c:Clostridia(0.9023),o:Clostridiales(0.7746),f:Eubacteriaceae(0.3068),g:Eubacterium(0.0751);	1.374545	5.03E-14	4.28E-13
d:Bacteria(1.0000),p:Bacteroidetes(0.9093),c:Bacteroidia(0.3967),o:Bacteroidales(0.1942),f:Bacteroidaceae(0.0296),g:Bacteroides(0.0094);	1.816614	2.14E-13	1.73E-12
d:Bacteria(1.0000),p:Bacteroidetes(0.9329),c:Sphingobacteriia(0.5845),o:Sphingobacteriales(0.3031),f:Sphingobacteriaceae(0.1525),g:Pedobacter(0.0143);	1.723097	6.01E-13	4.63E-12
d:Bacteria(1.0000),p:Firmicutes(0.9946),c:Clostridia(0.8931),o:Clostridiales(0.7305),f:Ruminococcaceae(0.3872),g:Papillibacter(0.0523);	1.130419	7.14E-13	5.26E-12
d:Bacteria(1.0000),p:Firmicutes(0.9989),c:Clostridia(0.9777),o:Clostridiales(0.9693),f:Ruminococcaceae(0.8943),g:Anaerotruncus(0.5327);	1.225745	7.72E-13	5.44E-12
d:Bacteria(1.0000),p:Bacteroidetes(0.9802),c:Sphingobacteriia(0.4593),o:Sphingobacteriales(0.2305),f:Sphingobacteriaceae(0.1097),g:Pedobacter(0.0143);	-1.17194	9.10E-13	6.14E-12
d:Bacteria(1.0000),p:Firmicutes(0.9907),c:Clostridia(0.8470),o:Clostridiales(0.5099),f:Ruminococcaceae(0.2665),g:Pseudobacteroides(0.0215);	1.418156	2.31E-12	1.50E-11
d:Bacteria(1.0000),p:Bacteroidetes(0.9329),c:Sphingobacteriia(0.2725),o:Sphingobacteriales(0.1218),f:Sphingobacteriaceae(0.0454),g:Pedobacter(0.0094);	1.593407	2.44E-12	1.52E-11
d:Bacteria(1.0000),p:Verrucomicrobia(0.8620),c:Subdivision5(0.6471),g:Subdivision5_genera_incertae_sedis(0.0409);	1.782291	3.40E-12	1.97E-11
d:Bacteria(1.0000),p:Bacteroidetes(0.9986),c:Bacteroidia(0.9931),o:Bacteroidales(0.9862),f:Prevotellaceae(0.7676),g:Prevotella(0.3238);	1.516737	3.40E-12	1.97E-11
d:Bacteria(1.0000),p:Bacteroidetes(0.9984),c:Bacteroidia(0.9937),o:Bacteroidales(0.9926),f:Prevotellaceae(0.6179),g:Prevotella(0.3760);	1.567456	4.64E-12	2.59E-11
d:Bacteria(1.0000),p:Verrucomicrobia(0.8620),c:Subdivision5(0.6471),g:Subdivision5_genera_incertae_sedis(0.0409);	1.245278	2.53E-11	1.37E-10
d:Bacteria(1.0000),p:Firmicutes(0.9938),c:Clostridia(0.8838),o:Clostridiales(0.6864),f:Lachnospiraceae(0.3872),g:Cellulosilyticum(0.0751);	1.676121	2.18E-10	1.14E-09
d:Bacteria(1.0000),p:Bacteroidetes(0.9899),c:Bacteroidia(0.5845),o:Bacteroidales(0.3031),f:Porphyromonadaceae(0.1525),g:Barnesiella(0.0167);	1.442678	3.80E-10	1.92E-09
d:Bacteria(1.0000),p:Bacteroidetes(0.9566),c:Bacteroidia(0.2725),o:Bacteroidales(0.1218),f:Marinilabiliaceae(0.0296),g:Alkalitalea(0.0094);	1.493547	4.77E-10	2.34E-09
d:Bacteria(1.0000),p:Firmicutes(0.9946),c:Clostridia(0.9207),o:Clostridiales(0.7746),f:Ruminococcaceae(0.4677),g:Intestinimonas(0.0865);	1.802408	5.89E-10	2.81E-09
d:Bacteria(1.0000),p:Firmicutes(0.9962),c:Clostridia(0.9115),o:Clostridiales(0.8057),f:Ruminococcaceae(0.3872),g:Papillibacter(0.0751);	1.038515	8.86E-10	4.10E-09
d:Bacteria(1.0000),p:Bacteroidetes(0.9985),c:Bacteroidia(0.9631),o:Bacteroidales(0.9317),f:Prevotellaceae(0.6553),g:Prevotella(0.4804);	1.290259	1.46E-09	6.58E-09
d:Bacteria(1.0000),p:Bacteroidetes(0.9914),c:Bacteroidia(0.5845),o:Bacteroidales(0.3031),f:Porphyromonadaceae(0.1525),g:Paludibacter(0.0191);	1.354358	2.91E-09	1.28E-08
d:Bacteria(1.0000),p:Bacteroidetes(0.9802),c:Sphingobacteriia(0.3342),o:Sphingobacteriales(0.1579),f:Sphingobacteriaceae(0.0668),g:Solitalea(0.0143);	1.257513	3.39E-09	1.45E-08
d:Bacteria(1.0000),p:Fibrobacteres(0.9991),c:Fibrobacteria(0.9704),o:Fibrobacterales(0.9597),f:Fibrobacteraceae(0.9109),g:Fibrobacter(0.8502);	1.183649	4.86E-09	2.02E-08
d:Bacteria(1.0000),p:Firmicutes(0.9962),c:Clostridia(0.9207),o:Clostridiales(0.8197),f:Clostridiales_Incertae_Sedis_XIII(0.3470),g:Anaerovorax(0.0865);	1.177757	5.63E-09	2.28E-08
d:Bacteria(1.0000),p:Bacteroidetes(0.9930),c:Bacteroidia(0.8654),o:Bacteroidales(0.5981),f:Prevotellaceae(0.2263),g:Paraprevotella(0.0523);	1.440917	1.89E-08	7.45E-08
d:Bacteria(1.0000),p:Firmicutes(0.9978),c:Clostridia(0.9391),o:Clostridiales(0.8477),f:Ruminococcaceae(0.6179),g:Oscillibacter(0.0751);	0.949357	2.04E-08	7.88E-08
d:Bacteria(1.0000),p:Firmicutes(0.9997),c:Clostridia(0.9449),o:Clostridiales(0.8617),f:Lachnospiraceae(0.7676),g:Clostridium_XlVa(0.6095);	1.424894	2.39E-08	9.01E-08
d:Bacteria(1.0000),p:Firmicutes(0.9566),c:Clostridia(0.6471),o:Clostridiales(0.3394),f:Lachnospiraceae(0.1311),g:Clostridium_XlVa(0.0191);	1.276749	3.32E-08	1.22E-07
d:Bacteria(1.0000),p:Spirochaetes(0.9970),c:Spirochaetia(0.9449),o:Spirochaetales(0.8617),f:Spirochaetaceae(0.7676),g:Treponema(0.4282);	1.319918	3.48E-08	1.25E-07
d:Bacteria(1.0000),p:Bacteroidetes(0.9986),c:Bacteroidia(0.9960),o:Bacteroidales(0.9960),f:Prevotellaceae(0.8943),g:Prevotella(0.4804);	1.289395	7.10E-08	2.50E-07
d:Bacteria(1.0000),p:Firmicutes(0.9987),c:Negativicutes(0.9741),o:Selenomonadales(0.9672),f:Acidaminococcaceae(0.8861),g:Phascolarctobacterium(0.3760);	1.200097	9.25E-08	3.19E-07

d:Archaea(0.9753),p:Euryarchaeota(0.8384),c:Thermoplasmata(0.1500),o:Methanomassiliicoccales(0.0673),f:Methanomassiliicoccaceae(0.0218),g:Methanomassiliicoccus(0.0077);	1.312348	1.52E-07	5.14E-07
d:Bacteria(1.0000),p:Bacteroidetes(0.9994),c:Bacteroidia(0.9981),o:Bacteroidales(0.9981),f:Prevotellaceae(0.9837),g:Prevotella(0.9189);	1.456415	2.03E-07	6.70E-07
d:Bacteria(0.9753),p:Bacteroidetes(0.5785),c:Sphingobacteriia(0.0315),o:Sphingobacteriales(0.0123),f:Sphingobacteriaceae(0.0042),g:Sphingobacterium(0.0034);	1.23033	3.30E-07	1.07E-06
d:Bacteria(1.0000),p:Bacteroidetes(0.9907),c:Sphingobacteriia(0.6471),o:Sphingobacteriales(0.3394),f:Sphingobacteriaceae(0.1861),g:Pedobacter(0.0167);	1.102183	3.95E-07	1.26E-06
d:Bacteria(1.0000),p:Firmicutes(0.9938),c:Clostridia(0.9023),o:Clostridiales(0.7305),f:Ruminococcaceae(0.2665),g:Papillibacter(0.0637);	0.884941	4.49E-07	1.40E-06
d:Bacteria(1.0000),p:Firmicutes(0.1875),c:Erysipelotrichia(0.0315),o:Erysipelotrichales(0.0123),f:Erysipelotrichaceae(0.0042),g:Erysipelotrichaceae_incertae_sedis(0.0034);	1.352221	5.16E-07	1.58E-06
d:Bacteria(1.0000),p:Bacteroidetes(0.9907),c:Bacteroidia(0.5219),o:Bacteroidales(0.2668),f:Porphyromonadaceae(0.1311),g:Paludibacter(0.0215);	1.145265	5.89E-07	1.77E-06
d:Bacteria(1.0000),p:Bacteroidetes(0.9907),c:Sphingobacteriia(0.5845),o:Sphingobacteriales(0.3031),f:Sphingobacteriaceae(0.1525),g:Solitalea(0.0167);	0.965738	7.21E-07	2.12E-06
d:Bacteria(1.0000),p:Firmicutes(0.8147),c:Clostridia(0.3967),o:Clostridiales(0.1942),f:Lachnospiraceae(0.0454),g:Parasporobacterium(0.0119);	1.154856	1.48E-06	4.25E-06
d:Bacteria(1.0000),p:Firmicutes(0.9987),c:Clostridia(0.9954),o:Clostridiales(0.9954),f:Clostridiaceae_1(0.8778),g:Clostridium_sensu_stricto(0.7126);	0.92472	1.50E-06	4.25E-06
d:Bacteria(1.0000),p:Verrucomicrobia(0.8857),c:Subdivision5(0.7097),g:Subdivision5_genera_incertae_sedis(0.0523);	1.194926	2.00E-06	5.58E-06
d:Bacteria(1.0000),p:Bacteroidetes(0.9329),c:Sphingobacteriia(0.3967),o:Sphingobacteriales(0.1942),f:Sphingobacteriaceae(0.0883),g:Parapedobacter(0.0119);	0.833392	3.69E-06	1.01E-05
d:Archaea(1.0000),p:Euryarchaeota(0.9938),c:Thermoplasmata(0.8654),o:Methanomassiliicoccales(0.4658),f:Methanomassiliicoccaceae(0.3068),g:Methanomassiliicoccus(0.0751);	1.012375	3.91E-06	1.05E-05
d:Bacteria(1.0000),p:Firmicutes(0.5785),c:Erysipelotrichia(0.2725),o:Erysipelotrichales(0.1218),f:Erysipelotrichaceae(0.0454),g:Clostridium_XVIII(0.0119);	1.060438	5.07E-06	1.35E-05
d:Bacteria(1.0000),p:Firmicutes(0.9093),c:Clostridia(0.7723),o:Clostridiales(0.3394),f:Ruminococcaceae(0.1311),g:Intestinimonas(0.0215);	-0.82871	5.58E-06	1.46E-05
d:Bacteria(1.0000),p:Bacteroidetes(0.8147),c:Sphingobacteriia(0.0345),o:Sphingobacteriales(0.0136),f:Sphingobacteriaceae(0.0061),g:Olivibacter(0.0043);	1.147745	6.10E-06	1.57E-05
d:Bacteria(1.0000),p:Firmicutes(0.7674),c:Negativicutes(0.2725),o:Selenomonadales(0.1218),f:Veillonellaceae(0.0454),g:Selenomonas(0.0119);	0.909471	8.62E-06	2.18E-05
d:Bacteria(1.0000),p:Firmicutes(0.9991),c:Clostridia(0.9975),o:Clostridiales(0.9975),f:Ruminococcaceae(0.9973),g:Faecalibacterium(0.9829);	1.280267	1.01E-05	2.51E-05
d:Bacteria(1.0000),p:Bacteroidetes(0.8620),c:Bacteroidia(0.4593),o:Bacteroidales(0.2305),f:Bacteroidaceae(0.0140),g:Bacteroides(0.0060);	0.878103	1.18E-05	2.90E-05
d:Bacteria(1.0000),p:Spirochaetes(0.9990),c:Spirochaetia(0.9777),o:Spirochaetales(0.9693),f:Spirochaetaceae(0.9274),g:Treponema(0.7126);	1.06847	1.23E-05	2.98E-05
d:Bacteria(1.0000),p:Firmicutes(0.7674),c:Clostridia(0.2725),o:Clostridiales(0.1218),f:Clostridiaceae_1(0.0296),g:Clostridium_sensu_stricto(0.0094);	0.829075	1.80E-05	4.28E-05
d:Bacteria(1.0000),p:Firmicutes(0.9970),c:Clostridia(0.9391),o:Clostridiales(0.8477),f:Ruminococcaceae(0.5804),g:Ruminococcus(0.2716);	0.916605	2.42E-05	5.67E-05
d:Bacteria(1.0000),p:Verrucomicrobia(0.9093),c:Subdivision5(0.7723),g:Subdivision5_genera_incertae_sedis(0.0637);	0.69256	2.59E-05	5.99E-05
d:Bacteria(1.0000),p:Bacteroidetes(0.8147),c:Sphingobacteriia(0.0315),o:Sphingobacteriales(0.0123),f:Sphingobacteriaceae(0.0042),g:Sphingobacterium(0.0034);	1.021437	4.61E-05	0.000105
d:Bacteria(1.0000),p:Firmicutes(0.9566),c:Clostridia(0.8470),o:Clostridiales(0.4658),f:Ruminococcaceae(0.1861),g:Clostridium_IV(0.0167);	-0.79628	5.24E-05	0.000118
d:Bacteria(1.0000),p:Firmicutes(0.9989),c:Clostridia(0.9968),o:Clostridiales(0.9968),f:Peptostreptococcaceae(0.9743),g:Clostridium_XI(0.9743);	0.959594	5.76E-05	0.000128
d:Bacteria(1.0000),p:Proteobacteria(0.9914),c:Deltaproteobacteria(0.8562),o:Myxococcales(0.5540),f:Myxococcaceae(0.1311),g:Corallococcus(0.0215);	0.958855	8.59E-05	0.000188
d:Bacteria(1.0000),p:Firmicutes(0.5785),c:Clostridia(0.2725),o:Clostridiales(0.1218),f:Clostridiaceae_4(0.0218),g:Thermotalea(0.0077);	0.762844	0.000115	0.000247
d:Bacteria(1.0000),p:Tenericutes(0.9930),c:Mollicutes(0.9023),o:Anaeroplasmatales(0.5981),f:Anaeroplasmataceae(0.4275),g:Anaeroplasma(0.1672);	0.837873	0.000121	0.000259
d:Bacteria(1.0000),p:Firmicutes(0.9988),c:Clostridia(0.9631),o:Clostridiales(0.9317),f:Lachnospiraceae(0.6553),g:Ruminococcus2(0.3238);	1.092898	0.000128	0.000269
d:Bacteria(1.0000),p:Bacteroidetes(0.9988),c:Bacteroidia(0.9023),o:Bacteroidales(0.7746),f:Prevotellaceae(0.2665),g:Prevotella(0.0637);	1.047528	0.00015	0.000312
d:Bacteria(1.0000),p:Bacteroidetes(0.9566),c:Sphingobacteriia(0.2725),o:Sphingobacteriales(0.1218),f:Sphingobacteriaceae(0.0454),g:Solitalea(0.0119);	0.787451	0.000154	0.000315
d:Bacteria(1.0000),p:Firmicutes(0.7674),c:Erysipelotrichia(0.3967),o:Erysipelotrichales(0.1942),f:Erysipelotrichaceae(0.0883),g:Clostridium_XVIII(0.0167);	1.044038	0.000162	0.000329
d:Bacteria(1.0000),p:Verrucomicrobia(0.9938),c:Subdivision5(0.9115),g:Subdivision5_genera_incertae_sedis(0.4282);	0.815127	0.000178	0.000356
d:Bacteria(1.0000),p:Tenericutes(0.4731),c:Mollicutes(0.2113),o:Acholeplasmatales(0.0945),f:Acholeplasmataceae(0.0296),g:Acholeplasma(0.0094);	0.920854	0.000184	0.000364
d:Bacteria(1.0000),p:Firmicutes(0.9986),c:Clostridia(0.9947),o:Clostridiales(0.9947),f:Lachnospiraceae(0.9687),g:Pseudobutyrivibrio(0.8158);	0.855982	0.000197	0.000384
d:Bacteria(1.0000),p:Firmicutes(0.5785),c:Clostridia(0.2725),o:Clostridiales(0.1218),f:Clostridiaceae_1(0.0218),g:Clostridium_sensu_stricto(0.0077);	0.882373	0.000216	0.000417
d:Bacteria(1.0000),p:Bacteroidetes(0.9093),c:Bacteroidia(0.3342),o:Bacteroidales(0.1579),f:Porphyromonadaceae(0.0668),g:Paludibacter(0.0119);	0.988447	0.000349	0.000665
d:Bacteria(1.0000),p:Verrucomicrobia(0.2622),c:Subdivision5(0.0888),g:Subdivision5_genera_incertae_sedis(0.0060);	-0.66282	0.000398	0.000751
d:Bacteria(1.0000),p:Verrucomicrobia(0.8384),c:Subdivision5(0.5845),g:Subdivision5_genera_incertae_sedis(0.0295);	-0.76128	0.000495	0.000922
d:Bacteria(1.0000),p:Bacteroidetes(0.9899),c:Bacteroidia(0.7723),o:Bacteroidales(0.4216),f:Prevotellaceae(0.0883),g:Paraprevotella(0.0143);	0.935949	0.000576	0.00106
d:Bacteria(1.0000),p:Firmicutes(0.9938),c:Clostridia(0.8746),o:Clostridiales(0.6422),f:Ruminococcaceae(0.4275),g:Sporobacter(0.0523);	0.682704	0.000819	0.001492
d:Bacteria(1.0000),p:Bacteroidetes(0.9329),c:Sphingobacteriia(0.2725),o:Sphingobacteriales(0.1218),f:Sphingobacteriaceae(0.0454),g:Parapedobacter(0.0119);	0.638022	0.000929	0.001672
d:Bacteria(1.0000),p:Firmicutes(0.9962),c:Clostridia(0.9299),o:Clostridiales(0.8337),f:Ruminococcaceae(0.5055),g:Clostridium_IV(0.0523);	0.51054	0.000948	0.001688
d:Bacteria(1.0000),p:Spirochaetes(0.9991),c:Spirochaetia(0.9668),o:Spirochaetales(0.9457),f:Spirochaetaceae(0.9026),g:Treponema(0.7814);	-1.0076	0.000991	0.001745
d:Bacteria(1.0000),p:Firmicutes(0.7674),c:Clostridia(0.3967),o:Clostridiales(0.1942),f:Clostridiaceae_1(0.0668),g:Clostridium_sensu_stricto(0.0143);	0.841694	0.001094	0.001906
d:Bacteria(1.0000),p:Firmicutes(0.2622),c:Erysipelotrichia(0.0345),o:Erysipelotrichales(0.0136),f:Erysipelotrichaceae(0.0061),g:Clostridium_XVIII(0.0043);	0.739456	0.0022	0.003792
d:Bacteria(0.9753),p:Bacteroidetes(0.7674),c:Sphingobacteriia(0.0345),o:Sphingobacteriales(0.0136),f:Sphingobacteriaceae(0.0061),g:Mucilaginibacter(0.0031);	0.750038	0.002554	0.004354
d:Archaea(1.0000),p:Euryarchaeota(0.9930),c:Thermoplasmata(0.8562),o:Methanomassiliicoccales(0.4216),f:Methanomassiliicoccaceae(0.2665),g:Methanomassiliicoccus(0.0637);	0.692281	0.002943	0.004967
d:Bacteria(1.0000),p:Firmicutes(0.8620),c:Clostridia(0.3967),o:Clostridiales(0.1942),f:Clostridiaceae_4(0.0454),g:Thermotalea(0.0119);	0.612018	0.003087	0.005156

d:Bacteria(1.0000),p:Firmicutes(0.9987),c:Clostridia(0.9924),o:Clostridiales(0.9799),f:Ruminococcaceae(0.6553),g:Butyricicoccus(0.4804);	0.662184	0.003749	0.006197
d:Bacteria(1.0000),p:Verrucomicrobia(0.8620),c:Subdivision5(0.6471),g:Subdivision5_genera_incertae_sedis(0.0409);	0.614024	0.00393	0.006378
d:Bacteria(1.0000),p:Firmicutes(0.7674),c:Clostridia(0.3967),o:Clostridiales(0.1942),f:Syntrophomonadaceae(0.0883),g:Syntrophothermus(0.0167);	0.687868	0.003937	0.006378
d:Bacteria(1.0000),p:Firmicutes(0.9999),c:Clostridia(0.9947),o:Clostridiales(0.9947),f:Lachnospiraceae(0.9770),g:Roseburia(0.8502);	0.578093	0.004589	0.00736
d:Bacteria(1.0000),p:Firmicutes(0.8147),c:Clostridia(0.5219),o:Clostridiales(0.2305),f:Ruminococcaceae(0.0454),g:Ruminococcus(0.0094);	-0.63447	0.005076	0.008061
d:Bacteria(1.0000),p:Bacteroidetes(0.9802),c:Sphingobacteriia(0.5845),o:Sphingobacteriales(0.3031),f:Sphingobacteriaceae(0.1525),g:Parapedobacter(0.0143);	0.603541	0.005808	0.009135

Table S5

Putative taxonomy of OTUs that were found in only in geladas and no other primates (assignment probabilities in parentheses)

d:Archaea(1.0000),p:Euryarchaeota(0.9930),c:Thermoplasmata(0.8562),o:Methanomassiliicoccales(0.4216),f:Methanomassiliicoccaceae(0.2665),g:Methanomassiliicoccus(0.0637);
d:Bacteria(1.0000),p:Spirochaetes(0.9991),c:Spirochaetia(0.9668),o:Spirochaetales(0.9457),f:Spirochaetaceae(0.9026),g:Treponema(0.7814);
d:Bacteria(1.0000),p:Bacteroidetes(0.9329),c:Sphingobacteriia(0.2725),o:Sphingobacteriales(0.1218),f:Sphingobacteriaceae(0.0454),g:Pedobacter(0.0094);
d:Bacteria(1.0000),p:Firmicutes(0.1875),c:Erysipelotrichia(0.0315),o:Erysipelotrichales(0.0123),f:Erysipelotrichaceae(0.0042),g:Erysipelotrichaceae_incertae_sedis(0.0034);
d:Bacteria(1.0000),p:Verrucomicrobia(0.9093),c:Subdivision5(0.7723),g:Subdivision5_genera_incertae_sedis(0.0637);
d:Bacteria(1.0000),p:Firmicutes(0.5785),c:Clostridia(0.2725),o:Clostridiales(0.1218),f:Clostridiaceae_4(0.0218),g:Thermotalea(0.0077);
d:Bacteria(1.0000),p:Bacteroidetes(0.9899),c:Bacteroidia(0.5845),o:Bacteroidales(0.3031),f:Porphyromonadaceae(0.1525),g:Barnesiella(0.0167);
d:Bacteria(1.0000),p:Bacteroidetes(0.9992),c:Bacteroidia(0.8470),o:Bacteroidales(0.5099),f:Bacteroidaceae(0.1097),g:Bacteroides(0.0191);
d:Bacteria(1.0000),p:Verrucomicrobia(0.9938),c:Subdivision5(0.9115),g:Subdivision5_genera_incertae_sedis(0.4282);
d:Bacteria(1.0000),p:Verrucomicrobia(0.8857),c:Subdivision5(0.7097),g:Subdivision5_genera_incertae_sedis(0.0523);
d:Bacteria(1.0000),p:Verrucomicrobia(0.8620),c:Subdivision5(0.6471),g:Subdivision5_genera_incertae_sedis(0.0409);
d:Bacteria(1.0000),p:Firmicutes(0.9566),c:Clostridia(0.6471),o:Clostridiales(0.3394),f:Lachnospiraceae(0.1311),g:Clostridium_XlVa(0.0191);
d:Bacteria(1.0000),p:Firmicutes(0.9946),c:Clostridia(0.9207),o:Clostridiales(0.7746),f:Ruminococcaceae(0.4677),g:Intestinimonas(0.0865);
d:Bacteria(1.0000),p:Verrucomicrobia(0.8857),c:Subdivision5(0.7097),g:Subdivision5_genera_incertae_sedis(0.0523);
d:Bacteria(1.0000),p:Bacteroidetes(0.8620),c:Sphingobacteriia(0.2113),o:Sphingobacteriales(0.0945),f:Sphingobacteriaceae(0.0296),g:Parapedobacter(0.0077);
d:Bacteria(1.0000),p:Firmicutes(0.5785),c:Clostridia(0.2725),o:Clostridiales(0.1218),f:Clostridiaceae_1(0.0218),g:Clostridium_sensu_stricto(0.0077);
d:Bacteria(1.0000),p:Bacteroidetes(0.9566),c:Bacteroidia(0.2725),o:Bacteroidales(0.1218),f:Marinilabiliaceae(0.0296),g:Alkalitalea(0.0094);
d:Bacteria(1.0000),p:Verrucomicrobia(0.2622),c:Subdivision5(0.0888),g:Subdivision5_genera_incertae_sedis(0.0060);
d:Bacteria(1.0000),p:Firmicutes(0.9907),c:Erysipelotrichia(0.8746),o:Erysipelotrichales(0.6422),f:Erysipelotrichaceae(0.4677),g:Clostridium_XVIII(0.0409);
d:Bacteria(1.0000),p:Firmicutes(0.9978),c:Clostridia(0.9023),o:Clostridiales(0.7746),f:Lachnospiraceae(0.3872),g:Pseudobutyrivibrio(0.0637);
d:Bacteria(1.0000),p:Tenericutes(0.4731),c:Mollicutes(0.2113),o:Anaeroplasmatales(0.0945),f:Anaeroplasmataceae(0.0296),g:Asteroleplasma(0.0094);
d:Bacteria(1.0000),p:Fibrobacteres(0.9988),c:Fibrobacteria(0.9972),o:Fibrobacterales(0.9972),f:Fibrobacteraceae(0.9972),g:Fibrobacter(0.9972);
d:Bacteria(1.0000),p:Tenericutes(0.2622),c:Mollicutes(0.0888),o:Anaeroplasmatales(0.0400),f:Anaeroplasmataceae(0.0140),g:Asteroleplasma(0.0060);
d:Bacteria(1.0000),p:Firmicutes(0.8620),c:Erysipelotrichia(0.6471),o:Erysipelotrichales(0.3394),f:Erysipelotrichaceae(0.1861),g:Holdemania(0.0295);
d:Bacteria(1.0000),p:Firmicutes(0.9987),c:Clostridia(0.9704),o:Clostridiales(0.9597),f:Lachnospiraceae(0.8861),g:Pseudobutyrivibrio(0.4282);
d:Bacteria(1.0000),p:Bacteroidetes(0.9985),c:Bacteroidia(0.9935),o:Bacteroidales(0.9904),f:Prevotellaceae(0.7676),g:Prevotella(0.2194);
d:Bacteria(1.0000),p:Lentisphaerae(0.8384),c:Oligosphaeria(0.5845),o:Oligosphaerales(0.3031),f:Oligosphaeraceae(0.1525),g:Oligosphaera(0.0295);
d:Bacteria(1.0000),p:Bacteroidetes(0.9802),c:Sphingobacteriia(0.3967),o:Sphingobacteriales(0.1942),f:Sphingobacteriaceae(0.0883),g:Pedobacter(0.0143);
d:Bacteria(1.0000),p:Fibrobacteres(0.9922),c:Fibrobacteria(0.8931),o:Fibrobacterales(0.7305),f:Fibrobacteraceae(0.5430),g:Fibrobacter(0.3238);
d:Bacteria(1.0000),p:Firmicutes(0.9922),c:Clostridia(0.8562),o:Clostridiales(0.5540),f:Clostridiaceae_1(0.3470),g:Oxobacter(0.0523);
d:Bacteria(1.0000),p:Firmicutes(0.9989),c:Clostridia(0.9391),o:Clostridiales(0.8477),f:Lachnospiraceae(0.6553),g:Clostridium_XlVa(0.4282);
d:Bacteria(1.0000),p:Verrucomicrobia(0.9914),c:Subdivision5(0.8838),g:Subdivision5_genera_incertae_sedis(0.2716);
d:Bacteria(1.0000),p:Firmicutes(0.9992),c:Clostridia(0.9850),o:Clostridiales(0.9736),f:Lachnospiraceae(0.8051),g:Blautia(0.4282);
d:Bacteria(1.0000),p:Actinobacteria(0.9985),c:Actinobacteria(0.9595),o:Coriobacteriales(0.9177),f:Coriobacteriaceae(0.8861),g:Adlercreutzia(0.1150);
d:Bacteria(1.0000),p:Firmicutes(0.9938),c:Clostridia(0.8746),o:Clostridiales(0.6422),f:Lachnospiraceae(0.2665),g:Lachnospiracea_incertae_sedis(0.0295);
d:Bacteria(1.0000),p:Firmicutes(0.4731),c:Negativicutes(0.0345),o:Selenomonadales(0.0136),f:Veillonellaceae(0.0061),g:Anaerovibrio(0.0034);
d:Bacteria(1.0000),p:Firmicutes(0.9938),c:Clostridia(0.8931),o:Clostridiales(0.7305),f:Clostridiales_Incertae_Sedis_XIII(0.2263),g:Anaerovorax(0.0523);
d:Bacteria(1.0000),p:Bacteroidetes(0.9899),c:Bacteroidia(0.7723),o:Bacteroidales(0.4216),f:Porphyromonadaceae(0.2263),g:Barnesiella(0.0191);
d:Bacteria(1.0000),p:Firmicutes(0.7911),c:Clostridia(0.2725),o:Clostridiales(0.1218),f:Lachnospiraceae(0.0218),g:Lachnospiracea_incertae_sedis(0.0077);
d:Bacteria(1.0000),p:Firmicutes(0.9914),c:Clostridia(0.8562),o:Clostridiales(0.5540),f:Lachnospiraceae(0.2665),g:Cellulosilyticum(0.0409);
d:Bacteria(1.0000),p:Firmicutes(0.2622),c:Bacilli(0.0315),o:Lactobacillales(0.0123),f:Leuconostocaceae(0.0042),g:Fructobacillus(0.0034);
d:Bacteria(1.0000),p:Firmicutes(0.8620),c:Erysipelotrichia(0.5845),o:Erysipelotrichales(0.3031),f:Erysipelotrichaceae(0.1525),g:Erysipelotrichaceae_incertae_sedis(0.0295);
d:Bacteria(1.0000),p:Firmicutes(0.4731),c:Clostridia(0.1500),o:Clostridiales(0.0673),f:Lachnospiraceae(0.0061),g:Clostridium_XlVa(0.0043);
d:Bacteria(1.0000),p:Firmicutes(0.6840),c:Erysipelotrichia(0.3342),o:Erysipelotrichales(0.1579),f:Erysipelotrichaceae(0.0668),g:Bulleidia(0.0143);
d:Bacteria(1.0000),p:Tenericutes(0.8620),c:Mollicutes(0.6471),o:Acholeplasmatales(0.3031),f:Acholeplasmataceae(0.1525),g:Acholeplasma(0.0295);
d:Bacteria(1.0000),p:Proteobacteria(0.8620),c:Deltaproteobacteria(0.6471),o:Bdellovibrionales(0.3394),f:Bdellovibrionaceae(0.1861),g:Vampirovibrio(0.0409);
d:Bacteria(1.0000),p:Tenericutes(0.7911),c:Mollicutes(0.4593),o:Acholeplasmatales(0.2305),f:Acholeplasmataceae(0.1097),g:Acholeplasma(0.0191);
d:Bacteria(1.0000),p:Lentisphaerae(0.9907),c:Oligosphaeria(0.8746),o:Oligosphaerales(0.6422),f:Oligosphaeraceae(0.4677),g:Oligosphaera(0.2194);

d:Bacteria(1.0000),p:Bacteroidetes(0.9093),c:Sphingobacteriia(0.2113),o:Sphingobacteriales(0.0945),f:Sphingobacteriaceae(0.0296),g:Olivibacter(0.0094);
d:Bacteria(1.0000),p:Firmicutes(0.9329),c:Erysipelotrichia(0.8348),o:Erysipelotrichales(0.4658),f:Erysipelotrichaceae(0.3068),g:Holdemania(0.0523);
d:Bacteria(1.0000),p:Verrucomicrobia(0.8147),c:Subdivision5(0.5219),g:Subdivision5_genera_incertae_sedis(0.0215);
d:Bacteria(1.0000),p:Bacteroidetes(0.9093),c:Sphingobacteriia(0.2113),o:Sphingobacteriales(0.0945),f:Sphingobacteriaceae(0.0296),g:Solitalea(0.0094);
d:Bacteria(1.0000),p:Proteobacteria(0.7674),c:Alphaproteobacteria(0.3967),o:Rhodospirillales(0.0673),f:Rhodospirillaceae(0.0218),g:Azospirillum(0.0077);
d:Bacteria(1.0000),p:Firmicutes(0.5785),c:Erysipelotrichia(0.2725),o:Erysipelotrichales(0.1218),f:Erysipelotrichaceae(0.0454),g:Holdemania(0.0119);
d:Bacteria(1.0000),p:Tenericutes(0.2622),c:Mollicutes(0.0888),o:Acholeplasmatales(0.0400),f:Acholeplasmataceae(0.0140),g:Acholeplasma(0.0060);
d:Bacteria(1.0000),p:Firmicutes(0.7674),c:Clostridia(0.3967),o:Clostridiales(0.1942),f:Ruminococcaceae(0.0668),g:Pseudoflavonifractor(0.0119);
d:Bacteria(1.0000),p:Firmicutes(0.9566),c:Erysipelotrichia(0.8470),o:Erysipelotrichales(0.5099),f:Erysipelotrichaceae(0.3470),g:Bulleidia(0.0865);
d:Bacteria(1.0000),p:Firmicutes(0.4731),c:Clostridia(0.2113),o:Clostridiales(0.0945),f:Ruminococcaceae(0.0140),g:Clostridium_III(0.0060);
d:Bacteria(1.0000),p:Bacteroidetes(0.9938),c:Bacteroidia(0.8746),o:Bacteroidales(0.6422),f:Porphyromonadaceae(0.4677),g:Barnesiella(0.0215);
d:Bacteria(1.0000),p:Actinobacteria(0.9093),c:Actinobacteria(0.7723),o:Coriobacteriales(0.4216),f:Coriobacteriaceae(0.2665),g:Adlercreutzia(0.0143);
d:Bacteria(1.0000),p:Firmicutes(0.9899),c:Clostridia(0.7723),o:Clostridiales(0.4216),f:Incertae_Sedis_XI(0.1861),g:Clostridium_XII(0.0409);
d:Bacteria(1.0000),p:Firmicutes(0.9994),c:Clostridia(0.9741),o:Clostridiales(0.9672),f:Lachnospiraceae(0.8051),g:Clostridium_XlVa(0.6438);
d:Bacteria(1.0000),p:Firmicutes(0.8384),c:Clostridia(0.5845),o:Clostridiales(0.3031),f:Ruminococcaceae(0.0668),g:Ruminococcus(0.0143);
d:Bacteria(1.0000),p:Firmicutes(0.9991),c:Clostridia(0.9559),o:Clostridiales(0.9037),f:Ruminococcaceae(0.7676),g:Clostridium_IV(0.3760);
d:Bacteria(1.0000),p:Firmicutes(0.9989),c:Clostridia(0.9814),o:Clostridiales(0.9715),f:Ruminococcaceae(0.9026),g:Ruminococcus(0.7470);
d:Bacteria(1.0000),p:Firmicutes(0.9990),c:Clostridia(0.9449),o:Clostridiales(0.8617),f:Lachnospiraceae(0.5430),g:Anaerostipes(0.2194);
d:Bacteria(1.0000),p:Proteobacteria(0.2622),c:Gammaproteobacteria(0.0315),o:Oceanospirillales(0.0123),f:Alcanivoracaceae(0.0042),g:Alcanivorax(0.0034);
d:Bacteria(1.0000),p:Firmicutes(0.3677),c:Erysipelotrichia(0.0888),o:Erysipelotrichales(0.0400),f:Erysipelotrichaceae(0.0140),g:Clostridium_XVIII(0.0060);
d:Bacteria(1.0000),p:Bacteroidetes(0.9988),c:Bacteroidia(0.9741),o:Bacteroidales(0.9672),f:Porphyromonadaceae(0.9109),g:Parabacteroides(0.6095);
d:Bacteria(1.0000),p:Firmicutes(0.9566),c:Clostridia(0.8348),o:Clostridiales(0.4658),f:Clostridiales_Incertae_Sedis_XIII(0.2263),g:Mogibacterium(0.0523);
d:Bacteria(1.0000),p:Firmicutes(0.4731),c:Bacilli(0.2113),o:Bacillales(0.0945),f:Staphylococcaceae(0.0296),g:Jeotgalicoccus(0.0077);
d:Bacteria(1.0000),p:Verrucomicrobia(0.9566),c:Subdivision5(0.8470),g:Subdivision5_genera_incertae_sedis(0.0865);
d:Bacteria(1.0000),p:Firmicutes(0.9093),c:Clostridia(0.4593),o:Clostridiales(0.2305),f:Clostridiaceae_1(0.0668),g:Clostridium_sensu_stricto(0.0143);
d:Bacteria(1.0000),p:Firmicutes(0.6840),c:Clostridia(0.3342),o:Clostridiales(0.1579),f:Peptostreptococcaceae(0.0296),g:Clostridium_XI(0.0060);
d:Bacteria(1.0000),p:Proteobacteria(0.9946),c:Deltaproteobacteria(0.8838),o:Desulfovibrionales(0.6864),f:Desulfovibrionaceae(0.2665),g:Desulfovibrio(0.0523);
d:Bacteria(1.0000),p:Firmicutes(0.2622),c:Clostridia(0.0888),o:Clostridiales(0.0400),g:Natranaerovirga(0.0043);
d:Bacteria(1.0000),p:Firmicutes(0.9991),c:Erysipelotrichia(0.9668),o:Erysipelotrichales(0.9457),f:Erysipelotrichaceae(0.9026),g:Erysipelotrichaceae_incertae_sedis(0.6782);
d:Bacteria(1.0000),p:Firmicutes(0.9922),c:Erysipelotrichia(0.8838),o:Erysipelotrichales(0.6864),f:Erysipelotrichaceae(0.5055),g:Clostridium_XVIII(0.0523);
d:Bacteria(1.0000),p:Firmicutes(0.9992),c:Clostridia(0.9522),o:Clostridiales(0.8897),f:Lachnospiraceae(0.6553),g:Lachnospiracea_incertae_sedis(0.1150);
d:Bacteria(1.0000),p:Firmicutes(0.9914),c:Clostridia(0.8838),o:Clostridiales(0.6864),f:Ruminococcaceae(0.4677),g:Ruminococcus(0.0865);
d:Bacteria(1.0000),p:Firmicutes(0.9329),c:Clostridia(0.7723),o:Clostridiales(0.4216),f:Eubacteriaceae(0.1097),g:Eubacterium(0.0191);
d:Bacteria(1.0000),p:Firmicutes(0.9914),c:Clostridia(0.8746),o:Clostridiales(0.6422),f:Clostridiales_Incertae_Sedis_XIII(0.2263),g:Anaerovorax(0.0523);
d:Bacteria(1.0000),p:Firmicutes(0.7674),c:Bacilli(0.1500),o:Lactobacillales(0.0673),f:Lactobacillaceae(0.0218),g:Lactobacillus(0.0077);
d:Bacteria(1.0000),p:Firmicutes(0.8620),c:Erysipelotrichia(0.6471),o:Erysipelotrichales(0.3394),f:Erysipelotrichaceae(0.1861),g:Erysipelothrix(0.0295);
d:Bacteria(1.0000),p:Firmicutes(0.9993),c:Clostridia(0.9741),o:Clostridiales(0.9672),f:Lachnospiraceae(0.8051),g:Clostridium_XlVa(0.6438);
d:Bacteria(1.0000),p:Proteobacteria(0.8147),c:Alphaproteobacteria(0.4593),o:Rhodospirillales(0.0945),f:Rhodospirillaceae(0.0296),g:Fodinicurvata(0.0077);
d:Bacteria(1.0000),p:Firmicutes(0.2622),c:Clostridia(0.0345),o:Clostridiales(0.0136),f:Eubacteriaceae(0.0042),g:Eubacterium(0.0034);
d:Bacteria(1.0000),p:Firmicutes(0.1875),c:Clostridia(0.0345),o:Clostridiales(0.0136),f:Clostridiaceae_3(0.0061),g:Brassicibacter(0.0043);
d:Bacteria(1.0000),p:Firmicutes(0.7911),c:Clostridia(0.2725),o:Clostridiales(0.1218),f:Lachnospiraceae(0.0218),g:Clostridium_XlVa(0.0077);
d:Bacteria(1.0000),p:Firmicutes(0.9987),c:Clostridia(0.9522),o:Clostridiales(0.8897),f:Lachnospiraceae(0.5430),g:Clostridium_XlVa(0.2194);
d:Bacteria(1.0000),p:Firmicutes(0.8620),c:Clostridia(0.4593),o:Clostridiales(0.2305),f:Ruminococcaceae(0.0883),g:Clostridium_III(0.0167);
d:Bacteria(1.0000),p:Tenericutes(0.3677),c:Mollicutes(0.1500),o:Entomoplasmatales(0.0673),f:Entomoplasmataceae(0.0140),g:Mesoplasma(0.0060);
d:Bacteria(1.0000),p:Bacteroidetes(0.9988),c:Bacteroidia(0.9115),o:Bacteroidales(0.8057),f:Prevotellaceae(0.3872),g:Prevotella(0.0865);
d:Bacteria(1.0000),p:Proteobacteria(0.7674),c:Alphaproteobacteria(0.3967),o:Rhodospirillales(0.0945),f:Rhodospirillaceae(0.0296),g:Fodinicurvata(0.0060);
d:Bacteria(1.0000),p:Firmicutes(0.9802),c:Clostridia(0.8562),o:Clostridiales(0.5540),f:Ruminococcaceae(0.2263),g:Clostridium_IV(0.0191);
d:Bacteria(1.0000),p:Bacteroidetes(0.9566),c:Flavobacteriia(0.2725),o:Flavobacteriales(0.1218),f:Flavobacteriaceae(0.0454),g:Riemerella(0.0119);
d:Bacteria(1.0000),p:Firmicutes(0.9899),c:Clostridia(0.8348),o:Clostridiales(0.4658),f:Clostridiales_Incertae_Sedis_XII(0.2263),g:Acidaminobacter(0.0523);
d:Bacteria(1.0000),p:Firmicutes(0.9930),c:Clostridia(0.6471),o:Clostridiales(0.3394),f:Lachnospiraceae(0.1525),g:Clostridium_XlVa(0.0215);

d:Bacteria(1.0000),p:Firmicutes(0.9990),c:Clostridia(0.9631),o:Clostridiales(0.9317),f:Clostridiales_Incertae_Sedis_XIII(0.6928),g:Mogibacterium(0.5327);
d:Bacteria(1.0000),p:Firmicutes(0.5785),c:Clostridia(0.2725),o:Clostridiales(0.1218),f:Clostridiales_Incertae_Sedis_III(0.0140),g:Caldicellulosiruptor(0.0060);
d:Bacteria(1.0000),p:Firmicutes(0.9978),c:Clostridia(0.9486),o:Clostridiales(0.8757),f:Ruminococcaceae(0.6553),g:Clostridium_IV(0.0751);
d:Bacteria(1.0000),p:Actinobacteria(0.9922),c:Actinobacteria(0.8746),o:Coriobacteriales(0.6422),f:Coriobacteriaceae(0.4677),g:Slackia(0.0865);
d:Bacteria(1.0000),p:Firmicutes(0.8857),c:Clostridia(0.6471),o:Clostridiales(0.3394),f:Ruminococcaceae(0.1097),g:Intestinimonas(0.0143);
d:Bacteria(1.0000),p:Firmicutes(0.9991),c:Clostridia(0.9486),o:Clostridiales(0.8757),f:Eubacteriaceae(0.4677),g:Eubacterium(0.2194);
d:Bacteria(1.0000),p:Firmicutes(0.6840),c:Erysipelotrichia(0.3342),o:Erysipelotrichales(0.1579),f:Erysipelotrichaceae(0.0668),g:Holdemania(0.0119);
d:Bacteria(1.0000),p:Firmicutes(0.8147),c:Erysipelotrichia(0.3967),o:Erysipelotrichales(0.1942),f:Erysipelotrichaceae(0.0883),g:Erysipelotrichaceae_incertae_sedis(0.0167);
d:Bacteria(1.0000),p:Firmicutes(0.9946),c:Clostridia(0.9023),o:Clostridiales(0.7746),f:Ruminococcaceae(0.4677),g:Ruminococcus(0.0751);
d:Bacteria(1.0000),p:Firmicutes(0.4731),c:Clostridia(0.2113),o:Clostridiales(0.0945),f:Ruminococcaceae(0.0218),g:Clostridium_III(0.0077);
d:Bacteria(1.0000),p:Firmicutes(0.9802),c:Clostridia(0.7097),o:Clostridiales(0.3775),f:Gracilibacteraceae(0.1311),g:Gracilibacter(0.0215);
d:Bacteria(1.0000),p:Proteobacteria(0.8384),c:Alphaproteobacteria(0.5219),o:Rhodospirillales(0.1579),f:Rhodospirillaceae(0.0668),g:Insolitispirillum(0.0077);
d:Bacteria(1.0000),p:Firmicutes(0.9991),c:Clostridia(0.9391),o:Clostridiales(0.8477),f:Ruminococcaceae(0.6928),g:Anaerobacterium(0.3238);
d:Bacteria(1.0000),p:Firmicutes(0.9987),c:Clostridia(0.9595),o:Clostridiales(0.9177),f:Lachnospiraceae(0.5804),g:Syntrophococcus(0.2194);
d:Bacteria(1.0000),p:Firmicutes(0.8857),c:Clostridia(0.7097),o:Clostridiales(0.3775),f:Ruminococcaceae(0.1097),g:Intestinimonas(0.0167);
d:Bacteria(1.0000),p:Firmicutes(0.9566),c:Clostridia(0.8348),o:Clostridiales(0.3775),f:Lachnospiraceae(0.1097),g:Clostridium_XlVa(0.0191);
d:Bacteria(1.0000),p:Firmicutes(0.9329),c:Clostridia(0.7723),o:Clostridiales(0.4216),f:Eubacteriaceae(0.1311),g:Eubacterium(0.0215);
d:Bacteria(1.0000),p:Firmicutes(0.9993),c:Clostridia(0.9391),o:Clostridiales(0.8477),f:Lachnospiraceae(0.7302),g:Parasporobacterium(0.3760);
d:Bacteria(1.0000),p:Firmicutes(0.9938),c:Clostridia(0.8931),o:Clostridiales(0.7305),f:Lachnospiraceae(0.3068),g:Marvinbryantia(0.0409);
d:Bacteria(1.0000),p:Proteobacteria(0.1875),c:Deltaproteobacteria(0.0345),o:Bdellovibrionales(0.0136),f:Bdellovibrionaceae(0.0061),g:Vampirovibrio(0.0043);
d:Bacteria(1.0000),p:Actinobacteria(0.9802),c:Actinobacteria(0.8470),o:Coriobacteriales(0.5099),f:Coriobacteriaceae(0.3470),g:Gordonibacter(0.0215);
d:Bacteria(1.0000),p:Bacteroidetes(0.9907),c:Sphingobacteriia(0.5219),o:Sphingobacteriales(0.2668),f:Sphingobacteriaceae(0.1311),g:Parapedobacter(0.0191);
d:Bacteria(1.0000),p:Firmicutes(0.9899),c:Clostridia(0.5845),o:Clostridiales(0.3031),f:Lachnospiraceae(0.1097),g:Eisenbergiella(0.0191);
d:Bacteria(1.0000),p:Firmicutes(0.7674),c:Clostridia(0.3967),o:Clostridiales(0.1942),f:Clostridiaceae_1(0.0883),g:Caloramator(0.0167);
d:Bacteria(1.0000),p:Firmicutes(0.7911),c:Clostridia(0.4593),o:Clostridiales(0.2305),f:Ruminococcaceae(0.0454),g:Intestinimonas(0.0094);
d:Bacteria(1.0000),p:Firmicutes(0.8857),c:Clostridia(0.5219),o:Clostridiales(0.2668),f:Ruminococcaceae(0.0668),g:Hydrogenoanaerobacterium(0.0094);
d:Bacteria(1.0000),p:Firmicutes(0.9987),c:Clostridia(0.9777),o:Clostridiales(0.9693),f:Lachnospiraceae(0.8943),g:Shuttleworthia(0.5751);
d:Bacteria(1.0000),p:Firmicutes(0.9954),c:Clostridia(0.9299),o:Clostridiales(0.8197),f:Ruminococcaceae(0.5430),g:Ruminococcus(0.1150);
d:Bacteria(1.0000),p:Firmicutes(0.9988),c:Clostridia(0.9966),o:Clostridiales(0.9966),f:Lachnospiraceae(0.9919),g:Cellulosilyticum(0.9189);
d:Bacteria(1.0000),p:Firmicutes(0.9946),c:Bacilli(0.7097),o:Lactobacillales(0.3775),f:Lactobacillaceae(0.2263),g:Lactobacillus(0.0523);
d:Bacteria(1.0000),p:Proteobacteria(0.9991),c:Betaproteobacteria(0.9704),o:Rhodocyclales(0.8057),f:Rhodocyclaceae(0.6179),g:Azoarcus(0.4282);
d:Bacteria(1.0000),p:Firmicutes(0.9989),c:Clostridia(0.9704),o:Clostridiales(0.9597),f:Lachnospiraceae(0.8425),g:Pseudobutyrivibrio(0.4804);
d:Bacteria(1.0000),p:Fibrobacteres(0.9986),c:Fibrobacteria(0.9522),o:Fibrobacterales(0.8897),f:Fibrobacteraceae(0.8425),g:Fibrobacter(0.6782);
d:Bacteria(1.0000),p:Firmicutes(0.9978),c:Clostridia(0.9486),o:Clostridiales(0.8757),f:Lachnospiraceae(0.6553),g:Cellulosilyticum(0.0865);
d:Bacteria(1.0000),p:Firmicutes(0.9987),c:Clostridia(0.9886),o:Clostridiales(0.9757),f:Lachnospiraceae(0.9191),g:Clostridium_XlVb(0.5751);
d:Bacteria(1.0000),p:Firmicutes(0.9907),c:Clostridia(0.8562),o:Clostridiales(0.5540),f:Ruminococcaceae(0.2263),g:Acetivibrio(0.0191);
d:Bacteria(1.0000),p:Firmicutes(0.9991),c:Erysipelotrichia(0.9668),o:Erysipelotrichales(0.9457),f:Erysipelotrichaceae(0.9026),g:Erysipelotrichaceae_incertae_sedis(0.7814);
d:Bacteria(1.0000),p:Firmicutes(0.8147),c:Clostridia(0.5219),o:Clostridiales(0.2668),f:Ruminococcaceae(0.0883),g:Clostridium_III(0.0167);
d:Bacteria(1.0000),p:Firmicutes(0.6840),c:Erysipelotrichia(0.3342),o:Erysipelotrichales(0.1579),f:Erysipelotrichaceae(0.0668),g:Clostridium_XVIII(0.0094);
d:Bacteria(1.0000),p:Spirochaetes(0.9991),c:Spirochaetia(0.9668),o:Spirochaetales(0.9457),f:Spirochaetaceae(0.9026),g:Treponema(0.5751);
d:Bacteria(1.0000),p:Tenericutes(0.2622),c:Mollicutes(0.0888),o:Acholeplasmatales(0.0400),f:Acholeplasmataceae(0.0140),g:Acholeplasma(0.0060);
d:Bacteria(1.0000),p:Firmicutes(0.8147),c:Clostridia(0.5219),o:Clostridiales(0.2668),f:Ruminococcaceae(0.0668),g:Intestinimonas(0.0143);
d:Bacteria(1.0000),p:Lentisphaerae(0.9962),c:Lentisphaeria(0.9391),o:Victivallales(0.8477),f:Victivallaceae(0.7302),g:Victivallis(0.5751);
d:Bacteria(1.0000),p:Firmicutes(0.9991),c:Clostridia(0.9595),o:Clostridiales(0.8897),f:Ruminococcaceae(0.6553),g:Papillibacter(0.1672);
d:Bacteria(1.0000),p:Firmicutes(0.9986),c:Clostridia(0.8746),o:Clostridiales(0.6422),f:Lachnospiraceae(0.4677),g:Lachnospiracea_incertae_sedis(0.0865);
d:Bacteria(1.0000),p:Firmicutes(0.9985),c:Clostridia(0.9926),o:Clostridiales(0.9820),f:Clostridiaceae_1(0.9191),g:Clostridium_sensu_stricto(0.6095);
d:Bacteria(1.0000),p:Bacteroidetes(0.9802),c:Sphingobacteriia(0.4593),o:Sphingobacteriales(0.2305),f:Sphingobacteriaceae(0.1097),g:Pedobacter(0.0143);
d:Bacteria(1.0000),p:Firmicutes(0.8857),c:Clostridia(0.7097),o:Clostridiales(0.3775),f:Eubacteriaceae(0.0668),g:Eubacterium(0.0143);
d:Bacteria(1.0000),p:Verrucomicrobia(0.8857),c:Subdivision5(0.7097),g:Subdivision5_genera_incertae_sedis(0.0523);
d:Bacteria(1.0000),p:Tenericutes(0.2622),c:Mollicutes(0.0888),o:Anaeroplasmatales(0.0400),f:Anaeroplasmataceae(0.0140),g:Asteroleplasma(0.0060);

d:Bacteria(1.0000),p:Firmicutes(0.8620),c:Clostridia(0.5845),o:Clostridiales(0.3031),f:Gracilibacteraceae(0.1097),g:Gracilibacter(0.0191);
d:Bacteria(1.0000),p:Firmicutes(0.9990),c:Bacilli(0.8931),o:Lactobacillales(0.7305),f:Lactobacillaceae(0.5430),g:Lactobacillus(0.3238);
d:Bacteria(1.0000),p:Fusobacteria(0.9983),c:Fusobacteriia(0.9952),o:Fusobacteriales(0.9952),f:Leptotrichiaceae(0.9026),g:Leptotrichia(0.3760);
d:Bacteria(1.0000),p:Firmicutes(0.9802),c:Clostridia(0.8562),o:Clostridiales(0.5540),f:Clostridiaceae_1(0.3068),g:Oxobacter(0.0523);
d:Bacteria(1.0000),p:Firmicutes(0.2622),c:Clostridia(0.0888),o:Clostridiales(0.0400),f:Clostridiales_Incertae_Sedis_XII(0.0140),g:Guggenheimella(0.0043);
d:Bacteria(1.0000),p:Firmicutes(0.9329),c:Clostridia(0.7723),o:Natranaerobiales(0.2668),f:Natranaerobiaceae(0.1311),g:Dethiobacter(0.0215);
d:Bacteria(1.0000),p:Fibrobacteres(0.9978),c:Fibrobacteria(0.9486),o:Fibrobacterales(0.8757),f:Fibrobacteraceae(0.8051),g:Fibrobacter(0.6438);
d:Bacteria(1.0000),p:Firmicutes(0.9991),c:Clostridia(0.9023),o:Clostridiales(0.7746),f:Lachnospiraceae(0.5430),g:Parasporobacterium(0.1672);
d:Bacteria(1.0000),p:Firmicutes(0.9991),c:Clostridia(0.9391),o:Clostridiales(0.8477),f:Lachnospiraceae(0.4677),g:Clostridium_XlVa(0.1672);
d:Bacteria(1.0000),p:Bacteroidetes(0.9989),c:Bacteroidia(0.9207),o:Bacteroidales(0.8197),f:Prevotellaceae(0.4677),g:Prevotella(0.0637);
d:Bacteria(1.0000),p:Firmicutes(0.9093),c:Clostridia(0.6471),o:Clostridiales(0.3394),f:Lachnospiraceae(0.1311),g:Anaerostipes(0.0167);
d:Bacteria(1.0000),p:Verrucomicrobia(0.8147),c:Subdivision5(0.5219),g:Subdivision5_genera_incertae_sedis(0.0215);
d:Bacteria(1.0000),p:Firmicutes(0.9986),c:Clostridia(0.9522),o:Clostridiales(0.8897),f:Clostridiaceae_1(0.6553),g:Clostridium_sensu_stricto(0.4804);
d:Bacteria(1.0000),p:Firmicutes(0.8384),c:Clostridia(0.5845),o:Clostridiales(0.3031),f:Ruminococcaceae(0.1097),g:Intestinimonas(0.0143);
d:Bacteria(1.0000),p:Firmicutes(0.9984),c:Bacilli(0.9937),o:Bacillales(0.9736),f:Staphylococcaceae(0.9274),g:Macrococcus(0.6095);
d:Bacteria(1.0000),p:Firmicutes(0.9990),c:Clostridia(0.9631),o:Clostridiales(0.9317),f:Lachnospiraceae(0.7302),g:Clostridium_XlVa(0.5751);
d:Bacteria(1.0000),p:Verrucomicrobia(0.8620),c:Subdivision5(0.6471),g:Subdivision5_genera_incertae_sedis(0.0409);
d:Bacteria(1.0000),p:Cyanobacteria/Chloroplast(0.9999),c:Cyanobacteria(0.9995),f:Family_I(0.9935),g:GpI(0.9935);
d:Bacteria(1.0000),p:Firmicutes(0.9992),c:Clostridia(0.9704),o:Clostridiales(0.9597),f:Lachnospiraceae(0.8425),g:Clostridium_XlVa(0.5327);
d:Bacteria(1.0000),p:Verrucomicrobia(0.8857),c:Subdivision5(0.7097),g:Subdivision5_genera_incertae_sedis(0.0523);
d:Bacteria(1.0000),p:Firmicutes(0.9566),c:Clostridia(0.8470),o:Clostridiales(0.4658),f:Ruminococcaceae(0.1861),g:Intestinimonas(0.0215);
d:Bacteria(1.0000),p:Firmicutes(0.9899),c:Clostridia(0.7097),o:Clostridiales(0.3775),f:Lachnospiraceae(0.1097),g:Clostridium_XlVb(0.0191);
d:Bacteria(1.0000),p:Bacteroidetes(0.8147),c:Bacteroidia(0.4593),o:Bacteroidales(0.2305),f:Bacteroidaceae(0.0296),g:Bacteroides(0.0094);
d:Bacteria(1.0000),p:Firmicutes(0.1875),c:Clostridia(0.0345),o:Clostridiales(0.0136),f:Ruminococcaceae(0.0061),g:Ruminococcus(0.0043);
d:Bacteria(1.0000),p:Proteobacteria(0.9093),c:Alphaproteobacteria(0.7723),o:Rhodospirillales(0.2668),f:Rhodospirillaceae(0.1311),g:Fodinicurvata(0.0167);
d:Bacteria(1.0000),p:Firmicutes(0.9930),c:Clostridia(0.9023),o:Clostridiales(0.7305),f:Ruminococcaceae(0.4275),g:Intestinimonas(0.0523);
d:Bacteria(1.0000),p:Bacteroidetes(0.7674),c:Sphingobacteriia(0.0345),o:Sphingobacteriales(0.0136),f:Sphingobacteriaceae(0.0061),g:Pedobacter(0.0043);
d:Bacteria(1.0000),p:Bacteroidetes(0.9802),c:Sphingobacteriia(0.3342),o:Sphingobacteriales(0.1579),f:Sphingobacteriaceae(0.0668),g:Parapedobacter(0.0119);
d:Bacteria(1.0000),p:Bacteroidetes(0.9970),c:Bacteroidia(0.6471),o:Bacteroidales(0.3394),f:Bacteroidaceae(0.0668),g:Bacteroides(0.0143);
d:Bacteria(1.0000),p:Bacteroidetes(0.9093),c:Sphingobacteriia(0.3342),o:Sphingobacteriales(0.1579),f:Sphingobacteriaceae(0.0668),g:Parapedobacter(0.0094);
d:Bacteria(1.0000),p:Firmicutes(0.9994),c:Clostridia(0.9741),o:Clostridiales(0.9672),f:Lachnospiraceae(0.8051),g:Clostridium_XlVa(0.6438);
d:Bacteria(1.0000),p:Cyanobacteria/Chloroplast(0.9990),c:Cyanobacteria(0.8746),f:Family_I(0.3068),g:GpI(0.0751);
d:Bacteria(1.0000),p:Firmicutes(0.9993),c:Clostridia(0.8562),o:Clostridiales(0.5540),f:Clostridiaceae_1(0.3470),g:Anaerosporobacter(0.0865);
d:Bacteria(1.0000),p:Firmicutes(0.9993),c:Clostridia(0.9207),o:Clostridiales(0.8197),f:Lachnospiraceae(0.3872),g:Lachnospiracea_incertae_sedis(0.0865);
d:Bacteria(1.0000),p:Firmicutes(0.9990),c:Clostridia(0.9631),o:Clostridiales(0.9317),f:Ruminococcaceae(0.7302),g:Oscillibacter(0.5327);
d:Bacteria(1.0000),p:Firmicutes(0.9987),c:Clostridia(0.9207),o:Clostridiales(0.8197),f:Lachnospiraceae(0.5430),g:Clostridium_XlVa(0.3238);
d:Bacteria(1.0000),p:Firmicutes(0.9991),c:Clostridia(0.9023),o:Clostridiales(0.7746),f:Lachnospiraceae(0.4275),g:Ruminococcus2(0.1150);
d:Bacteria(1.0000),p:Bacteroidetes(0.9984),c:Bacteroidia(0.9631),o:Bacteroidales(0.9317),f:Prevotellaceae(0.7302),g:Prevotella(0.3760);
d:Bacteria(1.0000),p:Firmicutes(0.9946),c:Clostridia(0.8931),o:Clostridiales(0.7305),f:Lachnospiraceae(0.4275),g:Cellulosilyticum(0.0523);
d:Bacteria(1.0000),p:Firmicutes(0.9989),c:Clostridia(0.9486),o:Clostridiales(0.8757),f:Lachnospiraceae(0.6179),g:Clostridium_XlVa(0.2194);
d:Bacteria(1.0000),p:Bacteroidetes(0.8384),c:Sphingobacteriia(0.2725),o:Sphingobacteriales(0.1218),f:Sphingobacteriaceae(0.0454),g:Pedobacter(0.0094);
d:Bacteria(1.0000),p:Bacteroidetes(0.8857),c:Bacteroidia(0.2725),o:Bacteroidales(0.1218),f:Prevotellaceae(0.0218),g:Prevotella(0.0077);
d:Bacteria(1.0000),p:Firmicutes(0.9990),c:Clostridia(0.9741),o:Clostridiales(0.9672),f:Ruminococcaceae(0.8943),g:Oscillibacter(0.5327);
d:Bacteria(1.0000),p:Spirochaetes(0.9993),c:Spirochaetia(0.9559),o:Spirochaetales(0.9037),f:Spirochaetaceae(0.8778),g:Treponema(0.5751);
d:Bacteria(1.0000),p:Fibrobacteres(0.9914),c:Fibrobacteria(0.8838),o:Fibrobacterales(0.6864),f:Fibrobacteraceae(0.5055),g:Fibrobacter(0.2716);
d:Bacteria(1.0000),p:Fibrobacteres(0.9986),c:Fibrobacteria(0.9522),o:Fibrobacterales(0.8897),f:Fibrobacteraceae(0.8425),g:Fibrobacter(0.6782);
d:Bacteria(1.0000),p:Verrucomicrobia(0.8384),c:Subdivision5(0.5845),g:Subdivision5_genera_incertae_sedis(0.0295);
d:Bacteria(1.0000),p:Spirochaetes(0.9991),c:Spirochaetia(0.9668),o:Spirochaetales(0.9457),f:Spirochaetaceae(0.9026),g:Treponema(0.5327);
d:Bacteria(1.0000),p:Firmicutes(0.9566),c:Clostridia(0.8470),o:Clostridiales(0.3394),f:Ruminococcaceae(0.1861),g:Intestinimonas(0.0215);
d:Bacteria(1.0000),p:Firmicutes(0.9989),c:Clostridia(0.8931),o:Clostridiales(0.7305),f:Lachnospiraceae(0.5055),g:Clostridium_XlVa(0.2194);

d:Bacteria(1.0000),p:Tenericutes(0.5785),c:Mollicutes(0.2725),o:Acholeplasmatales(0.0673),f:Acholeplasmataceae(0.0218),g:Acholeplasma(0.0077);
d:Bacteria(1.0000),p:Firmicutes(0.8147),c:Clostridia(0.5219),o:Clostridiales(0.2305),f:Ruminococcaceae(0.0218),g:Intestinimonas(0.0077);
d:Bacteria(1.0000),p:Bacteroidetes(0.9093),c:Sphingobacteriia(0.2113),o:Sphingobacteriales(0.0945),f:Sphingobacteriaceae(0.0296),g:Solitalea(0.0094);
d:Bacteria(1.0000),p:Bacteroidetes(0.9990),c:Bacteroidia(0.8746),o:Bacteroidales(0.6422),f:Prevotellaceae(0.2263),g:Prevotella(0.0523);
d:Bacteria(1.0000),p:Firmicutes(0.9930),c:Clostridia(0.8562),o:Clostridiales(0.5540),f:Lachnospiraceae(0.1861),g:Anaerostipes(0.0409);
d:Bacteria(1.0000),p:Firmicutes(0.5785),c:Clostridia(0.2725),o:Clostridiales(0.0400),f:Ruminococcaceae(0.0140),g:Intestinimonas(0.0060);
d:Bacteria(1.0000),p:Bacteroidetes(0.9984),c:Bacteroidia(0.9947),o:Bacteroidales(0.9947),f:Prevotellaceae(0.8425),g:Prevotella(0.3238);
d:Bacteria(1.0000),p:Firmicutes(0.7674),c:Clostridia(0.1500),o:Clostridiales(0.0673),f:Clostridiales_Incertae_Sedis_XIII(0.0218),g:Anaerovorax(0.0077);
d:Bacteria(1.0000),p:Bacteroidetes(0.8147),c:Sphingobacteriia(0.0888),o:Sphingobacteriales(0.0400),f:Sphingobacteriaceae(0.0140),g:Pedobacter(0.0060);
d:Bacteria(1.0000),p:Firmicutes(0.8620),c:Negativicutes(0.5845),o:Selenomonadales(0.3031),f:Acidaminococcaceae(0.1525),g:Acidaminococcus(0.0119);
d:Bacteria(1.0000),p:Firmicutes(0.3677),c:Erysipelotrichia(0.1500),o:Erysipelotrichales(0.0673),f:Erysipelotrichaceae(0.0218),g:Clostridium_XVIII(0.0077);
d:Bacteria(1.0000),p:Firmicutes(0.8857),c:Clostridia(0.7097),o:Clostridiales(0.3031),f:Ruminococcaceae(0.1525),g:Clostridium_IV(0.0119);
d:Bacteria(1.0000),p:Bacteroidetes(0.8857),c:Sphingobacteriia(0.2113),o:Sphingobacteriales(0.0945),f:Sphingobacteriaceae(0.0296),g:Parapedobacter(0.0077);
d:Bacteria(1.0000),p:Firmicutes(0.9093),c:Clostridia(0.7723),o:Clostridiales(0.4216),f:Clostridiaceae_1(0.1311),g:Clostridium_sensu_stricto(0.0215);
d:Bacteria(1.0000),p:Tenericutes(0.8620),c:Mollicutes(0.6471),o:Anaeroplasmatales(0.2305),f:Anaeroplasmataceae(0.1097),g:Anaeroplasma(0.0191);
d:Bacteria(1.0000),p:Verrucomicrobia(0.9093),c:Subdivision5(0.7723),g:Subdivision5_genera_incertae_sedis(0.0637);
d:Bacteria(1.0000),p:Firmicutes(0.9991),c:Clostridia(0.9559),o:Clostridiales(0.9037),f:Peptostreptococcaceae(0.5804),g:Clostridium_XI(0.3760);
d:Bacteria(1.0000),p:Spirochaetes(0.8147),c:Spirochaetia(0.5219),o:Spirochaetales(0.2668),f:Spirochaetaceae(0.1311),g:Treponema(0.0094);
d:Bacteria(1.0000),p:Bacteroidetes(0.9329),c:Bacteroidia(0.2113),o:Bacteroidales(0.0945),f:Marinilabiaceae(0.0296),g:Marinilabilia(0.0094);
d:Bacteria(1.0000),p:Bacteroidetes(0.9984),c:Bacteroidia(0.9937),o:Bacteroidales(0.9926),f:Prevotellaceae(0.7302),g:Prevotella(0.5751);
d:Bacteria(1.0000),p:Bacteroidetes(0.9802),c:Bacteroidia(0.3342),o:Bacteroidales(0.1579),f:Marinilabiaceae(0.0668),g:Anaerophaga(0.0143);

Table S6

OTUs with dirrerential occurrence in Geladas relative to other primates (logFC=log fold change, FDR=false discovery rate)

Taxonomic assignment (probability of correct assignment in parentheses)	logFC	PValue	FDR
d:Bacteria(1.0000),p:Verrucomicrobia(0.8620),c:Subdivision5(0.6471),g:Subdivision5_genera_incertae_sedis(0.0409);	12.57012	3.05E-196	1.40E-193
d:Bacteria(1.0000),p:Verrucomicrobia(0.7674),c:Subdivision5(0.3967),g:Subdivision5_genera_incertae_sedis(0.0167);	11.08797	6.00E-194	1.37E-191
d:Bacteria(1.0000),p:Firmicutes(0.8620),c:Clostridia(0.6471),o:Clostridiales(0.2305),f:Ruminococcaceae(0.1097),g:Clostridium_IV(0.0191);	13.64319	3.81E-191	5.82E-189
d:Bacteria(1.0000),p:Bacteroidetes(0.8620),c:Sphingobacteriia(0.2725),o:Sphingobacteriales(0.1218),f:Sphingobacteriaceae(0.0454),g:Pedobacter(0.0094);	13.39467	2.11E-189	2.42E-187
d:Bacteria(1.0000),p:Bacteroidetes(0.9802),c:Sphingobacteriia(0.6471),o:Sphingobacteriales(0.3394),f:Sphingobacteriaceae(0.1861),g:Pedobacter(0.0167);	12.61787	2.12E-183	1.94E-181
d:Bacteria(1.0000),p:Firmicutes(0.8147),c:Clostridia(0.5219),o:Clostridiales(0.1579),f:Ruminococcaceae(0.0668),g:Intestinimonas(0.0119);	13.68362	1.22E-180	9.35E-179
d:Bacteria(1.0000),p:Bacteroidetes(0.9329),c:Sphingobacteriia(0.2725),o:Sphingobacteriales(0.1218),f:Sphingobacteriaceae(0.0454),g:Parapedobacter(0.0119);	13.04268	2.00E-174	1.31E-172
d:Bacteria(1.0000),p:Bacteroidetes(0.9802),c:Sphingobacteriia(0.4593),o:Sphingobacteriales(0.2305),f:Sphingobacteriaceae(0.1097),g:Pedobacter(0.0143);	11.96507	1.59E-164	9.08E-163
d:Bacteria(1.0000),p:Tenericutes(0.9930),c:Mollicutes(0.9023),o:Anaeroplasmatales(0.5981),f:Anaeroplasmataceae(0.4275),g:Anaeroplasma(0.1672);	11.8341	3.98E-157	2.03E-155
d:Bacteria(1.0000),p:Verrucomicrobia(0.9093),c:Subdivision5(0.7723),g:Subdivision5_genera_incertae_sedis(0.0637);	12.50873	1.46E-153	6.67E-152
d:Bacteria(1.0000),p:Bacteroidetes(0.9329),c:Sphingobacteriia(0.3967),o:Sphingobacteriales(0.1942),f:Sphingobacteriaceae(0.0883),g:Parapedobacter(0.0119);	13.05164	2.10E-152	8.75E-151
d:Bacteria(1.0000),p:Firmicutes(0.8857),c:Clostridia(0.7097),o:Clostridiales(0.3775),f:Eubacteriaceae(0.0668),g:Eubacterium(0.0143);	11.75052	6.39E-141	2.44E-139
d:Bacteria(1.0000),p:Verrucomicrobia(0.2622),c:Subdivision5(0.0888),g:Subdivision5_genera_incertae_sedis(0.0060);	11.92309	1.15E-140	4.05E-139
d:Bacteria(1.0000),p:Spirochaetes(0.8857),c:Spirochaetia(0.7097),o:Spirochaetales(0.3775),f:Spirochaetaceae(0.2263),g:Treponema(0.0191);	13.10518	6.19E-140	2.03E-138
d:Bacteria(1.0000),p:Bacteroidetes(0.9907),c:Sphingobacteriia(0.5845),o:Sphingobacteriales(0.3031),f:Sphingobacteriaceae(0.1525),g:Solitalea(0.0167);	12.30376	2.41E-136	7.37E-135
d:Bacteria(1.0000),p:Bacteroidetes(0.9566),c:Sphingobacteriia(0.2725),o:Sphingobacteriales(0.1218),f:Sphingobacteriaceae(0.0454),g:Solitalea(0.0119);	12.35404	1.14E-134	3.26E-133
d:Bacteria(1.0000),p:Fibrobacteres(0.9991),c:Fibrobacteria(0.9704),o:Fibrobacterales(0.9597),f:Fibrobacteraceae(0.9109),g:Fibrobacter(0.8502);	12.75264	2.14E-133	5.61E-132
d:Bacteria(1.0000),p:Bacteroidetes(0.9938),c:Bacteroidia(0.8562),o:Bacteroidales(0.5540),f:Porphyromonadaceae(0.1525),g:Paludibacter(0.0295);	13.31761	2.20E-133	5.61E-132
d:Bacteria(1.0000),p:Bacteroidetes(0.9899),c:Bacteroidia(0.5845),o:Bacteroidales(0.3031),f:Prevotellaceae(0.0883),g:Paraprevotella(0.0143);	12.49455	1.02E-132	2.46E-131
d:Bacteria(1.0000),p:Spirochaetes(0.8857),c:Spirochaetia(0.7097),o:Spirochaetales(0.3775),f:Spirochaetaceae(0.2263),g:Treponema(0.0094);	12.36244	9.97E-132	2.28E-130
d:Bacteria(1.0000),p:Tenericutes(0.1875),c:Mollicutes(0.0345),o:Anaeroplasmatales(0.0136),f:Anaeroplasmataceae(0.0061),g:Asteroleplasma(0.0043);	12.95843	2.33E-131	5.07E-130
d:Bacteria(1.0000),p:Tenericutes(0.9930),c:Mollicutes(0.9023),o:Anaeroplasmatales(0.5540),f:Anaeroplasmataceae(0.3872),g:Anaeroplasma(0.1150);	13.34203	5.26E-130	1.09E-128
d:Bacteria(1.0000),p:Firmicutes(0.5785),c:Clostridia(0.2725),o:Clostridiales(0.1218),f:Clostridiaceae_4(0.0218),g:Thermotalea(0.0077);	12.78518	1.08E-128	2.15E-127
d:Bacteria(1.0000),p:Bacteroidetes(0.9984),c:Bacteroidia(0.9937),o:Bacteroidales(0.9926),f:Prevotellaceae(0.6179),g:Prevotella(0.3760);	12.25736	5.80E-128	1.11E-126
d:Bacteria(1.0000),p:Verrucomicrobia(0.9938),c:Subdivision5(0.9115),g:Subdivision5_genera_incertae_sedis(0.4282);	11.33519	1.02E-125	1.87E-124
d:Bacteria(1.0000),p:Bacteroidetes(0.9566),c:Bacteroidia(0.2725),o:Bacteroidales(0.1218),f:Marinilabiliaceae(0.0296),g:Alkalitalea(0.0094);	12.04905	8.42E-122	1.48E-120
d:Bacteria(1.0000),p:Spirochaetes(0.9990),c:Spirochaetia(0.9777),o:Spirochaetales(0.9693),f:Spirochaetaceae(0.9274),g:Treponema(0.7126);	11.82571	1.86E-119	3.16E-118
d:Bacteria(1.0000),p:Bacteroidetes(0.9802),c:Sphingobacteriia(0.3342),o:Sphingobacteriales(0.1579),f:Sphingobacteriaceae(0.0668),g:Solitalea(0.0143);	12.04879	2.84E-119	4.65E-118
d:Bacteria(1.0000),p:Bacteroidetes(0.9899),c:Bacteroidia(0.5845),o:Bacteroidales(0.3031),f:Porphyromonadaceae(0.1525),g:Barnesiella(0.0167);	12.18043	6.97E-119	1.10E-117
d:Bacteria(1.0000),p:Bacteroidetes(0.9093),c:Sphingobacteriia(0.2725),o:Sphingobacteriales(0.1218),f:Sphingobacteriaceae(0.0454),g:Pedobacter(0.0094);	12.36012	4.17E-118	6.37E-117
d:Bacteria(1.0000),p:Bacteroidetes(0.9566),c:Sphingobacteriia(0.3342),o:Sphingobacteriales(0.1579),f:Sphingobacteriaceae(0.0668),g:Pedobacter(0.0119);	12.03936	2.90E-117	4.28E-116
d:Bacteria(1.0000),p:Firmicutes(0.9566),c:Clostridia(0.5845),o:Clostridiales(0.3031),f:Clostridiales_Incertae_Sedis_XIII(0.0883),g:Anaerovorax(0.0167);	12.49784	2.21E-116	3.17E-115
d:Bacteria(1.0000),p:Bacteroidetes(0.9907),c:Sphingobacteriia(0.3342),o:Sphingobacteriales(0.1579),f:Sphingobacteriaceae(0.0668),g:Pedobacter(0.0143);	12.5634	4.98E-115	6.91E-114
d:Archaea(1.0000),p:Euryarchaeota(0.9930),c:Thermoplasmata(0.8562),o:Methanomassiliicoccales(0.4216),f:Methanomassiliicoccaceae(0.2665),g:Methanomassiliicoccus(0.0637);	11.90703	6.89E-114	9.28E-113
d:Bacteria(1.0000),p:Firmicutes(0.9978),c:Clostridia(0.9486),o:Clostridiales(0.8757),f:Ruminococcaceae(0.5804),g:Oscillibacter(0.0865);	12.53035	1.07E-113	1.40E-112
d:Bacteria(1.0000),p:Bacteroidetes(0.9802),c:Sphingobacteriia(0.5845),o:Sphingobacteriales(0.3031),f:Sphingobacteriaceae(0.1525),g:Parapedobacter(0.0143);	12.78952	1.25E-112	1.58E-111
d:Bacteria(1.0000),p:Verrucomicrobia(0.9566),c:Subdivision5(0.8470),g:Subdivision5_genera_incertae_sedis(0.0865);	10.40061	1.19E-111	1.47E-110
d:Bacteria(1.0000),p:Bacteroidetes(0.8147),c:Sphingobacteriia(0.0315),o:Sphingobacteriales(0.0123),f:Sphingobacteriaceae(0.0042),g:Sphingobacterium(0.0034);	11.94548	3.60E-111	4.33E-110
d:Bacteria(1.0000),p:Bacteroidetes(0.9802),c:Sphingobacteriia(0.3342),o:Sphingobacteriales(0.1579),f:Sphingobacteriaceae(0.0668),g:Solitalea(0.0143);	12.57493	8.91E-109	1.05E-107
d:Bacteria(1.0000),p:Firmicutes(0.9329),c:Clostridia(0.7723),o:Clostridiales(0.3394),f:Ruminococcaceae(0.1311),g:Intestinimonas(0.0167);	11.63612	1.11E-108	1.27E-107
d:Bacteria(1.0000),p:Verrucomicrobia(0.8857),c:Subdivision5(0.7097),g:Subdivision5_genera_incertae_sedis(0.0523);	11.706	7.39E-107	8.25E-106
d:Bacteria(1.0000),p:Bacteroidetes(0.8147),c:Bacteroidia(0.4593),o:Bacteroidales(0.2305),f:Bacteroidaceae(0.0296),g:Bacteroides(0.0094);	11.03388	1.06E-106	1.15E-105
d:Bacteria(1.0000),p:Bacteroidetes(0.9985),c:Bacteroidia(0.9631),o:Bacteroidales(0.9317),f:Prevotellaceae(0.6553),g:Prevotella(0.4804);	13.14114	1.79E-106	1.91E-105
d:Bacteria(1.0000),p:Verrucomicrobia(0.8857),c:Subdivision5(0.7097),g:Subdivision5_genera_incertae_sedis(0.0523);	11.45745	1.22E-105	1.27E-104
d:Bacteria(0.9753),p:Bacteroidetes(0.5785),c:Sphingobacteriia(0.0315),o:Sphingobacteriales(0.0123),f:Sphingobacteriaceae(0.0042),g:Sphingobacterium(0.0034);	12.17261	3.87E-104	3.94E-103
d:Bacteria(1.0000),p:Bacteroidetes(0.9899),c:Bacteroidia(0.7723),o:Bacteroidales(0.4216),f:Prevotellaceae(0.0883),g:Paraprevotella(0.0143);	11.50094	2.00E-100	1.99E-99
d:Bacteria(1.0000),p:Bacteroidetes(0.9329),c:Sphingobacteriia(0.2725),o:Sphingobacteriales(0.1218),f:Sphingobacteriaceae(0.0454),g:Pedobacter(0.0094);	11.44956	4.70E-100	4.58E-99

d:Bacteria(1.0000),p:Firmicutes(0.9566),c:Clostridia(0.6471),o:Clostridiales(0.3394),f:Lachnospiraceae(0.1311),g:Clostridium_XlVa(0.0191);	11.81583	1.32E-99	1.26E-98
d:Bacteria(1.0000),p:Verrucomicrobia(0.8620),c:Subdivision5(0.6471),g:Subdivision5_genera_incertae_sedis(0.0409);	11.88808	2.14E-99	2.00E-98
d:Bacteria(1.0000),p:Bacteroidetes(0.9093),c:Bacteroidia(0.3967),o:Bacteroidales(0.1942),f:Bacteroidaceae(0.0296),g:Bacteroides(0.0094);	11.98445	2.77E-99	2.54E-98
d:Bacteria(1.0000),p:Bacteroidetes(0.8147),c:Bacteroidetes_incertae_sedis(0.0345),g:Ohtaekwangia(0.0043);	12.72747	1.66E-98	1.49E-97
d:Bacteria(1.0000),p:Firmicutes(0.2622),c:Erysipelotrichia(0.0345),o:Erysipelotrichales(0.0136),f:Erysipelotrichaceae(0.0061),g:Clostridium_XVIII(0.0043);	12.41949	1.51E-97	1.33E-96
d:Bacteria(1.0000),p:Firmicutes(0.1875),c:Erysipelotrichia(0.0315),o:Erysipelotrichales(0.0123),f:Erysipelotrichaceae(0.0042),g:Erysipelotrichaceae_incertae_sedis(0.0034);	12.52944	6.74E-94	5.83E-93
d:Bacteria(0.9753),p:Bacteroidetes(0.7674),c:Sphingobacteriia(0.0345),o:Sphingobacteriales(0.0136),f:Sphingobacteriaceae(0.0061),g:Mucilaginibacter(0.0031);	12.61559	8.86E-93	7.51E-92
d:Bacteria(1.0000),p:Spirochaetes(0.9970),c:Spirochaetia(0.9449),o:Spirochaetales(0.8617),f:Spirochaetaceae(0.7676),g:Treponema(0.4282);	12.18603	5.05E-92	4.20E-91
d:Bacteria(1.0000),p:Proteobacteria(0.9914),c:Deltaproteobacteria(0.8562),o:Myxococcales(0.5540),f:Myxococcaceae(0.1311),g:Corallococcus(0.0215);	12.31509	1.14E-91	9.34E-91
d:Bacteria(1.0000),p:Bacteroidetes(0.9329),c:Sphingobacteriia(0.5845),o:Sphingobacteriales(0.3031),f:Sphingobacteriaceae(0.1525),g:Pedobacter(0.0143);	11.27762	3.29E-91	2.64E-90
d:Bacteria(1.0000),p:Firmicutes(0.5785),c:Clostridia(0.2725),o:Clostridiales(0.1218),f:Clostridiaceae_1(0.0218),g:Clostridium_sensu_stricto(0.0077);	12.94301	1.91E-90	1.51E-89
d:Bacteria(1.0000),p:Bacteroidetes(0.8147),c:Sphingobacteriia(0.0345),o:Sphingobacteriales(0.0136),f:Sphingobacteriaceae(0.0061),g:Olivibacter(0.0043);	13.14258	5.62E-88	4.36E-87
d:Bacteria(1.0000),p:Bacteroidetes(0.8620),c:Sphingobacteriia(0.2113),o:Sphingobacteriales(0.0945),f:Sphingobacteriaceae(0.0296),g:Parapedobacter(0.0077);	12.23056	8.32E-86	6.35E-85
d:Archaea(0.9753),p:Euryarchaeota(0.8384),c:Thermoplasmata(0.1500),o:Methanomassiliicoccales(0.0673),f:Methanomassiliicoccaceae(0.0218),g:Methanomassiliicoccus(0.0077);	11.3936	2.86E-85	2.15E-84
d:Bacteria(0.9753),p:Bacteroidetes(0.7911),c:Bacteroidia(0.0285),o:Bacteroidales(0.0111),f:Porphyromonadaceae(0.0037),g:Dysgonomonas(0.0031);	11.80683	1.26E-84	9.31E-84
d:Bacteria(1.0000),p:Firmicutes(0.7674),c:Clostridia(0.3967),o:Clostridiales(0.1942),f:Syntrophomonadaceae(0.0883),g:Syntrophothermus(0.0167);	12.25434	2.32E-82	1.68E-81
d:Bacteria(1.0000),p:Verrucomicrobia(0.8384),c:Subdivision5(0.5845),g:Subdivision5_genera_incertae_sedis(0.0295);	10.87078	6.08E-82	4.35E-81
d:Bacteria(1.0000),p:Firmicutes(0.9946),c:Clostridia(0.9207),o:Clostridiales(0.7746),f:Ruminococcaceae(0.4677),g:Intestinimonas(0.0865);	12.22323	1.81E-81	1.27E-80
d:Bacteria(1.0000),p:Firmicutes(0.6840),c:Clostridia(0.3342),o:Clostridiales(0.1579),f:Clostridiaceae_2(0.0218),g:Alkaliphilus(0.0077);	12.77802	1.40E-79	9.71E-79
d:Bacteria(1.0000),p:Bacteroidetes(0.9802),c:Sphingobacteriia(0.3967),o:Sphingobacteriales(0.1942),f:Sphingobacteriaceae(0.0883),g:Pedobacter(0.0143);	11.51544	3.50E-77	2.39E-76
d:Bacteria(1.0000),p:Proteobacteria(0.9996),c:Epsilonproteobacteria(0.9974),o:Campylobacterales(0.9862),f:Helicobacteraceae(0.9604),g:Helicobacter(0.7814);	11.47127	5.65E-77	3.81E-76
d:Bacteria(1.0000),p:Verrucomicrobia(0.8620),c:Subdivision5(0.6471),g:Subdivision5_genera_incertae_sedis(0.0409);	9.614384	8.93E-75	5.93E-74
d:Bacteria(1.0000),p:Firmicutes(0.4731),c:Erysipelotrichia(0.2113),o:Erysipelotrichales(0.0945),f:Erysipelotrichaceae(0.0296),g:Clostridium_XVIII(0.0094);	10.72126	3.49E-72	2.29E-71
d:Bacteria(1.0000),p:Spirochaetes(0.9991),c:Spirochaetia(0.9668),o:Spirochaetales(0.9457),f:Spirochaetaceae(0.9026),g:Treponema(0.7814);	13.44942	1.08E-69	6.96E-69
d:Bacteria(1.0000),p:Bacteroidetes(0.9093),c:Bacteroidia(0.3342),o:Bacteroidales(0.1579),f:Porphyromonadaceae(0.0668),g:Paludibacter(0.0119);	13.59139	3.06E-69	1.95E-68
d:Bacteria(1.0000),p:Tenericutes(0.7674),c:Mollicutes(0.3967),o:Acholeplasmatales(0.1942),f:Acholeplasmataceae(0.0883),g:Acholeplasma(0.0167);	12.34056	1.18E-68	7.41E-68
d:Bacteria(1.0000),p:Bacteroidetes(0.9988),c:Bacteroidia(0.9023),o:Bacteroidales(0.7746),f:Prevotellaceae(0.2665),g:Prevotella(0.0637);	12.79437	2.12E-66	1.32E-65
d:Bacteria(1.0000),p:Firmicutes(0.9988),c:Clostridia(0.9631),o:Clostridiales(0.9317),f:Lachnospiraceae(0.6553),g:Ruminococcus2(0.3238);	11.0091	7.27E-62	4.44E-61
d:Bacteria(1.0000),p:Firmicutes(0.7674),c:Erysipelotrichia(0.3967),o:Erysipelotrichales(0.1942),f:Erysipelotrichaceae(0.0883),g:Clostridium_XVIII(0.0167);	11.78532	2.13E-60	1.28E-59
d:Bacteria(1.0000),p:Bacteroidetes(0.9899),c:Sphingobacteriia(0.5219),o:Sphingobacteriales(0.2668),f:Sphingobacteriaceae(0.1311),g:Parapedobacter(0.0143);	12.21212	2.76E-52	1.64E-51
d:Bacteria(1.0000),p:Proteobacteria(0.7674),c:Alphaproteobacteria(0.2725),o:Kiloniellales(0.0400),f:Kiloniellaceae(0.0140),g:Kiloniella(0.0060);	10.58261	2.21E-44	1.30E-43
d:Bacteria(1.0000),p:Firmicutes(0.9989),c:Clostridia(0.9391),o:Clostridiales(0.8477),f:Lachnospiraceae(0.6553),g:Clostridium_XlVa(0.4282);	12.80356	1.02E-43	5.94E-43
d:Bacteria(1.0000),p:Firmicutes(0.9566),c:Clostridia(0.8470),o:Clostridiales(0.4658),f:Ruminococcaceae(0.1861),g:Clostridium_IV(0.0167);	5.927691	5.09E-30	2.91E-29
d:Bacteria(1.0000),p:Firmicutes(0.9999),c:Clostridia(0.9937),o:Clostridiales(0.9926),f:Clostridiaceae_1(0.9701),g:Clostridium_sensu_stricto(0.9701);	6.139891	9.21E-30	5.21E-29
d:Bacteria(1.0000),p:Firmicutes(0.9991),c:Clostridia(0.9023),o:Clostridiales(0.7746),f:Lachnospiraceae(0.5804),g:Clostridium_XlVa(0.2194);	-9.55277	4.53E-29	2.53E-28
d:Bacteria(1.0000),p:Firmicutes(0.8857),c:Clostridia(0.7097),o:Clostridiales(0.3031),f:Ruminococcaceae(0.1097),g:Intestinimonas(0.0119);	5.241771	9.03E-27	4.98E-26
d:Bacteria(1.0000),p:Firmicutes(0.9987),c:Clostridia(0.9960),o:Clostridiales(0.9960),f:Peptostreptococcaceae(0.9786),g:Clostridium_XI(0.9786);	5.442596	6.49E-24	3.54E-23
d:Bacteria(1.0000),p:Tenericutes(0.8147),c:Mollicutes(0.5219),o:Acholeplasmatales(0.2305),f:Acholeplasmataceae(0.1097),g:Acholeplasma(0.0191);	6.112906	8.42E-24	4.54E-23
d:Bacteria(1.0000),p:Bacteroidetes(0.9990),c:Bacteroidia(0.9973),o:Bacteroidales(0.9973),f:Prevotellaceae(0.9191),g:Prevotella(0.8158);	-10.7096	1.42E-22	7.59E-22
d:Bacteria(0.9753),p:Bacteroidetes(0.5785),c:Sphingobacteriia(0.0345),o:Sphingobacteriales(0.0136),f:Sphingobacteriaceae(0.0061),g:Pseudosphingobacterium(0.0031);	-11.7511	1.62E-22	8.55E-22
d:Bacteria(1.0000),p:Firmicutes(0.9986),c:Clostridia(0.9960),o:Clostridiales(0.9960),f:Ruminococcaceae(0.9914),g:Ruminococcus(0.9914);	-9.00146	1.72E-22	8.93E-22
d:Bacteria(1.0000),p:Firmicutes(0.9987),c:Clostridia(0.9922),o:Clostridiales(0.9778),f:Ruminococcaceae(0.9357),g:Ruminococcus(0.8846);	-10.7069	3.15E-22	1.62E-21
d:Bacteria(1.0000),p:Bacteroidetes(0.9988),c:Bacteroidia(0.9922),o:Bacteroidales(0.9778),f:Prevotellaceae(0.4677),g:Prevotella(0.0523);	-10.3357	5.92E-22	3.01E-21
d:Bacteria(1.0000),p:Proteobacteria(0.7674),c:Deltaproteobacteria(0.3967),o:Syntrophobacterales(0.0673),f:Syntrophaceae(0.0218),g:Desulfomonile(0.0077);	7.664169	7.72E-22	3.88E-21
d:Bacteria(1.0000),p:Bacteroidetes(0.9930),c:Bacteroidia(0.8654),o:Bacteroidales(0.5981),f:Prevotellaceae(0.2263),g:Paraprevotella(0.0523);	4.496664	1.15E-21	5.74E-21
d:Bacteria(1.0000),p:Bacteroidetes(0.9093),c:Bacteroidia(0.2725),o:Bacteroidales(0.1218),f:Marinilabiaceae(0.0454),g:Anaerophaga(0.0119);	-10.583	1.18E-21	5.76E-21
d:Bacteria(1.0000),p:Firmicutes(0.9999),c:Clostridia(0.9952),o:Clostridiales(0.9952),f:Lachnospiraceae(0.9357),g:Dorea(0.8846);	-6.34702	1.18E-21	5.76E-21
d:Bacteria(1.0000),p:Bacteroidetes(0.9093),c:Flavobacteriia(0.3342),o:Flavobacteriales(0.1579),f:Flavobacteriaceae(0.0668),g:Riemerella(0.0143);	-10.9406	1.61E-21	7.77E-21
d:Bacteria(1.0000),p:Firmicutes(0.9329),c:Clostridia(0.8348),o:Clostridiales(0.3775),f:Ruminococcaceae(0.1525),g:Clostridium_IV(0.0143);	-10.4483	4.12E-21	1.96E-20
d:Bacteria(1.0000),p:Firmicutes(0.9938),c:Clostridia(0.9023),o:Clostridiales(0.7305),f:Ruminococcaceae(0.2665),g:Papillibacter(0.0637);	4.506009	1.48E-20	6.98E-20

d:Bacteria(1.0000),p:Verrucomicrobia(0.9899),c:Subdivision5(0.8654),g:Subdivision5_genera_incertae_sedis(0.1672);	-10.4951	2.53E-20	1.18E-19
d:Bacteria(1.0000),p:Firmicutes(0.9995),c:Clostridia(0.9814),o:Clostridiales(0.9715),f:Lachnospiraceae(0.9026),g:Coprococcus(0.6782);	-6.29871	5.16E-20	2.39E-19
d:Bacteria(1.0000),p:Firmicutes(0.7674),c:Clostridia(0.2725),o:Clostridiales(0.1218),f:Lachnospiraceae(0.0454),g:Anaerostipes(0.0119);	4.45173	8.24E-20	3.78E-19
d:Bacteria(1.0000),p:Bacteroidetes(0.9987),c:Bacteroidia(0.9704),o:Bacteroidales(0.9597),f:Rikenellaceae(0.9109),g:Alistipes(0.7814);	-9.18769	8.77E-20	3.98E-19
d:Bacteria(1.0000),p:Firmicutes(0.9999),c:Clostridia(0.9954),o:Clostridiales(0.9954),f:Lachnospiraceae(0.9533),g:Ruminococcus2(0.9533);	-6.69952	1.04E-19	4.69E-19
d:Bacteria(1.0000),p:Bacteroidetes(0.9984),c:Bacteroidia(0.9947),o:Bacteroidales(0.9947),f:Prevotellaceae(0.7302),g:Prevotella(0.4282);	-11.1439	1.14E-19	5.06E-19
d:Bacteria(1.0000),p:Bacteroidetes(0.9899),c:Bacteroidia(0.8562),o:Bacteroidales(0.5540),f:Prevotellaceae(0.0883),g:Alloprevotella(0.0167);	-10.5748	1.26E-19	5.53E-19
d:Bacteria(1.0000),p:Firmicutes(0.8620),c:Clostridia(0.6471),o:Clostridiales(0.3394),f:Ruminococcaceae(0.1097),g:Ruminococcus(0.0143);	-11.1311	1.28E-19	5.57E-19
d:Bacteria(1.0000),p:Firmicutes(0.8620),c:Clostridia(0.3967),o:Clostridiales(0.1942),f:Clostridiaceae_4(0.0454),g:Thermotalea(0.0119);	6.079718	1.53E-19	6.59E-19
d:Bacteria(1.0000),p:Firmicutes(0.7911),c:Clostridia(0.4593),o:Clostridiales(0.2305),f:Clostridiaceae_4(0.0454),g:Thermotalea(0.0119);	-10.0496	1.67E-19	7.14E-19
d:Bacteria(1.0000),p:Bacteroidetes(0.9998),c:Bacteroidia(0.9989),o:Bacteroidales(0.9989),f:Porphyromonadaceae(0.9987),g:Butyricimonas(0.9987);	-10.9672	2.44E-19	1.03E-18
d:Bacteria(1.0000),p:Firmicutes(1.0000),c:Clostridia(0.9970),o:Clostridiales(0.9970),f:Lachnospiraceae(0.9878),g:Blautia(0.9743);	-4.14472	2.49E-19	1.05E-18
d:Bacteria(1.0000),p:Firmicutes(0.8620),c:Clostridia(0.6471),o:Clostridiales(0.2668),f:Ruminococcaceae(0.1311),g:Intestinimonas(0.0191);	-10.7361	2.76E-19	1.14E-18
d:Bacteria(1.0000),p:Firmicutes(0.7911),c:Erysipelotrichia(0.3967),o:Erysipelotrichales(0.1942),f:Erysipelotrichaceae(0.0883),g:Clostridium_XVIII(0.0167);	-10.2712	2.77E-19	1.14E-18
d:Bacteria(1.0000),p:Firmicutes(0.9993),c:Clostridia(0.9559),o:Clostridiales(0.9037),f:Ruminococcaceae(0.4275),g:Butyricicoccus(0.1672);	-10.6917	3.53E-19	1.44E-18
d:Bacteria(1.0000),p:Firmicutes(0.9970),c:Clostridia(0.9299),o:Clostridiales(0.8337),f:Ruminococcaceae(0.5430),g:Sporobacter(0.0865);	-8.5334	4.07E-19	1.65E-18
d:Bacteria(1.0000),p:Bacteroidetes(0.8857),c:Bacteroidia(0.2113),o:Bacteroidales(0.0945),f:Porphyromonadaceae(0.0296),g:Barnesiella(0.0094);	-10.8896	4.18E-19	1.68E-18
d:Bacteria(1.0000),p:Firmicutes(0.8147),c:Clostridia(0.5219),o:Clostridiales(0.2305),f:Ruminococcaceae(0.0454),g:Intestinimonas(0.0094);	5.685682	7.58E-19	3.02E-18
d:Bacteria(1.0000),p:Bacteroidetes(0.9989),c:Bacteroidia(0.8470),o:Bacteroidales(0.5099),f:Porphyromonadaceae(0.3470),g:Barnesiella(0.0523);	-10.7575	1.08E-18	4.28E-18
d:Bacteria(1.0000),p:Firmicutes(0.9984),c:Clostridia(0.9814),o:Clostridiales(0.9715),f:Lachnospiraceae(0.9357),g:Lachnospiracea_incertae_sedis(0.9189);	-4.93452	1.13E-18	4.42E-18
d:Bacteria(1.0000),p:Fibrobacteres(0.9983),c:Fibrobacteria(0.9957),o:Fibrobacterales(0.9957),f:Fibrobacteraceae(0.9957),g:Fibrobacter(0.9957);	-9.47727	1.44E-18	5.61E-18
d:Bacteria(1.0000),p:Spirochaetes(0.9988),c:Spirochaetia(0.9886),o:Spirochaetales(0.9757),f:Spirochaetaceae(0.9522),g:Treponema(0.7814);	-11.597	2.47E-18	9.51E-18
d:Bacteria(1.0000),p:Firmicutes(0.8147),c:Clostridia(0.5219),o:Clostridiales(0.1942),f:Ruminococcaceae(0.0883),g:Clostridium_III(0.0119);	3.839097	2.81E-18	1.07E-17
d:Bacteria(1.0000),p:Firmicutes(1.0000),c:Clostridia(0.9952),o:Clostridiales(0.9952),f:Lachnospiraceae(0.9837),g:Dorea(0.9701);	-5.96639	3.35E-18	1.27E-17
d:Bacteria(1.0000),p:Firmicutes(0.9938),c:Clostridia(0.8654),o:Clostridiales(0.5981),f:Lachnospiraceae(0.3068),g:Cellulosilyticum(0.0751);	6.23064	3.46E-18	1.30E-17
d:Bacteria(1.0000),p:Firmicutes(0.9991),c:Clostridia(0.9559),o:Clostridiales(0.9037),f:Lachnospiraceae(0.6928),g:Clostridium_XlVa(0.3760);	-4.25622	4.51E-18	1.68E-17
d:Bacteria(1.0000),p:Bacteroidetes(0.9993),c:Bacteroidia(0.9979),o:Bacteroidales(0.9979),f:Prevotellaceae(0.9522),g:Prevotella(0.9189);	-13.3458	4.69E-18	1.73E-17
d:Bacteria(1.0000),p:Firmicutes(1.0000),c:Clostridia(0.9960),o:Clostridiales(0.9960),f:Lachnospiraceae(0.9770),g:Blautia(0.9189);	-5.33815	7.26E-18	2.66E-17
d:Bacteria(1.0000),p:Firmicutes(0.9983),c:Clostridia(0.9924),o:Clostridiales(0.9799),f:Lachnospiraceae(0.9191),g:Anaerostipes(0.8846);	-5.0524	9.16E-18	3.33E-17
d:Bacteria(1.0000),p:Verrucomicrobia(0.9329),c:Subdivision5(0.8348),g:Subdivision5_genera_incertae_sedis(0.0751);	-11.5482	1.06E-17	3.82E-17
d:Bacteria(1.0000),p:Actinobacteria(0.9899),c:Actinobacteria(0.7097),o:Coriobacteriales(0.3775),f:Coriobacteriaceae(0.2263),g:Olsenella(0.0191);	-10.7938	1.23E-17	4.39E-17
d:Bacteria(1.0000),p:Bacteroidetes(0.9954),c:Bacteroidia(0.8654),o:Bacteroidales(0.5981),f:Porphyromonadaceae(0.4275),g:Parabacteroides(0.0409);	-10.905	1.68E-17	5.95E-17
d:Bacteria(1.0000),p:Firmicutes(0.9992),c:Erysipelotrichia(0.9595),o:Erysipelotrichales(0.9177),f:Erysipelotrichaceae(0.8861),g:Clostridium_XVIII(0.3760);	-4.99963	2.32E-17	8.18E-17
d:Bacteria(1.0000),p:Verrucomicrobia(0.8620),c:Subdivision5(0.6471),g:Subdivision5_genera_incertae_sedis(0.0409);	-10.0861	2.95E-17	1.03E-16
d:Bacteria(1.0000),p:Firmicutes(0.9329),c:Clostridia(0.8348),o:Clostridiales(0.3031),f:Ruminococcaceae(0.1525),g:Intestinimonas(0.0191);	-7.95482	3.03E-17	1.05E-16
d:Bacteria(1.0000),p:Bacteroidetes(0.9990),c:Bacteroidia(0.9970),o:Bacteroidales(0.9970),f:Prevotellaceae(0.9109),g:Prevotella(0.8158);	-8.72731	3.40E-17	1.17E-16
d:Bacteria(1.0000),p:Firmicutes(1.0000),c:Clostridia(0.9973),o:Clostridiales(0.9973),f:Lachnospiraceae(0.9888),g:Fusicatenibacter(0.9786);	-5.39776	3.50E-17	1.20E-16
d:Bacteria(1.0000),p:Firmicutes(0.8384),c:Clostridia(0.1500),o:Clostridiales(0.0673),f:Lachnospiraceae(0.0218),g:Clostridium_XlVa(0.0077);	5.268045	4.74E-17	1.61E-16
d:Bacteria(1.0000),p:Firmicutes(0.9093),c:Clostridia(0.7723),o:Clostridiales(0.3394),f:Ruminococcaceae(0.1311),g:Intestinimonas(0.0215);	4.609574	4.91E-17	1.66E-16
d:Bacteria(1.0000),p:Firmicutes(0.9996),c:Clostridia(0.9937),o:Clostridiales(0.9926),f:Ruminococcaceae(0.9878),g:Faecalibacterium(0.7126);	-5.12091	6.02E-17	2.01E-16
d:Bacteria(1.0000),p:Firmicutes(0.9986),c:Clostridia(0.9937),o:Clostridiales(0.9926),f:Lachnospiraceae(0.9687),g:Roseburia(0.6095);	-4.74515	1.08E-16	3.58E-16
d:Bacteria(1.0000),p:Firmicutes(0.9329),c:Clostridia(0.7097),o:Clostridiales(0.2668),f:Ruminococcaceae(0.1097),g:Intestinimonas(0.0191);	-10.9766	1.44E-16	4.74E-16
d:Bacteria(1.0000),p:Bacteroidetes(0.9988),c:Bacteroidia(0.9850),o:Bacteroidales(0.9736),f:Prevotellaceae(0.8861),g:Alloprevotella(0.6782);	-9.18301	1.86E-16	6.08E-16
d:Bacteria(1.0000),p:Firmicutes(0.5785),c:Clostridia(0.2725),o:Clostridiales(0.1218),f:Ruminococcaceae(0.0454),g:Ruminococcus(0.0119);	5.29272	2.09E-16	6.78E-16
d:Bacteria(1.0000),p:Firmicutes(0.9989),c:Clostridia(0.9391),o:Clostridiales(0.8477),f:Lachnospiraceae(0.5804),g:Blautia(0.3238);	-4.47553	2.17E-16	7.00E-16
d:Bacteria(1.0000),p:Firmicutes(0.7674),c:Clostridia(0.3967),o:Clostridiales(0.1942),f:Lachnospiraceae(0.0454),g:Coprococcus(0.0119);	-8.28135	2.22E-16	7.13E-16
d:Bacteria(1.0000),p:Firmicutes(1.0000),c:Bacilli(0.9926),o:Lactobacillales(0.9820),f:Lactobacillaceae(0.9786),g:Lactobacillus(0.9786);	-4.334	3.30E-16	1.05E-15
d:Bacteria(1.0000),p:Firmicutes(0.9996),c:Clostridia(0.9741),o:Clostridiales(0.9672),f:Lachnospiraceae(0.8778),g:Clostridium_XlVa(0.7126);	-4.18782	3.43E-16	1.08E-15
d:Bacteria(1.0000),p:Bacteroidetes(0.9946),c:Bacteroidia(0.9207),o:Bacteroidales(0.8197),f:Prevotellaceae(0.3872),g:Alloprevotella(0.0865);	-10.9382	5.35E-16	1.68E-15
d:Bacteria(1.0000),p:Spirochaetes(0.9970),c:Spirochaetia(0.9449),o:Spirochaetales(0.8617),f:Spirochaetaceae(0.7676),g:Treponema(0.3760);	-8.55874	6.01E-16	1.87E-15

d:Bacteria(1.0000),p:Firmicutes(0.9566),c:Clostridia(0.8470),o:Clostridiales(0.5099),f:Clostridiaceae_1(0.1861),g:Caloramator(0.0409);	-7.40225	7.40E-16	2.29E-15
d:Bacteria(1.0000),p:Bacteroidetes(0.9907),c:Bacteroidia(0.5845),o:Bacteroidales(0.3031),f:Porphyromonadaceae(0.1525),g:Paludibacter(0.0295);	-8.49587	7.83E-16	2.41E-15
d:Bacteria(1.0000),p:Firmicutes(0.9989),c:Clostridia(0.9970),o:Clostridiales(0.9970),f:Ruminococcaceae(0.9970),g:Gemmiger(0.7126);	-6.05057	7.91E-16	2.42E-15
d:Bacteria(1.0000),p:Tenericutes(0.8620),c:Mollicutes(0.6471),o:Acholeplasmatales(0.3394),f:Acholeplasmataceae(0.1861),g:Acholeplasma(0.0409);	6.135686	8.35E-16	2.53E-15
d:Bacteria(1.0000),p:Tenericutes(0.4731),c:Mollicutes(0.2113),o:Anaeroplasmatales(0.0945),f:Anaeroplasmataceae(0.0296),g:Asteroleplasma(0.0094);	-8.98176	1.26E-15	3.78E-15
d:Bacteria(1.0000),p:Firmicutes(0.4731),c:Clostridia(0.2113),o:Clostridiales(0.0945),f:Clostridiaceae_3(0.0218),g:Brassicibacter(0.0077);	-8.18221	1.83E-15	5.49E-15
d:Bacteria(1.0000),p:Firmicutes(0.9994),c:Clostridia(0.9983),o:Clostridiales(0.9983),f:Ruminococcaceae(0.9847),g:Faecalibacterium(0.6782);	-5.10745	1.85E-15	5.51E-15
d:Bacteria(1.0000),p:Firmicutes(0.9998),c:Clostridia(0.9956),o:Clostridiales(0.9956),f:Ruminococcaceae(0.9898),g:Faecalibacterium(0.8158);	-5.83372	5.39E-15	1.59E-14
d:Bacteria(1.0000),p:Bacteroidetes(0.9566),c:Flavobacteriia(0.5219),o:Flavobacteriales(0.2668),f:Flavobacteriaceae(0.1311),g:Galbibacter(0.0143);	-10.9826	6.18E-15	1.81E-14
d:Bacteria(1.0000),p:Firmicutes(0.2622),c:Erysipelotrichia(0.0888),o:Erysipelotrichales(0.0400),f:Erysipelotrichaceae(0.0140),g:Clostridium_XVIII(0.0060);	-9.66061	7.76E-15	2.26E-14
d:Bacteria(1.0000),p:Firmicutes(0.9993),c:Clostridia(0.9299),o:Clostridiales(0.8337),f:Lachnospiraceae(0.5055),g:Clostridium_XlVa(0.0865);	-4.6892	8.04E-15	2.33E-14
d:Bacteria(1.0000),p:Firmicutes(0.9946),c:Clostridia(0.5845),o:Clostridiales(0.3031),f:Clostridiaceae_1(0.1525),g:Anaerosporobacter(0.0295);	-4.098	2.82E-14	8.12E-14
d:Bacteria(1.0000),p:Firmicutes(0.9987),c:Clostridia(0.9935),o:Clostridiales(0.9904),f:Lachnospiraceae(0.9837),g:Lachnospiracea_incertae_sedis(0.9765);	-4.86016	2.87E-14	8.23E-14
d:Bacteria(1.0000),p:Tenericutes(0.1875),c:Mollicutes(0.0345),o:Entomoplasmatales(0.0136),f:Entomoplasmataceae(0.0061),g:Entomoplasma(0.0043);	-12.9507	3.47E-14	9.87E-14
d:Bacteria(1.0000),p:Firmicutes(1.0000),c:Clostridia(0.9968),o:Clostridiales(0.9968),f:Lachnospiraceae(0.9770),g:Blautia(0.9743);	-3.56268	4.12E-14	1.17E-13
d:Bacteria(1.0000),p:Firmicutes(0.9990),c:Clostridia(0.9207),o:Clostridiales(0.8197),f:Lachnospiraceae(0.6179),g:Lachnospiracea_incertae_sedis(0.2194);	-4.90031	5.12E-14	1.44E-13
d:Bacteria(1.0000),p:Bacteroidetes(0.9802),c:Bacteroidia(0.5219),o:Bacteroidales(0.2668),f:Porphyromonadaceae(0.1311),g:Paludibacter(0.0191);	-6.20823	5.60E-14	1.56E-13
d:Bacteria(1.0000),p:Bacteroidetes(0.9985),c:Bacteroidia(0.9935),o:Bacteroidales(0.9904),f:Prevotellaceae(0.6928),g:Prevotella(0.4804);	-10.1886	6.42E-14	1.78E-13
d:Bacteria(1.0000),p:Firmicutes(0.9995),c:Clostridia(0.9924),o:Clostridiales(0.9799),f:Lachnospiraceae(0.9191),g:Roseburia(0.5327);	-4.75231	7.75E-14	2.14E-13
d:Bacteria(1.0000),p:Firmicutes(1.0000),c:Clostridia(0.9960),o:Clostridiales(0.9960),f:Lachnospiraceae(0.9888),g:Roseburia(0.9786);	-4.105	8.55E-14	2.35E-13
d:Bacteria(1.0000),p:Bacteroidetes(0.9994),c:Bacteroidia(0.9937),o:Bacteroidales(0.9926),f:Prevotellaceae(0.9533),g:Prevotella(0.9533);	-5.69268	8.82E-14	2.40E-13
d:Bacteria(1.0000),p:Firmicutes(0.9994),c:Clostridia(0.9704),o:Clostridiales(0.9597),f:Lachnospiraceae(0.8051),g:Clostridium_XlVa(0.6095);	-3.95682	1.43E-13	3.87E-13
d:Bacteria(1.0000),p:Firmicutes(0.9995),c:Clostridia(0.9926),o:Clostridiales(0.9820),f:Lachnospiraceae(0.8943),g:Blautia(0.6095);	-4.03792	1.45E-13	3.91E-13
d:Bacteria(1.0000),p:Bacteroidetes(0.9907),c:Bacteroidia(0.4593),o:Bacteroidales(0.2305),f:Porphyromonadaceae(0.1097),g:Paludibacter(0.0167);	-7.08379	1.54E-13	4.12E-13
d:Bacteria(1.0000),p:Firmicutes(0.9987),c:Clostridia(0.9956),o:Clostridiales(0.9956),f:Lachnospiraceae(0.9604),g:Blautia(0.9533);	-5.24487	2.14E-13	5.71E-13
d:Bacteria(1.0000),p:Bacteroidetes(0.8147),c:Sphingobacteriia(0.0345),o:Sphingobacteriales(0.0136),f:Sphingobacteriaceae(0.0061),g:Parapedobacter(0.0043);	-11.4078	3.10E-13	8.20E-13
d:Bacteria(1.0000),p:Tenericutes(0.8147),c:Mollicutes(0.5219),o:Acholeplasmatales(0.2668),f:Acholeplasmataceae(0.1311),g:Acholeplasma(0.0215);	5.253944	4.57E-13	1.20E-12
d:Bacteria(1.0000),p:Firmicutes(0.6840),c:Clostridia(0.3342),o:Clostridiales(0.1579),f:Clostridiaceae_4(0.0296),g:Thermotalea(0.0094);	4.201763	8.18E-13	2.14E-12
d:Bacteria(1.0000),p:Tenericutes(0.4731),c:Mollicutes(0.2113),o:Acholeplasmatales(0.0945),f:Acholeplasmataceae(0.0296),g:Acholeplasma(0.0094);	4.474011	8.61E-13	2.24E-12
d:Bacteria(1.0000),p:Firmicutes(0.9991),c:Clostridia(0.9449),o:Clostridiales(0.8617),f:Eubacteriaceae(0.5804),g:Eubacterium(0.3760);	3.620024	9.01E-13	2.33E-12
d:Bacteria(1.0000),p:Firmicutes(0.9984),c:Clostridia(0.9814),o:Clostridiales(0.9715),f:Lachnospiraceae(0.8943),g:Lachnospiracea_incertae_sedis(0.6438);	-5.02769	9.70E-13	2.50E-12
d:Bacteria(1.0000),p:Bacteroidetes(0.9914),c:Bacteroidia(0.8348),o:Bacteroidales(0.4658),f:Porphyromonadaceae(0.3068),g:Barnesiella(0.0215);	-9.78713	9.96E-13	2.55E-12
d:Bacteria(1.0000),p:Firmicutes(0.9989),c:Clostridia(0.9777),o:Clostridiales(0.9693),f:Ruminococcaceae(0.8943),g:Anaerotruncus(0.5327);	3.706616	1.53E-12	3.88E-12
d:Bacteria(1.0000),p:Bacteroidetes(0.9329),c:Bacteroidia(0.8348),o:Bacteroidales(0.4658),f:Prevotellaceae(0.1097),g:Alloprevotella(0.0143);	-8.48614	1.59E-12	4.02E-12
d:Bacteria(1.0000),p:Bacteroidetes(1.0000),c:Bacteroidia(1.0000),o:Bacteroidales(1.0000),f:Bacteroidaceae(0.9871),g:Bacteroides(0.9871);	-6.1651	2.11E-12	5.32E-12
d:Bacteria(1.0000),p:Bacteroidetes(0.9993),c:Bacteroidia(0.9979),o:Bacteroidales(0.9979),f:Prevotellaceae(0.9687),g:Prevotella(0.9533);	-9.45428	2.66E-12	6.67E-12
d:Bacteria(1.0000),p:Bacteroidetes(0.8384),c:Sphingobacteriia(0.2113),o:Sphingobacteriales(0.0945),f:Sphingobacteriaceae(0.0296),g:Pedobacter(0.0094);	-11.0814	2.68E-12	6.68E-12
d:Bacteria(1.0000),p:Bacteroidetes(0.8147),c:Bacteroidia(0.0345),o:Bacteroidales(0.0136),f:Rikenellaceae(0.0061),g:Alistipes(0.0043);	3.881188	2.94E-12	7.28E-12
d:Bacteria(1.0000),p:Actinobacteria(0.9985),c:Actinobacteria(0.9954),o:Coriobacteriales(0.9954),f:Coriobacteriaceae(0.9908),g:Collinsella(0.9722);	-5.1232	4.94E-12	1.22E-11
d:Bacteria(1.0000),p:Tenericutes(0.2622),c:Mollicutes(0.0888),o:Anaeroplasmatales(0.0400),f:Anaeroplasmataceae(0.0140),g:Asteroleplasma(0.0060);	-11.6086	6.45E-12	1.58E-11
d:Bacteria(1.0000),p:Proteobacteria(0.7674),c:Alphaproteobacteria(0.3967),o:Rhodospirillales(0.0945),f:Rhodospirillaceae(0.0296),g:Fodinicurvata(0.0043);	-8.12789	6.96E-12	1.70E-11
d:Bacteria(1.0000),p:Proteobacteria(1.0000),c:Gammaproteobacteria(0.9952),o:Enterobacteriales(0.9841),f:Enterobacteriaceae(0.9837),g:Escherichia/Shigella(0.8502);	-5.7595	8.65E-12	2.10E-11
d:Bacteria(1.0000),p:Firmicutes(0.9998),c:Clostridia(0.9922),o:Clostridiales(0.9778),f:Lachnospiraceae(0.8943),g:Clostridium_XlVa(0.6095);	-4.41345	1.06E-11	2.55E-11
d:Bacteria(1.0000),p:Bacteroidetes(0.9930),c:Bacteroidia(0.8348),o:Bacteroidales(0.4658),f:Porphyromonadaceae(0.3068),g:Parabacteroides(0.0215);	-9.27241	1.36E-11	3.27E-11
d:Bacteria(1.0000),p:Firmicutes(0.9986),c:Clostridia(0.9777),o:Clostridiales(0.9693),f:Lachnospiraceae(0.8425),g:Lachnospiracea_incertae_sedis(0.5751);	-3.73511	1.49E-11	3.54E-11
d:Bacteria(1.0000),p:Firmicutes(0.9989),c:Clostridia(0.9559),o:Clostridiales(0.9037),f:Lachnospiraceae(0.7302),g:Clostridium_XlVa(0.5751);	-5.88158	1.93E-11	4.59E-11
d:Bacteria(1.0000),p:Firmicutes(0.8857),c:Clostridia(0.5219),o:Clostridiales(0.2668),f:Lachnospiraceae(0.0883),g:Clostridium_XlVb(0.0167);	-6.00652	2.57E-11	6.07E-11
d:Bacteria(1.0000),p:Firmicutes(0.9938),c:Clostridia(0.8931),o:Clostridiales(0.7305),f:Ruminococcaceae(0.5055),g:Sporobacter(0.0751);	-5.1852	2.66E-11	6.25E-11
d:Bacteria(1.0000),p:Tenericutes(0.2622),c:Mollicutes(0.0888),o:Entomoplasmatales(0.0400),f:Spiroplasmataceae(0.0140),g:Spiroplasma(0.0060);	-4.73568	3.77E-11	8.82E-11
d:Bacteria(1.0000),p:Firmicutes(0.9985),c:Negativicutes(0.9922),o:Selenomonadales(0.9778),f:Acidaminococcaceae(0.9274),g:Phascolarctobacterium(0.5751);	-4.83576	4.14E-11	9.62E-11

d:Bacteria(1.0000),p:Firmicutes(0.9946),c:Clostridia(0.8348),o:Clostridiales(0.4658),f:Clostridiaceae_1(0.2263),g:Anaerosporobacter(0.0523);	-5.99969	6.99E-11	1.62E-10
d:Bacteria(1.0000),p:Firmicutes(0.9988),c:Clostridia(0.9814),o:Clostridiales(0.9715),f:Ruminococcaceae(0.9274),g:Faecalibacterium(0.4804);	-3.47149	7.25E-11	1.67E-10
d:Bacteria(1.0000),p:Bacteroidetes(0.9914),c:Bacteroidia(0.7097),o:Bacteroidales(0.3775),f:Porphyromonadaceae(0.2263),g:Barnesiella(0.0215);	-10.5162	9.40E-11	2.15E-10
d:Bacteria(0.9753),p:Firmicutes(0.2622),c:Erysipelotrichia(0.0315),o:Erysipelotrichales(0.0123),f:Erysipelotrichaceae(0.0042),g:Erysipelothrix(0.0034);	-6.26591	1.18E-10	2.69E-10
d:Bacteria(1.0000),p:Firmicutes(0.9989),c:Clostridia(0.9486),o:Clostridiales(0.8757),f:Lachnospiraceae(0.8051),g:Lachnospiracea_incertae_sedis(0.5751);	-4.31534	1.30E-10	2.96E-10
d:Bacteria(1.0000),p:Bacteroidetes(0.9899),c:Sphingobacteriia(0.3342),o:Sphingobacteriales(0.1579),f:Sphingobacteriaceae(0.0668),g:Mucilaginibacter(0.0143);	-4.93255	1.34E-10	3.02E-10
d:Bacteria(1.0000),p:Firmicutes(0.9999),c:Clostridia(0.9994),o:Clostridiales(0.9994),f:Ruminococcaceae(0.9992),g:Subdoligranulum(0.6438);	-3.80234	1.51E-10	3.40E-10
d:Bacteria(1.0000),p:Bacteroidetes(0.9985),c:Bacteroidia(0.9933),o:Bacteroidales(0.9883),f:Prevotellaceae(0.7676),g:Prevotella(0.3238);	-10.7382	1.61E-10	3.59E-10
d:Bacteria(1.0000),p:Bacteroidetes(1.0000),c:Bacteroidia(0.9974),o:Bacteroidales(0.9974),f:Porphyromonadaceae(0.9974),g:Parabacteroides(0.9935);	-4.75454	1.86E-10	4.13E-10
d:Bacteria(1.0000),p:Actinobacteria(0.9914),c:Actinobacteria(0.8562),o:Coriobacteriales(0.5540),f:Coriobacteriaceae(0.3872),g:Slackia(0.0295);	-8.63467	1.87E-10	4.15E-10
d:Bacteria(1.0000),p:Actinobacteria(0.9938),c:Actinobacteria(0.8931),o:Coriobacteriales(0.7305),f:Coriobacteriaceae(0.5430),g:Slackia(0.0751);	-5.31416	3.34E-10	7.36E-10
d:Bacteria(1.0000),p:Firmicutes(0.5785),c:Erysipelotrichia(0.2725),o:Erysipelotrichales(0.1218),f:Erysipelotrichaceae(0.0454),g:Clostridium_XVIII(0.0119);	4.449491	3.42E-10	7.50E-10
d:Bacteria(1.0000),p:Firmicutes(0.9946),c:Clostridia(0.9023),o:Clostridiales(0.7746),f:Ruminococcaceae(0.3470),g:Ruminococcus(0.0523);	-4.55877	5.42E-10	1.18E-09
d:Bacteria(1.0000),p:Firmicutes(0.9987),c:Clostridia(0.9924),o:Clostridiales(0.9799),f:Ruminococcaceae(0.9687),g:Gemmiger(0.3238);	-6.81009	7.63E-10	1.66E-09
d:Bacteria(1.0000),p:Firmicutes(0.9992),c:Clostridia(0.9299),o:Clostridiales(0.8337),f:Ruminococcaceae(0.6928),g:Clostridium_IV(0.4282);	-3.25158	9.32E-10	2.01E-09
d:Bacteria(1.0000),p:Firmicutes(0.2622),c:Clostridia(0.0888),o:Clostridiales(0.0400),f:Clostridiaceae_3(0.0140),g:Brassicibacter(0.0060);	-4.57227	1.21E-09	2.61E-09
d:Bacteria(1.0000),p:Firmicutes(0.9988),c:Clostridia(0.9631),o:Clostridiales(0.9317),f:Lachnospiraceae(0.6928),g:Clostridium_XlVa(0.3238);	-4.44283	1.49E-09	3.19E-09
d:Bacteria(1.0000),p:Firmicutes(0.9986),c:Clostridia(0.9850),o:Clostridiales(0.9736),f:Lachnospiraceae(0.8051),g:Roseburia(0.5751);	-3.28137	1.61E-09	3.43E-09
d:Bacteria(1.0000),p:Spirochaetes(0.9999),c:Spirochaetia(0.9991),o:Spirochaetales(0.9991),f:Spirochaetaceae(0.9989),g:Treponema(0.9969);	-10.9972	1.96E-09	4.16E-09
d:Bacteria(1.0000),p:Firmicutes(0.9988),c:Clostridia(0.9886),o:Clostridiales(0.9757),f:Eubacteriaceae(0.8778),g:Eubacterium(0.7126);	-4.1007	2.03E-09	4.29E-09
d:Bacteria(1.0000),p:Bacteroidetes(0.9994),c:Bacteroidia(0.9981),o:Bacteroidales(0.9981),f:Prevotellaceae(0.9837),g:Prevotella(0.9189);	3.025453	2.27E-09	4.77E-09
d:Bacteria(1.0000),p:Firmicutes(0.9990),c:Clostridia(0.9559),o:Clostridiales(0.9037),f:Lachnospiraceae(0.5804),g:Roseburia(0.2716);	-4.13389	2.89E-09	6.04E-09
d:Bacteria(1.0000),p:Firmicutes(0.9990),c:Erysipelotrichia(0.9973),o:Erysipelotrichales(0.9973),f:Erysipelotrichaceae(0.9973),g:Clostridium_XVIII(0.9967);	-6.83946	3.20E-09	6.67E-09
d:Bacteria(1.0000),p:Firmicutes(0.9566),c:Clostridia(0.7097),o:Clostridiales(0.3775),f:Clostridiaceae_1(0.1097),g:Clostridium_sensu_stricto(0.0191);	-4.1725	3.31E-09	6.86E-09
d:Bacteria(1.0000),p:Firmicutes(0.9991),c:Clostridia(0.9975),o:Clostridiales(0.9975),f:Ruminococcaceae(0.9973),g:Faecalibacterium(0.9829);	-2.49576	4.19E-09	8.65E-09
d:Bacteria(1.0000),p:Bacteroidetes(0.9914),c:Bacteroidia(0.5845),o:Bacteroidales(0.3031),f:Porphyromonadaceae(0.1525),g:Paludibacter(0.0191);	2.923277	4.70E-09	9.66E-09
d:Bacteria(1.0000),p:Firmicutes(0.9992),c:Clostridia(0.9522),o:Clostridiales(0.8897),f:Ruminococcaceae(0.6553),g:Sporobacter(0.2194);	-3.88604	4.97E-09	1.02E-08
d:Bacteria(1.0000),p:Firmicutes(0.9962),c:Clostridia(0.9207),o:Clostridiales(0.8197),f:Clostridiales_Incertae_Sedis_XIII(0.3470),g:Anaerovorax(0.0865);	4.050097	5.28E-09	1.07E-08
d:Bacteria(1.0000),p:Proteobacteria(0.9987),c:Betaproteobacteria(0.9115),o:Rhodocyclales(0.5981),f:Rhodocyclaceae(0.4275),g:Denitratisoma(0.1672);	-5.23157	6.35E-09	1.29E-08
d:Bacteria(1.0000),p:Firmicutes(0.9986),c:Clostridia(0.9115),o:Clostridiales(0.8057),f:Lachnospiraceae(0.5055),g:Clostridium_XlVa(0.2716);	-3.54547	6.86E-09	1.38E-08
d:Bacteria(1.0000),p:Bacteroidetes(0.9986),c:Bacteroidia(0.9960),o:Bacteroidales(0.9960),f:Prevotellaceae(0.8943),g:Prevotella(0.4804);	3.677236	7.37E-09	1.48E-08
d:Bacteria(1.0000),p:Firmicutes(0.9986),c:Clostridia(0.9931),o:Clostridiales(0.9841),f:Ruminococcaceae(0.9687),g:Gemmiger(0.4282);	-5.72068	7.76E-09	1.55E-08
d:Bacteria(1.0000),p:Firmicutes(0.9983),c:Clostridia(0.9933),o:Clostridiales(0.9883),f:Lachnospiraceae(0.9770),g:Coprococcus(0.9701);	-4.38335	8.43E-09	1.68E-08
d:Bacteria(1.0000),p:Firmicutes(0.9998),c:Clostridia(0.9559),o:Clostridiales(0.9037),f:Lachnospiraceae(0.8778),g:Lachnospiracea_incertae_sedis(0.7126);	-2.79764	1.02E-08	2.01E-08
d:Bacteria(1.0000),p:Firmicutes(0.9962),c:Clostridia(0.9023),o:Clostridiales(0.7746),f:Ruminococcaceae(0.4275),g:Sporobacter(0.0751);	3.127693	1.24E-08	2.44E-08
d:Bacteria(1.0000),p:Firmicutes(0.9991),c:Clostridia(0.9522),o:Clostridiales(0.8897),f:Ruminococcaceae(0.7302),g:Ruminococcus(0.1150);	-4.17338	1.34E-08	2.63E-08
d:Bacteria(1.0000),p:Firmicutes(0.9998),c:Clostridia(0.9814),o:Clostridiales(0.9715),f:Lachnospiraceae(0.9026),g:Clostridium_XlVa(0.8158);	-3.68563	1.49E-08	2.93E-08
d:Bacteria(1.0000),p:Firmicutes(0.6840),c:Clostridia(0.1500),o:Clostridiales(0.0673),f:Lachnospiraceae(0.0061),g:Lachnospira(0.0043);	3.565788	1.61E-08	3.15E-08
d:Bacteria(1.0000),p:Proteobacteria(0.4731),c:Deltaproteobacteria(0.2113),o:Bdellovibrionales(0.0945),f:Bdellovibrionaceae(0.0296),g:Vampirovibrio(0.0094);	-4.59989	1.69E-08	3.28E-08
d:Bacteria(1.0000),p:Bacteroidetes(1.0000),c:Bacteroidia(1.0000),o:Bacteroidales(1.0000),f:Rikenellaceae(1.0000),g:Alistipes(1.0000);	-8.09694	1.77E-08	3.42E-08
d:Bacteria(1.0000),p:Firmicutes(0.9946),c:Clostridia(0.9207),o:Clostridiales(0.8197),f:Ruminococcaceae(0.4677),g:Sporobacter(0.0637);	-3.68525	1.96E-08	3.78E-08
d:Bacteria(1.0000),p:Bacteroidetes(0.8620),c:Bacteroidia(0.3967),o:Bacteroidales(0.1942),f:Bacteroidaceae(0.0140),g:Bacteroides(0.0060);	-5.91663	2.18E-08	4.18E-08
d:Bacteria(1.0000),p:Bacteroidetes(0.9999),c:Bacteroidia(0.9996),o:Bacteroidales(0.9996),f:Bacteroidaceae(0.9914),g:Bacteroides(0.9914);	-3.79789	2.67E-08	5.09E-08
d:Bacteria(1.0000),p:Bacteroidetes(0.9998),c:Bacteroidia(0.9962),o:Bacteroidales(0.9962),f:Prevotellaceae(0.9898),g:Prevotella(0.9807);	-7.02923	2.70E-08	5.13E-08
d:Bacteria(1.0000),p:Bacteroidetes(0.9946),c:Bacteroidia(0.4593),o:Bacteroidales(0.2305),f:Bacteroidaceae(0.0296),g:Bacteroides(0.0094);	-9.64158	3.12E-08	5.90E-08
d:Bacteria(1.0000),p:Firmicutes(0.9984),c:Negativicutes(0.9935),o:Selenomonadales(0.9904),f:Veillonellaceae(0.9867),g:Dialister(0.9701);	-6.46119	5.12E-08	9.64E-08
d:Bacteria(1.0000),p:Firmicutes(0.8857),c:Clostridia(0.5845),o:Clostridiales(0.3031),f:Clostridiaceae_2(0.0883),g:Alkaliphilus(0.0167);	-3.33652	5.25E-08	9.85E-08
d:Bacteria(1.0000),p:Firmicutes(0.9978),c:Clostridia(0.9299),o:Clostridiales(0.8337),f:Ruminococcaceae(0.5430),g:Sporobacter(0.0865);	-2.73101	5.64E-08	1.05E-07
d:Bacteria(1.0000),p:Bacteroidetes(1.0000),c:Bacteroidia(1.0000),o:Bacteroidales(1.0000),f:Bacteroidaceae(0.9871),g:Bacteroides(0.9871);	-7.78206	5.80E-08	1.08E-07
d:Bacteria(1.0000),p:Bacteroidetes(0.9988),c:Bacteroidia(0.9886),o:Bacteroidales(0.9757),f:Prevotellaceae(0.4275),g:Prevotella(0.0865);	-4.0728	7.35E-08	1.36E-07

d:Bacteria(1.0000),p:Firmicutes(0.7674),c:Clostridia(0.3967),o:Clostridiales(0.1218),f:Ruminococcaceae(0.0454),g:Intestinimonas(0.0077);	-4.61928	8.89E-08	1.64E-07
d:Bacteria(1.0000),p:Firmicutes(0.9938),c:Clostridia(0.8746),o:Clostridiales(0.6422),f:Ruminococcaceae(0.4275),g:Sporobacter(0.0523);	2.463288	1.14E-07	2.09E-07
d:Bacteria(1.0000),p:Firmicutes(0.9802),c:Clostridia(0.8470),o:Clostridiales(0.5099),f:Ruminococcaceae(0.3068),g:Ethanoligenens(0.0215);	2.922194	1.49E-07	2.74E-07
d:Bacteria(1.0000),p:Firmicutes(1.0000),c:Clostridia(0.9968),o:Clostridiales(0.9968),f:Clostridiaceae_1(0.9857),g:Sarcina(0.9722);	-4.33718	1.59E-07	2.90E-07
d:Bacteria(1.0000),p:Firmicutes(0.7674),c:Clostridia(0.3967),o:Clostridiales(0.1942),f:Clostridiaceae_3(0.0668),g:Brassicibacter(0.0143);	-3.61925	1.62E-07	2.95E-07
d:Bacteria(1.0000),p:Firmicutes(0.9987),c:Clostridia(0.9924),o:Clostridiales(0.9799),f:Ruminococcaceae(0.6553),g:Butyricicoccus(0.4804);	2.389421	1.73E-07	3.13E-07
d:Bacteria(1.0000),p:Firmicutes(0.9985),c:Clostridia(0.9958),o:Clostridiales(0.9958),f:Ruminococcaceae(0.9898),g:Faecalibacterium(0.9533);	-3.26028	2.05E-07	3.69E-07
d:Bacteria(1.0000),p:Firmicutes(0.9988),c:Clostridia(0.9559),o:Clostridiales(0.9037),f:Lachnospiraceae(0.7302),g:Clostridium_XlVa(0.4804);	-4.00776	2.43E-07	4.37E-07
d:Bacteria(1.0000),p:Firmicutes(0.9329),c:Clostridia(0.8348),o:Clostridiales(0.4658),f:Ruminococcaceae(0.1861),g:Clostridium_IV(0.0167);	-3.46042	2.96E-07	5.30E-07
d:Bacteria(1.0000),p:Firmicutes(0.9914),c:Clostridia(0.8562),o:Clostridiales(0.5540),f:Ruminococcaceae(0.2263),g:Papillibacter(0.0295);	-2.61821	3.14E-07	5.59E-07
d:Bacteria(1.0000),p:Proteobacteria(1.0000),c:Epsilonproteobacteria(1.0000),o:Campylobacterales(1.0000),f:Helicobacteraceae(0.9973),g:Helicobacter(0.9968);	-3.14221	3.56E-07	6.32E-07
d:Bacteria(1.0000),p:Firmicutes(0.9985),c:Clostridia(0.9933),o:Clostridiales(0.9883),f:Eubacteriaceae(0.7302),g:Eubacterium(0.5751);	-2.48848	4.11E-07	7.27E-07
d:Bacteria(1.0000),p:Firmicutes(0.7911),c:Erysipelotrichia(0.4593),o:Erysipelotrichales(0.2305),f:Erysipelotrichaceae(0.1097),g:Erysipelothrix(0.0191);	2.37665	4.61E-07	8.12E-07
d:Bacteria(1.0000),p:Firmicutes(0.9962),c:Clostridia(0.9023),o:Clostridiales(0.7746),f:Eubacteriaceae(0.3068),g:Eubacterium(0.0751);	2.618831	4.65E-07	8.17E-07
d:Bacteria(1.0000),p:Bacteroidetes(0.9991),c:Bacteroidia(0.8931),o:Bacteroidales(0.7305),f:Prevotellaceae(0.2263),g:Prevotella(0.0523);	-4.28431	4.97E-07	8.70E-07
d:Bacteria(1.0000),p:Firmicutes(0.9991),c:Clostridia(0.9704),o:Clostridiales(0.9597),f:Ruminococcaceae(0.8778),g:Oscillibacter(0.5327);	-2.24092	5.85E-07	1.02E-06
d:Bacteria(1.0000),p:Firmicutes(1.0000),c:Bacilli(1.0000),o:Lactobacillales(0.9966),f:Streptococcaceae(0.9957),g:Streptococcus(0.9957);	-2.00511	6.60E-07	1.15E-06
d:Bacteria(1.0000),p:Bacteroidetes(0.9996),c:Bacteroidia(0.9933),o:Bacteroidales(0.9883),f:Rikenellaceae(0.9847),g:Alistipes(0.9829);	-7.71001	7.80E-07	1.35E-06
d:Bacteria(1.0000),p:Firmicutes(0.9989),c:Clostridia(0.9947),o:Clostridiales(0.9947),f:Lachnospiraceae(0.9439),g:Ruminococcus2(0.8158);	-5.88157	1.01E-06	1.75E-06
d:Bacteria(1.0000),p:Firmicutes(0.9938),c:Clostridia(0.8838),o:Clostridiales(0.6864),f:Ruminococcaceae(0.2665),g:Papillibacter(0.0523);	-2.75953	1.02E-06	1.75E-06
d:Bacteria(1.0000),p:Spirochaetes(0.9938),c:Spirochaetia(0.9115),o:Spirochaetales(0.8057),f:Spirochaetaceae(0.6179),g:Treponema(0.0865);	-4.98554	1.02E-06	1.75E-06
d:Bacteria(1.0000),p:Firmicutes(0.9998),c:Clostridia(0.9933),o:Clostridiales(0.9883),f:Lachnospiraceae(0.9109),g:Blautia(0.6438);	-2.30912	1.06E-06	1.81E-06
d:Bacteria(1.0000),p:Firmicutes(0.9990),c:Clostridia(0.9973),o:Clostridiales(0.9973),f:Ruminococcaceae(0.9939),g:Ruminococcus(0.9935);	-2.60323	1.09E-06	1.84E-06
d:Bacteria(1.0000),p:Firmicutes(0.6840),c:Clostridia(0.3342),o:Clostridiales(0.0673),f:Ruminococcaceae(0.0218),g:Clostridium_IV(0.0077);	-5.03539	1.26E-06	2.13E-06
d:Bacteria(1.0000),p:Firmicutes(0.9930),c:Clostridia(0.8931),o:Clostridiales(0.7305),f:Ruminococcaceae(0.4677),g:Clostridium_IV(0.0637);	2.082758	1.31E-06	2.21E-06
d:Bacteria(1.0000),p:Firmicutes(0.9993),c:Clostridia(0.9391),o:Clostridiales(0.8477),f:Ruminococcaceae(0.6179),g:Intestinimonas(0.1150);	-3.07427	1.37E-06	2.30E-06
d:Bacteria(1.0000),p:Bacteroidetes(0.9999),c:Bacteroidia(0.9957),o:Bacteroidales(0.9957),f:Porphyromonadaceae(0.9957),g:Odoribacter(0.9957);	-5.73435	1.54E-06	2.57E-06
d:Bacteria(1.0000),p:Firmicutes(0.9999),c:Clostridia(0.9996),o:Clostridiales(0.9996),f:Lachnospiraceae(0.9837),g:Blautia(0.7470);	-2.99578	1.63E-06	2.72E-06
d:Bacteria(1.0000),p:Actinobacteria(0.2622),c:Actinobacteria(0.0888),o:Actinomycetales(0.0400),f:Nocardiaceae(0.0140),g:Rhodococcus(0.0060);	-3.65645	1.80E-06	2.98E-06
d:Bacteria(1.0000),p:Firmicutes(0.9985),c:Clostridia(0.9922),o:Clostridiales(0.9778),f:Ruminococcaceae(0.9701),g:Clostridium_IV(0.9701);	-3.30922	2.10E-06	3.47E-06
d:Bacteria(1.0000),p:Firmicutes(0.4731),c:Clostridia(0.2113),o:Clostridiales(0.0945),f:Lachnospiraceae(0.0218),g:Clostridium_XlVa(0.0077);	3.659565	2.47E-06	4.07E-06
d:Bacteria(1.0000),p:Firmicutes(0.9907),c:Clostridia(0.8470),o:Clostridiales(0.5099),f:Clostridiaceae_4(0.1311),g:Thermotalea(0.0215);	-3.34246	2.53E-06	4.15E-06
d:Bacteria(1.0000),p:Firmicutes(0.9329),c:Clostridia(0.7097),o:Clostridiales(0.3775),f:Clostridiaceae_2(0.1311),g:Alkaliphilus(0.0215);	-2.24843	2.67E-06	4.37E-06
d:Bacteria(1.0000),p:Firmicutes(0.9938),c:Clostridia(0.9115),o:Clostridiales(0.8057),f:Ruminococcaceae(0.3872),g:Clostridium_IV(0.0409);	2.012693	4.36E-06	7.10E-06
d:Bacteria(1.0000),p:Firmicutes(0.9986),c:Clostridia(0.9947),o:Clostridiales(0.9947),f:Lachnospiraceae(0.9687),g:Pseudobutyrivibrio(0.8158);	2.241803	5.65E-06	9.18E-06
d:Bacteria(1.0000),p:Firmicutes(0.9999),c:Clostridia(0.9947),o:Clostridiales(0.9947),f:Lachnospiraceae(0.9770),g:Roseburia(0.8502);	-1.62552	5.84E-06	9.45E-06
d:Bacteria(1.0000),p:Bacteroidetes(0.9992),c:Bacteroidia(0.9956),o:Bacteroidales(0.9956),f:Porphyromonadaceae(0.9919),g:Parabacteroides(0.9914);	-3.9771	6.88E-06	1.11E-05
d:Bacteria(1.0000),p:Firmicutes(0.9995),c:Clostridia(0.9926),o:Clostridiales(0.9820),f:Clostridiaceae_1(0.7676),g:Clostridium_sensu_stricto(0.6095);	-3.25477	7.60E-06	1.22E-05
d:Bacteria(1.0000),p:Firmicutes(0.6840),c:Clostridia(0.3342),o:Clostridiales(0.1579),f:Ruminococcaceae(0.0218),g:Ruminococcus(0.0077);	1.876447	7.69E-06	1.23E-05
d:Bacteria(1.0000),p:Tenericutes(0.4731),c:Mollicutes(0.2113),o:Mycoplasmatales(0.0673),f:Mycoplasmataceae(0.0218),g:Mycoplasma(0.0077);	3.566048	8.72E-06	1.39E-05
d:Bacteria(1.0000),p:Firmicutes(0.9987),c:Negativicutes(0.9741),o:Selenomonadales(0.9672),f:Acidaminococcaceae(0.8861),g:Phascolarctobacterium(0.3760);	1.885042	9.25E-06	1.47E-05
d:Bacteria(1.0000),p:Firmicutes(0.9329),c:Clostridia(0.7723),o:Clostridiales(0.4216),f:Clostridiaceae_2(0.1311),g:Alkaliphilus(0.0215);	-2.8747	1.37E-05	2.16E-05
d:Bacteria(1.0000),p:Firmicutes(0.9987),c:Clostridia(0.9954),o:Clostridiales(0.9954),f:Clostridiaceae_1(0.8778),g:Clostridium_sensu_stricto(0.7126);	2.262097	1.43E-05	2.25E-05
d:Bacteria(1.0000),p:Firmicutes(0.7674),c:Clostridia(0.3967),o:Clostridiales(0.1942),f:Ruminococcaceae(0.0454),g:Ruminococcus(0.0119);	2.508969	1.47E-05	2.32E-05
d:Bacteria(1.0000),p:Firmicutes(0.9954),c:Clostridia(0.7097),o:Clostridiales(0.3775),f:Lachnospiraceae(0.1861),g:Syntrophococcus(0.0409);	-2.93086	1.64E-05	2.58E-05
d:Bacteria(1.0000),p:Firmicutes(0.9907),c:Clostridia(0.8654),o:Clostridiales(0.5981),f:Ruminococcaceae(0.2665),g:Sporobacter(0.0215);	-3.11115	1.84E-05	2.87E-05
d:Bacteria(1.0000),p:Tenericutes(0.5785),c:Mollicutes(0.2725),o:Acholeplasmatales(0.1218),f:Acholeplasmataceae(0.0454),g:Acholeplasma(0.0119);	2.370274	2.16E-05	3.36E-05
d:Bacteria(1.0000),p:Bacteroidetes(0.9907),c:Sphingobacteriia(0.6471),o:Sphingobacteriales(0.3394),f:Sphingobacteriaceae(0.1861),g:Pedobacter(0.0167);	2.314203	2.41E-05	3.75E-05
d:Bacteria(1.0000),p:Firmicutes(0.7674),c:Clostridia(0.2725),o:Clostridiales(0.1218),f:Clostridiaceae_1(0.0296),g:Clostridium_sensu_stricto(0.0094);	1.925284	2.50E-05	3.87E-05
d:Bacteria(1.0000),p:Firmicutes(0.9978),c:Bacilli(0.8348),o:Lactobacillales(0.4658),f:Lactobacillaceae(0.3068),g:Lactobacillus(0.0751);	-4.52331	2.62E-05	4.05E-05

d:Bacteria(1.0000),p:Bacteroidetes(0.9566),c:Bacteroidia(0.2725),o:Bacteroidales(0.1218),f:Marinilabiliaceae(0.0454),g:Alkalitalea(0.0119);	-3.25535	2.79E-05	4.29E-05
d:Bacteria(1.0000),p:Bacteroidetes(1.0000),c:Bacteroidia(1.0000),o:Bacteroidales(1.0000),f:Bacteroidaceae(0.9871),g:Bacteroides(0.9871);	-3.68215	2.88E-05	4.40E-05
d:Bacteria(1.0000),p:Firmicutes(0.1875),c:Clostridia(0.0345),o:Clostridiales(0.0136),f:Clostridiales_Incertae_Sedis_XI(0.0061),g:Soehngenia(0.0043);	-3.51931	3.95E-05	6.04E-05
d:Bacteria(1.0000),p:Firmicutes(0.9990),c:Clostridia(0.9299),o:Clostridiales(0.8337),f:Lachnospiraceae(0.5430),g:Anaerostipes(0.3238);	-2.57053	4.35E-05	6.62E-05
d:Bacteria(1.0000),p:Firmicutes(0.4731),c:Clostridia(0.2113),o:Clostridiales(0.0945),f:Clostridiaceae_3(0.0140),g:Sporosalibacterium(0.0060);	-2.71901	4.49E-05	6.81E-05
d:Bacteria(1.0000),p:Bacteroidetes(1.0000),c:Bacteroidia(1.0000),o:Bacteroidales(1.0000),f:Bacteroidaceae(0.9967),g:Bacteroides(0.9967);	-2.96211	4.51E-05	6.81E-05
d:Bacteria(1.0000),p:Firmicutes(0.9983),c:Clostridia(0.9886),o:Clostridiales(0.9757),f:Lachnospiraceae(0.9522),g:Coprococcus(0.8158);	-2.17074	4.68E-05	7.06E-05
d:Bacteria(1.0000),p:Proteobacteria(0.3677),c:Deltaproteobacteria(0.1500),o:Bdellovibrionales(0.0673),f:Bdellovibrionaceae(0.0218),g:Vampirovibrio(0.0077);	-2.80029	4.85E-05	7.28E-05
d:Bacteria(1.0000),p:Firmicutes(0.9997),c:Clostridia(0.9449),o:Clostridiales(0.8617),f:Lachnospiraceae(0.7676),g:Clostridium_XlVa(0.6095);	1.63994	4.90E-05	7.34E-05
d:Bacteria(1.0000),p:Firmicutes(0.3677),c:Clostridia(0.1500),o:Clostridiales(0.0673),f:Lachnospiraceae(0.0218),g:Pseudobutyrivibrio(0.0077);	-2.69841	5.42E-05	8.08E-05
d:Bacteria(1.0000),p:Proteobacteria(0.9998),c:Deltaproteobacteria(0.9924),o:Desulfovibrionales(0.9799),f:Desulfovibrionaceae(0.9701),g:Desulfovibrio(0.9701);	-2.51822	5.51E-05	8.19E-05
d:Bacteria(1.0000),p:Verrucomicrobia(0.9093),c:Subdivision5(0.7723),g:Subdivision5_genera_incertae_sedis(0.0637);	2.608317	5.86E-05	8.66E-05
d:Bacteria(1.0000),p:Bacteroidetes(0.8620),c:Bacteroidia(0.4593),o:Bacteroidales(0.2305),f:Bacteroidaceae(0.0140),g:Bacteroides(0.0060);	2.355119	5.86E-05	8.66E-05
d:Bacteria(1.0000),p:Proteobacteria(0.7674),c:Alphaproteobacteria(0.1500),o:Kiloniellales(0.0400),f:Kiloniellaceae(0.0140),g:Kiloniella(0.0060);	-2.92753	7.96E-05	0.000117
d:Bacteria(1.0000),p:Firmicutes(0.8620),c:Clostridia(0.6471),o:Clostridiales(0.2305),f:Ruminococcaceae(0.1097),g:Clostridium_IV(0.0143);	2.161307	8.93E-05	0.000131
d:Bacteria(1.0000),p:Firmicutes(0.9987),c:Clostridia(0.9962),o:Clostridiales(0.9962),f:Ruminococcaceae(0.9929),g:Faecalibacterium(0.9701);	-2.06113	9.15E-05	0.000134
d:Bacteria(1.0000),p:Firmicutes(0.9990),c:Clostridia(0.9559),o:Clostridiales(0.9037),f:Ruminococcaceae(0.8425),g:Clostridium_IV(0.6438);	-2.85053	0.000121	0.000176
d:Bacteria(1.0000),p:Firmicutes(0.9983),c:Clostridia(0.9952),o:Clostridiales(0.9952),f:Ruminococcaceae(0.9888),g:Faecalibacterium(0.8846);	-2.23963	0.000144	0.00021
d:Bacteria(1.0000),p:Firmicutes(0.9907),c:Clostridia(0.8470),o:Clostridiales(0.5099),f:Clostridiaceae_4(0.1311),g:Geosporobacter(0.0191);	-2.04579	0.000162	0.000235
d:Bacteria(1.0000),p:Firmicutes(0.8147),c:Clostridia(0.5219),o:Clostridiales(0.1579),f:Ruminococcaceae(0.0668),g:Intestinimonas(0.0094);	2.303601	0.000174	0.000251
d:Bacteria(1.0000),p:Tenericutes(0.7674),c:Mollicutes(0.3967),o:Entomoplasmatales(0.1942),f:Spiroplasmataceae(0.0883),g:Spiroplasma(0.0167);	-2.47391	0.000186	0.000268
d:Bacteria(1.0000),p:Firmicutes(0.9802),c:Clostridia(0.8470),o:Clostridiales(0.5099),f:Clostridiales_Incertae_Sedis_XIII(0.1861),g:Anaerovorax(0.0409);	-1.9554	0.000221	0.000317
d:Bacteria(1.0000),p:Proteobacteria(0.5785),c:Alphaproteobacteria(0.2725),o:Rhodospirillales(0.0673),f:Rhodospirillaceae(0.0140),g:Azospirillum(0.0043);	-2.52551	0.000247	0.000354
d:Bacteria(1.0000),p:Firmicutes(0.9962),c:Clostridia(0.9299),o:Clostridiales(0.8337),f:Ruminococcaceae(0.5055),g:Clostridium_IV(0.0523);	1.484301	0.000327	0.000467
d:Bacteria(1.0000),p:Firmicutes(0.8620),c:Clostridia(0.4593),o:Clostridiales(0.2305),f:Lachnospiraceae(0.0454),g:Parasporobacterium(0.0119);	2.098137	0.000363	0.000516
d:Bacteria(1.0000),p:Firmicutes(0.9991),c:Clostridia(0.9207),o:Clostridiales(0.8197),f:Lachnospiraceae(0.5430),g:Roseburia(0.2716);	-2.0916	0.000382	0.000542
d:Bacteria(1.0000),p:Actinobacteria(1.0000),c:Actinobacteria(1.0000),o:Bifidobacteriales(1.0000),f:Bifidobacteriaceae(1.0000),g:Bifidobacterium(0.9893);	-1.77884	0.000414	0.000585
d:Bacteria(1.0000),p:Firmicutes(0.9993),c:Clostridia(0.9960),o:Clostridiales(0.9960),f:Lachnospiraceae(0.9857),g:Lachnospiracea_incertae_sedis(0.9743);	-2.77859	0.000436	0.000614
d:Bacteria(1.0000),p:Firmicutes(0.9996),c:Clostridia(0.9741),o:Clostridiales(0.9672),f:Lachnospiraceae(0.8778),g:Clostridium_XlVa(0.7126);	-2.3615	0.000459	0.000645
d:Bacteria(1.0000),p:Firmicutes(0.9914),c:Clostridia(0.8746),o:Clostridiales(0.5540),f:Ruminococcaceae(0.2665),g:Intestinimonas(0.0295);	1.501843	0.000474	0.000663
d:Bacteria(1.0000),p:Firmicutes(0.3677),c:Erysipelotrichia(0.0888),o:Erysipelotrichales(0.0400),f:Erysipelotrichaceae(0.0140),g:Bulleidia(0.0060);	-2.65159	0.000555	0.000775
d:Bacteria(1.0000),p:Firmicutes(0.9962),c:Clostridia(0.9115),o:Clostridiales(0.8057),f:Ruminococcaceae(0.3872),g:Papillibacter(0.0751);	1.469504	0.000596	0.00083
d:Bacteria(1.0000),p:Firmicutes(0.1651),c:Erysipelotrichia(0.0315),o:Erysipelotrichales(0.0123),f:Erysipelotrichaceae(0.0042),g:Erysipelothrix(0.0034);	-2.26156	0.000605	0.00084
d:Bacteria(1.0000),p:Firmicutes(1.0000),c:Negativicutes(0.9970),o:Selenomonadales(0.9970),f:Veillonellaceae(0.9970),g:Veillonella(0.9871);	-1.9539	0.000635	0.000879
d:Bacteria(1.0000),p:Bacteroidetes(0.9962),c:Bacteroidia(0.9115),o:Bacteroidales(0.8057),f:Prevotellaceae(0.3872),g:Prevotella(0.1150);	-3.55438	0.000678	0.000935
d:Bacteria(1.0000),p:Firmicutes(0.9802),c:Clostridia(0.8562),o:Clostridiales(0.4216),f:Ruminococcaceae(0.2665),g:Intestinimonas(0.0295);	1.988466	0.000818	0.001125
d:Bacteria(1.0000),p:Firmicutes(0.5785),c:Clostridia(0.2113),o:Clostridiales(0.0945),f:Ruminococcaceae(0.0218),g:Intestinimonas(0.0060);	-2.30901	0.000836	0.001147
d:Bacteria(1.0000),p:Firmicutes(0.7674),c:Clostridia(0.3967),o:Clostridiales(0.1942),f:Ruminococcaceae(0.0296),g:Clostridium_III(0.0094);	-2.78916	0.00089	0.001217
d:Bacteria(1.0000),p:Firmicutes(0.9984),c:Clostridia(0.9937),o:Clostridiales(0.9883),f:Ruminococcaceae(0.9837),g:Ruminococcus(0.9786);	2.015231	0.00096	0.001309
d:Bacteria(1.0000),p:Firmicutes(0.8384),c:Clostridia(0.5845),o:Clostridiales(0.3031),f:Ruminococcaceae(0.0883),g:Intestinimonas(0.0167);	1.435442	0.001242	0.001688
d:Bacteria(1.0000),p:Firmicutes(0.7674),c:Erysipelotrichia(0.3342),o:Erysipelotrichales(0.1579),f:Erysipelotrichaceae(0.0668),g:Clostridium_XVIII(0.0143);	-2.14371	0.001438	0.001949
d:Bacteria(1.0000),p:Firmicutes(0.3677),c:Clostridia(0.1500),o:Clostridiales(0.0673),f:Clostridiaceae_3(0.0218),g:Brassicibacter(0.0077);	-1.54146	0.001585	0.002141
d:Bacteria(1.0000),p:Firmicutes(0.9946),c:Clostridia(0.8931),o:Clostridiales(0.7305),f:Ruminococcaceae(0.3872),g:Papillibacter(0.0523);	1.416713	0.001863	0.002509
d:Bacteria(1.0000),p:Firmicutes(0.9995),c:Clostridia(0.9522),o:Clostridiales(0.8897),f:Lachnospiraceae(0.8425),g:Clostridium_XlVa(0.5751);	-2.94206	0.001929	0.00259
d:Bacteria(1.0000),p:Firmicutes(0.9093),c:Clostridia(0.4593),o:Clostridiales(0.2305),f:Lachnospiraceae(0.0668),g:Clostridium_XlVa(0.0143);	-1.92795	0.002476	0.003316
d:Bacteria(1.0000),p:Firmicutes(0.8384),c:Clostridia(0.5845),o:Clostridiales(0.1942),f:Ruminococcaceae(0.0668),g:Clostridium_III(0.0119);	-1.87478	0.002524	0.00337
d:Bacteria(1.0000),p:Firmicutes(0.8620),c:Clostridia(0.6471),o:Clostridiales(0.1942),f:Ruminococcaceae(0.0883),g:Intestinimonas(0.0143);	1.629054	0.002882	0.003837
d:Bacteria(1.0000),p:Firmicutes(0.8147),c:Clostridia(0.5219),o:Clostridiales(0.2668),f:Ruminococcaceae(0.0668),g:Ruminococcus(0.0094);	1.634548	0.003241	0.004302
d:Bacteria(1.0000),p:Firmicutes(0.9954),c:Clostridia(0.8746),o:Clostridiales(0.6422),f:Ruminococcaceae(0.2263),g:Sporobacter(0.0523);	-1.48256	0.003373	0.004465
d:Bacteria(1.0000),p:Firmicutes(0.8147),c:Clostridia(0.3967),o:Clostridiales(0.1942),f:Lachnospiraceae(0.0454),g:Parasporobacterium(0.0119);	1.642852	0.003651	0.004819

d:Bacteria(1.0000),p:Firmicutes(0.8384),c:Clostridia(0.3342),o:Clostridiales(0.1579),f:Clostridiaceae_1(0.0296),g:Clostridium_sensu_stricto(0.0094);	1.346912	0.003896	0.005127
d:Bacteria(1.0000),p:Firmicutes(0.7674),c:Negativicutes(0.2725),o:Selenomonadales(0.1218),f:Veillonellaceae(0.0454),g:Selenomonas(0.0119);	1.390203	0.005098	0.00669
d:Bacteria(1.0000),p:Firmicutes(0.9978),c:Clostridia(0.9391),o:Clostridiales(0.8477),f:Ruminococcaceae(0.6179),g:Oscillibacter(0.0751);	-1.18173	0.005641	0.007371
d:Bacteria(1.0000),p:Proteobacteria(0.4731),c:Deltaproteobacteria(0.2113),o:Bdellovibrionales(0.0945),f:Bdellovibrionaceae(0.0296),g:Vampirovibrio(0.0094);	-1.97909	0.005649	0.007371
d:Bacteria(1.0000),p:Proteobacteria(0.1875),c:Deltaproteobacteria(0.0345),o:Bdellovibrionales(0.0136),f:Bdellovibrionaceae(0.0061),g:Vampirovibrio(0.0043);	-1.75516	0.005993	0.007798
d:Bacteria(1.0000),p:Tenericutes(0.5785),c:Mollicutes(0.2725),o:Entomoplasmatales(0.1218),f:Spiroplasmataceae(0.0454),g:Spiroplasma(0.0119);	-1.80945	0.006283	0.008151
d:Bacteria(0.9753),p:Proteobacteria(0.2622),c:Deltaproteobacteria(0.0888),o:Bdellovibrionales(0.0400),f:Bdellovibrionaceae(0.0140),g:Vampirovibrio(0.0060);	-2.30023	0.006758	0.008743
d:Bacteria(1.0000),p:Firmicutes(0.9991),c:Clostridia(0.9595),o:Clostridiales(0.9177),f:Ruminococcaceae(0.8778),g:Oscillibacter(0.4282);	-1.51395	0.007009	0.009043
d:Bacteria(1.0000),p:Bacteroidetes(0.8620),c:Bacteroidia(0.3967),o:Bacteroidales(0.1942),f:Porphyromonadaceae(0.0668),g:Barnesiella(0.0077);	1.714278	0.007316	0.009412
d:Bacteria(1.0000),p:Bacteroidetes(0.9986),c:Bacteroidia(0.9931),o:Bacteroidales(0.9862),f:Prevotellaceae(0.7676),g:Prevotella(0.3238);	1.60808	0.007627	0.009785

Table S7

Putative taxonomy of OTUs found in all chlorocebus samples. Taxonomic assignment probabilities are in parentheses.

d:Bacteria(1.0000),p:Bacteroidetes(0.9907),c:Sphingobacteriia(0.6471),o:Sphingobacteriales(0.3394),f:Sphingobacteriaceae(0.1861),g:Pedobacter(0.0167);
d:Bacteria(1.0000),p:Bacteroidetes(0.9914),c:Bacteroidia(0.5845),o:Bacteroidales(0.3031),f:Porphyromonadaceae(0.1525),g:Paludibacter(0.0191);
d:Bacteria(1.0000),p:Spirochaetes(0.9988),c:Spirochaetia(0.9886),o:Spirochaetales(0.9757),f:Spirochaetaceae(0.9522),g:Treponema(0.8158);
d:Bacteria(1.0000),p:Bacteroidetes(0.9999),c:Bacteroidia(0.9994),o:Bacteroidales(0.9994),f:Prevotellaceae(0.9935),g:Prevotella(0.9935);
d:Bacteria(1.0000),p:Firmicutes(0.9991),c:Clostridia(0.9975),o:Clostridiales(0.9975),f:Ruminococcaceae(0.9973),g:Faecalibacterium(0.9829);
d:Bacteria(1.0000),p:Firmicutes(0.9946),c:Clostridia(0.8931),o:Clostridiales(0.7305),f:Ruminococcaceae(0.3872),g:Papillibacter(0.0523);
d:Bacteria(1.0000),p:Bacteroidetes(0.9999),c:Bacteroidia(0.9996),o:Bacteroidales(0.9996),f:Bacteroidaceae(0.9914),g:Bacteroides(0.9914);
d:Bacteria(1.0000),p:Firmicutes(0.9962),c:Clostridia(0.9299),o:Clostridiales(0.8337),f:Ruminococcaceae(0.5055),g:Clostridium_IV(0.0523);
d:Bacteria(1.0000),p:Firmicutes(0.7674),c:Negativicutes(0.2725),o:Selenomonadales(0.1218),f:Veillonellaceae(0.0454),g:Selenomonas(0.0119);
d:Bacteria(1.0000),p:Firmicutes(0.9907),c:Clostridia(0.8470),o:Clostridiales(0.5099),f:Ruminococcaceae(0.2665),g:Pseudobacteroides(0.0215);
d:Bacteria(1.0000),p:Firmicutes(1.0000),c:Clostridia(0.9968),o:Clostridiales(0.9968),f:Clostridiaceae_1(0.9857),g:Sarcina(0.9722);
d:Bacteria(1.0000),p:Firmicutes(0.9987),c:Negativicutes(0.9741),o:Selenomonadales(0.9672),f:Acidaminococcaceae(0.8861),g:Phascolarctobacterium(0.3760);
d:Bacteria(1.0000),p:Firmicutes(0.9999),c:Clostridia(0.9947),o:Clostridiales(0.9947),f:Lachnospiraceae(0.9770),g:Roseburia(0.8502);
d:Bacteria(1.0000),p:Actinobacteria(1.0000),c:Actinobacteria(1.0000),o:Bifidobacteriales(1.0000),f:Bifidobacteriaceae(1.0000),g:Bifidobacterium(0.9893);
d:Bacteria(1.0000),p:Proteobacteria(1.0000),c:Gammaproteobacteria(0.9952),o:Enterobacteriales(0.9841),f:Enterobacteriaceae(0.9837),g:Escherichia/Shigella(0.8502);
d:Bacteria(1.0000),p:Firmicutes(0.9914),c:Clostridia(0.8562),o:Clostridiales(0.5540),f:Ruminococcaceae(0.3068),g:Pseudobacteroides(0.0295);
d:Bacteria(1.0000),p:Firmicutes(0.8147),c:Clostridia(0.5219),o:Clostridiales(0.2305),f:Ruminococcaceae(0.0454),g:Ruminococcus(0.0094);
d:Bacteria(1.0000),p:Firmicutes(0.9970),c:Clostridia(0.9207),o:Clostridiales(0.8197),f:Eubacteriaceae(0.4275),g:Eubacterium(0.1672);
d:Bacteria(1.0000),p:Firmicutes(0.9978),c:Clostridia(0.9391),o:Clostridiales(0.8477),f:Ruminococcaceae(0.6179),g:Oscillibacter(0.0751);
d:Bacteria(1.0000),p:Firmicutes(0.9962),c:Clostridia(0.9115),o:Clostridiales(0.8057),f:Ruminococcaceae(0.3872),g:Papillibacter(0.0751);
d:Bacteria(1.0000),p:Proteobacteria(0.4731),c:Deltaproteobacteria(0.2113),o:Bdellovibrionales(0.0945),f:Bdellovibrionaceae(0.0296),g:Vampirovibrio(0.0094);
d:Bacteria(1.0000),p:Firmicutes(0.9992),c:Erysipelotrichia(0.9595),o:Erysipelotrichales(0.9177),f:Erysipelotrichaceae(0.8861),g:Clostridium_XVIII(0.3760);
d:Bacteria(1.0000),p:Firmicutes(0.4731),c:Clostridia(0.2113),o:Clostridiales(0.0945),f:Clostridiaceae_3(0.0140),g:Sporosalibacterium(0.0060);
d:Bacteria(1.0000),p:Firmicutes(0.9914),c:Clostridia(0.8746),o:Clostridiales(0.6422),f:Ruminococcaceae(0.3068),g:Papillibacter(0.0295);
d:Bacteria(1.0000),p:Spirochaetes(0.6840),c:Spirochaetia(0.3342),o:Spirochaetales(0.1579),f:Spirochaetaceae(0.0668),g:Spirochaeta(0.0077);
d:Bacteria(1.0000),p:Firmicutes(1.0000),c:Clostridia(0.9970),o:Clostridiales(0.9970),f:Lachnospiraceae(0.9878),g:Blautia(0.9743);
d:Bacteria(1.0000),p:Firmicutes(0.6840),c:Clostridia(0.3342),o:Clostridiales(0.1579),f:Ruminococcaceae(0.0218),g:Ruminococcus(0.0077);
d:Bacteria(1.0000),p:Firmicutes(0.9093),c:Bacilli(0.5219),o:Bacillales(0.2668),f:Paenibacillaceae_1(0.1097),g:Cohnella(0.0191);
d:Bacteria(1.0000),p:Spirochaetes(0.9988),c:Spirochaetia(0.9886),o:Spirochaetales(0.9757),f:Spirochaetaceae(0.9522),g:Treponema(0.7814);
d:Bacteria(1.0000),p:Firmicutes(0.9914),c:Clostridia(0.8562),o:Clostridiales(0.5540),f:Ruminococcaceae(0.2263),g:Papillibacter(0.0295);
d:Bacteria(1.0000),p:Proteobacteria(1.0000),c:Epsilonproteobacteria(1.0000),o:Campylobacterales(1.0000),f:Helicobacteraceae(0.9973),g:Helicobacter(0.9968);
d:Bacteria(1.0000),p:Firmicutes(0.7674),c:Clostridia(0.3967),o:Clostridiales(0.1942),f:Ruminococcaceae(0.0218),g:Intestinimonas(0.0077);
d:Bacteria(1.0000),p:Firmicutes(1.0000),c:Clostridia(0.9960),o:Clostridiales(0.9960),f:Lachnospiraceae(0.9888),g:Roseburia(0.9786);
d:Bacteria(1.0000),p:Firmicutes(0.9978),c:Clostridia(0.9299),o:Clostridiales(0.8337),f:Ruminococcaceae(0.5430),g:Sporobacter(0.0865);
d:Bacteria(1.0000),p:Firmicutes(0.9962),c:Clostridia(0.9207),o:Clostridiales(0.8197),f:Clostridiales_Incertae_Sedis_XIII(0.4275),g:Mogibacterium(0.1672);
d:Bacteria(1.0000),p:Firmicutes(0.6840),c:Clostridia(0.3342),o:Clostridiales(0.1579),f:Lachnospiraceae(0.0296),g:Coprococcus(0.0077);
d:Bacteria(1.0000),p:Firmicutes(0.9996),c:Clostridia(0.9741),o:Clostridiales(0.9672),f:Lachnospiraceae(0.8778),g:Clostridium_XlVa(0.7126);
d:Bacteria(1.0000),p:Firmicutes(0.9994),c:Clostridia(0.9704),o:Clostridiales(0.9597),f:Lachnospiraceae(0.8051),g:Clostridium_XlVa(0.6095);
d:Bacteria(1.0000),p:Bacteroidetes(0.9987),c:Bacteroidia(0.9704),o:Bacteroidales(0.9597),f:Rikenellaceae(0.9109),g:Alistipes(0.7814);
d:Bacteria(1.0000),p:Firmicutes(0.9999),c:Clostridia(0.9994),o:Clostridiales(0.9994),f:Ruminococcaceae(0.9992),g:Subdoligranulum(0.6438);
d:Bacteria(1.0000),p:Firmicutes(0.9907),c:Clostridia(0.8470),o:Clostridiales(0.5099),f:Clostridiaceae_4(0.1311),g:Geosporobacter(0.0191);
d:Bacteria(1.0000),p:Firmicutes(1.0000),c:Clostridia(0.9952),o:Clostridiales(0.9952),f:Lachnospiraceae(0.9837),g:Dorea(0.9701);
d:Bacteria(1.0000),p:Firmicutes(0.9991),c:Clostridia(0.9207),o:Clostridiales(0.8197),f:Lachnospiraceae(0.5430),g:Roseburia(0.2716);
d:Bacteria(1.0000),p:Firmicutes(0.9991),c:Clostridia(0.9522),o:Clostridiales(0.8897),f:Ruminococcaceae(0.7302),g:Ruminococcus(0.1150);
d:Bacteria(1.0000),p:Tenericutes(0.2622),c:Mollicutes(0.0888),o:Entomoplasmatales(0.0400),f:Spiroplasmataceae(0.0140),g:Spiroplasma(0.0060);
d:Bacteria(1.0000),p:Firmicutes(0.9998),c:Clostridia(0.9922),o:Clostridiales(0.9778),f:Lachnospiraceae(0.8943),g:Clostridium_XlVa(0.6095);
d:Bacteria(1.0000),p:Firmicutes(0.9990),c:Clostridia(0.9937),o:Clostridiales(0.9926),f:Lachnospiraceae(0.9857),g:Coprococcus(0.9829);
d:Bacteria(1.0000),p:Firmicutes(1.0000),c:Clostridia(0.9968),o:Clostridiales(0.9968),f:Lachnospiraceae(0.9770),g:Blautia(0.9743);

d:Bacteria(1.0000),p:Bacteroidetes(0.9988),c:Bacteroidia(0.9922),o:Bacteroidales(0.9778),f:Prevotellaceae(0.4677),g:Prevotella(0.0523);
d:Bacteria(1.0000),p:Firmicutes(0.7911),c:Erysipelotrichia(0.3967),o:Erysipelotrichales(0.1942),f:Erysipelotrichaceae(0.0883),g:Clostridium_XVIII(0.0167);
d:Bacteria(1.0000),p:Firmicutes(0.8384),c:Clostridia(0.5845),o:Clostridiales(0.3031),f:Ruminococcaceae(0.1311),g:Ruminococcus(0.0191);
d:Bacteria(1.0000),p:Firmicutes(0.9946),c:Clostridia(0.9023),o:Clostridiales(0.7746),f:Ruminococcaceae(0.3470),g:Ruminococcus(0.0523);
d:Bacteria(1.0000),p:Firmicutes(0.9987),c:Clostridia(0.9962),o:Clostridiales(0.9962),f:Ruminococcaceae(0.9929),g:Faecalibacterium(0.9701);
d:Bacteria(1.0000),p:Firmicutes(0.9998),c:Clostridia(0.9933),o:Clostridiales(0.9883),f:Lachnospiraceae(0.9109),g:Blautia(0.6438);
d:Bacteria(1.0000),p:Firmicutes(0.9984),c:Clostridia(0.9814),o:Clostridiales(0.9715),f:Lachnospiraceae(0.9357),g:Lachnospiracea_incertae_sedis(0.9189);
d:Bacteria(1.0000),p:Firmicutes(0.9991),c:Clostridia(0.9559),o:Clostridiales(0.9037),f:Lachnospiraceae(0.6928),g:Clostridium_XlVa(0.3760);
d:Bacteria(1.0000),p:Firmicutes(0.9946),c:Clostridia(0.5845),o:Clostridiales(0.3031),f:Clostridiaceae_1(0.1525),g:Anaerosporobacter(0.0295);
d:Bacteria(1.0000),p:Firmicutes(0.9992),c:Clostridia(0.9522),o:Clostridiales(0.8897),f:Ruminococcaceae(0.6553),g:Sporobacter(0.2194);
d:Bacteria(1.0000),p:Firmicutes(0.9329),c:Clostridia(0.8348),o:Clostridiales(0.3031),f:Ruminococcaceae(0.1525),g:Intestinimonas(0.0191);
d:Bacteria(1.0000),p:Firmicutes(0.9993),c:Clostridia(0.9299),o:Clostridiales(0.8337),f:Lachnospiraceae(0.5055),g:Clostridium_XlVa(0.0865);
d:Bacteria(1.0000),p:Firmicutes(0.9998),c:Clostridia(0.9559),o:Clostridiales(0.9037),f:Lachnospiraceae(0.8778),g:Lachnospiracea_incertae_sedis(0.7126);
d:Bacteria(1.0000),p:Bacteroidetes(0.9990),c:Bacteroidia(0.9973),o:Bacteroidales(0.9973),f:Prevotellaceae(0.9191),g:Prevotella(0.8158);
d:Bacteria(1.0000),p:Firmicutes(0.9991),c:Clostridia(0.9704),o:Clostridiales(0.9597),f:Ruminococcaceae(0.8778),g:Oscillibacter(0.5327);
d:Bacteria(1.0000),p:Firmicutes(0.9986),c:Clostridia(0.9115),o:Clostridiales(0.8057),f:Lachnospiraceae(0.5055),g:Clostridium_XlVa(0.2716);
d:Bacteria(1.0000),p:Bacteroidetes(0.9899),c:Sphingobacteriia(0.3342),o:Sphingobacteriales(0.1579),f:Sphingobacteriaceae(0.0668),g:Mucilaginibacter(0.0143);
d:Bacteria(1.0000),p:Firmicutes(0.9999),c:Clostridia(0.9952),o:Clostridiales(0.9952),f:Lachnospiraceae(0.9357),g:Dorea(0.8846);
d:Bacteria(1.0000),p:Firmicutes(0.9995),c:Clostridia(0.9926),o:Clostridiales(0.9820),f:Lachnospiraceae(0.8943),g:Blautia(0.6095);
d:Bacteria(1.0000),p:Firmicutes(0.9990),c:Clostridia(0.9559),o:Clostridiales(0.9037),f:Lachnospiraceae(0.5804),g:Roseburia(0.2716);
d:Bacteria(1.0000),p:Firmicutes(0.8384),c:Clostridia(0.3967),o:Clostridiales(0.1942),f:Ruminococcaceae(0.0454),g:Clostridium_III(0.0119);
d:Bacteria(1.0000),p:Bacteroidetes(0.9922),c:Bacteroidia(0.8348),o:Bacteroidales(0.4658),f:Prevotellaceae(0.1525),g:Prevotella(0.0167);
d:Bacteria(1.0000),p:Firmicutes(0.9992),c:Clostridia(0.9299),o:Clostridiales(0.8337),f:Ruminococcaceae(0.6928),g:Clostridium_IV(0.4282);
d:Bacteria(1.0000),p:Firmicutes(0.9996),c:Clostridia(0.9974),o:Clostridiales(0.9862),f:Ruminococcaceae(0.9604),g:Clostridium_IV(0.4804);
d:Bacteria(1.0000),p:Firmicutes(0.9988),c:Clostridia(0.9886),o:Clostridiales(0.9757),f:Eubacteriaceae(0.8778),g:Eubacterium(0.7126);
d:Bacteria(1.0000),p:Firmicutes(0.9989),c:Clostridia(0.9391),o:Clostridiales(0.8477),f:Lachnospiraceae(0.5804),g:Blautia(0.3238);
d:Bacteria(1.0000),p:Firmicutes(0.9986),c:Clostridia(0.9937),o:Clostridiales(0.9926),f:Lachnospiraceae(0.9687),g:Roseburia(0.6095);
d:Bacteria(1.0000),p:Bacteroidetes(0.9907),c:Bacteroidia(0.5219),o:Bacteroidales(0.2668),f:Porphyromonadaceae(0.1311),g:Paludibacter(0.0215);
d:Bacteria(1.0000),p:Firmicutes(0.9985),c:Clostridia(0.9958),o:Clostridiales(0.9958),f:Ruminococcaceae(0.9898),g:Faecalibacterium(0.9533);
d:Bacteria(1.0000),p:Firmicutes(0.9994),c:Clostridia(0.9983),o:Clostridiales(0.9983),f:Ruminococcaceae(0.9847),g:Faecalibacterium(0.6782);
d:Bacteria(1.0000),p:Firmicutes(0.9996),c:Clostridia(0.9937),o:Clostridiales(0.9926),f:Ruminococcaceae(0.9878),g:Faecalibacterium(0.7126);
d:Bacteria(1.0000),p:Bacteroidetes(0.9994),c:Bacteroidia(0.9937),o:Bacteroidales(0.9926),f:Prevotellaceae(0.9533),g:Prevotella(0.9533);
d:Bacteria(1.0000),p:Firmicutes(0.9990),c:Negativicutes(0.9668),o:Selenomonadales(0.9457),f:Acidaminococcaceae(0.8778),g:Phascolarctobacterium(0.2194);
d:Bacteria(1.0000),p:Firmicutes(0.9938),c:Clostridia(0.8838),o:Clostridiales(0.6864),f:Ruminococcaceae(0.2665),g:Papillibacter(0.0523);
d:Bacteria(1.0000),p:Firmicutes(0.9954),c:Clostridia(0.8746),o:Clostridiales(0.6422),f:Ruminococcaceae(0.2263),g:Sporobacter(0.0523);
d:Bacteria(1.0000),p:Firmicutes(0.9984),c:Clostridia(0.9935),o:Clostridiales(0.9904),f:Ruminococcaceae(0.9837),g:Faecalibacterium(0.8158);
d:Bacteria(1.0000),p:Bacteroidetes(0.9802),c:Bacteroidia(0.5219),o:Bacteroidales(0.2668),f:Porphyromonadaceae(0.1311),g:Paludibacter(0.0191);
d:Bacteria(1.0000),p:Firmicutes(0.9983),c:Clostridia(0.9952),o:Clostridiales(0.9952),f:Ruminococcaceae(0.9888),g:Faecalibacterium(0.8846);

Table S8

Putative taxonomy of OTUs shared between geladas and sheep (assignment probabilities in parentheses)

	% abundance in gelada	% abundance in sheep
d:Bacteria(1.0000),p:Firmicutes(0.5785),c:Erysipelotrichia(0.2725),o:Erysipelotrichales(0.1218),f:Erysipelotrichaceae(0.0454),g:Clostridium_XVIII(0.0119);	1.501	0.094
d:Bacteria(1.0000),p:Bacteroidetes(0.9999),c:Bacteroidia(0.9994),o:Bacteroidales(0.9994),f:Prevotellaceae(0.9935),g:Prevotella(0.9935);	1.413	0.239
d:Bacteria(1.0000),p:Firmicutes(0.9991),c:Clostridia(0.9975),o:Clostridiales(0.9975),f:Ruminococcaceae(0.9973),g:Faecalibacterium(0.9829);	0.255	0.038
d:Archaea(1.0000),p:Euryarchaeota(0.9938),c:Thermoplasmata(0.8654),o:Methanomassiliicoccales(0.4658),f:Methanomassiliicoccaceae(0.3068),g:Methanomassiliicoccus(0.0751);	0.163	0.221
d:Bacteria(1.0000),p:Firmicutes(0.9962),c:Clostridia(0.9299),o:Clostridiales(0.8337),f:Ruminococcaceae(0.5055),g:Clostridium_IV(0.0523);	0.330	0.231
d:Bacteria(1.0000),p:Firmicutes(0.9962),c:Clostridia(0.9023),o:Clostridiales(0.7746),f:Eubacteriaceae(0.3068),g:Eubacterium(0.0751);	0.282	0.056
d:Bacteria(1.0000),p:Firmicutes(0.9999),c:Clostridia(0.9947),o:Clostridiales(0.9947),f:Lachnospiraceae(0.9770),g:Roseburia(0.8502);	0.262	0.024
d:Bacteria(1.0000),p:Tenericutes(0.1875),c:Mollicutes(0.0345),o:Anaeroplasmatales(0.0136),f:Anaeroplasmataceae(0.0061),g:Asteroleplasma(0.0043);	0.171	0.018
d:Bacteria(1.0000),p:Firmicutes(0.7911),c:Erysipelotrichia(0.4593),o:Erysipelotrichales(0.2305),f:Erysipelotrichaceae(0.1097),g:Erysipelothrix(0.0191);	0.094	0.031
d:Bacteria(1.0000),p:Firmicutes(0.9093),c:Bacilli(0.5219),o:Bacillales(0.2668),f:Paenibacillaceae_1(0.1097),g:Cohnella(0.0191);	0.057	0.060
d:Bacteria(1.0000),p:Firmicutes(0.9986),c:Clostridia(0.9947),o:Clostridiales(0.9947),f:Lachnospiraceae(0.9687),g:Pseudobutyrivibrio(0.8158);	0.140	0.118
d:Bacteria(1.0000),p:Firmicutes(0.9329),c:Clostridia(0.5219),o:Clostridiales(0.2668),f:Gracilibacteraceae(0.1311),g:Gracilibacter(0.0215);	0.032	1.669
d:Bacteria(1.0000),p:Firmicutes(0.9978),c:Clostridia(0.9299),o:Clostridiales(0.8337),f:Ruminococcaceae(0.5430),g:Sporobacter(0.0865);	0.031	0.019
d:Bacteria(1.0000),p:Firmicutes(0.9962),c:Clostridia(0.9207),o:Clostridiales(0.8197),f:Clostridiales_Incertae_Sedis_XIII(0.4275),g:Mogibacterium(0.1672);	0.058	0.075
d:Bacteria(1.0000),p:Firmicutes(0.9329),c:Clostridia(0.7723),o:Clostridiales(0.4216),f:Ruminococcaceae(0.1525),g:Clostridium_IV(0.0167);	0.061	0.067
d:Bacteria(1.0000),p:Firmicutes(0.9907),c:Clostridia(0.8470),o:Clostridiales(0.5099),f:Clostridiaceae_4(0.1311),g:Geosporobacter(0.0191);	0.028	0.022
d:Bacteria(1.0000),p:Firmicutes(1.0000),c:Clostridia(0.9968),o:Clostridiales(0.9968),f:Lachnospiraceae(0.9770),g:Blautia(0.9743);	0.018	0.005

Table S9

Putative taxonomy of OTUs found in at least 50% of geladas and 50% of sheep (assignment probabilities in parentheses)

	% abundance in gelada	% abundance in sheep
d:Bacteria(1.0000),p:Firmicutes(0.4731),c:Erysipelotrichia(0.2113),o:Erysipelotrichales(0.0945),f:Erysipelotrichaceae(0.0296),g:Clostridium_XVIII(0.0094);	1.101	0.270
d:Bacteria(1.0000),p:Firmicutes(0.5785),c:Erysipelotrichia(0.2725),o:Erysipelotrichales(0.1218),f:Erysipelotrichaceae(0.0454),g:Clostridium_XVIII(0.0119);	1.501	0.094
d:Bacteria(1.0000),p:Bacteroidetes(0.9907),c:Sphingobacteriia(0.6471),o:Sphingobacteriales(0.3394),f:Sphingobacteriaceae(0.1861),g:Pedobacter(0.0167);	1.842	0.025
d:Bacteria(1.0000),p:Firmicutes(0.8857),c:Clostridia(0.7097),o:Clostridiales(0.3031),f:Ruminococcaceae(0.1097),g:Intestinimonas(0.0119);	1.434	0.008
d:Bacteria(1.0000),p:Bacteroidetes(0.9999),c:Bacteroidia(0.9994),o:Bacteroidales(0.9994),f:Prevotellaceae(0.9935),g:Prevotella(0.9935);	1.413	0.239
d:Bacteria(1.0000),p:Firmicutes(0.9991),c:Clostridia(0.9975),o:Clostridiales(0.9975),f:Ruminococcaceae(0.9973),g:Faecalibacterium(0.9829);	0.255	0.038
d:Bacteria(1.0000),p:Bacteroidetes(0.9999),c:Bacteroidia(0.9996),o:Bacteroidales(0.9996),f:Bacteroidaceae(0.9914),g:Bacteroides(0.9914);	0.080	0.081
d:Archaea(1.0000),p:Euryarchaeota(0.9938),c:Thermoplasmata(0.8654),o:Methanomassiliicoccales(0.4658),f:Methanomassiliicoccaceae(0.3068),g:Methanomassiliicoccus(0.0751);	0.163	0.221
d:Bacteria(1.0000),p:Spirochaetes(0.9970),c:Spirochaetia(0.9449),o:Spirochaetales(0.8617),f:Spirochaetaceae(0.7676),g:Treponema(0.4282);	0.389	0.014
d:Archaea(1.0000),p:Euryarchaeota(0.9999),c:Methanobacteria(0.9996),o:Methanobacteriales(0.9996),f:Methanobacteriaceae(0.9996),g:Methanobrevibacter(0.9957);	0.129	0.545
d:Bacteria(1.0000),p:Firmicutes(0.9962),c:Clostridia(0.9299),o:Clostridiales(0.8337),f:Ruminococcaceae(0.5055),g:Clostridium_IV(0.0523);	0.330	0.231
d:Bacteria(1.0000),p:Firmicutes(0.9962),c:Clostridia(0.9023),o:Clostridiales(0.7746),f:Eubacteriaceae(0.3068),g:Eubacterium(0.0751);	0.282	0.056
d:Archaea(1.0000),p:Euryarchaeota(0.9930),c:Thermoplasmata(0.8562),o:Methanomassiliicoccales(0.4216),f:Methanomassiliicoccaceae(0.2665),g:Methanomassiliicoccus(0.0637);	0.135	0.241
d:Bacteria(1.0000),p:Firmicutes(0.9987),c:Clostridia(0.9960),o:Clostridiales(0.9960),f:Peptostreptococcaceae(0.9786),g:Clostridium_XI(0.9786);	0.268	0.002
d:Bacteria(1.0000),p:Bacteroidetes(0.9329),c:Sphingobacteriia(0.2725),o:Sphingobacteriales(0.1218),f:Sphingobacteriaceae(0.0454),g:Pedobacter(0.0094);	0.144	0.007
d:Bacteria(1.0000),p:Firmicutes(0.9999),c:Clostridia(0.9947),o:Clostridiales(0.9947),f:Lachnospiraceae(0.9770),g:Roseburia(0.8502);	0.262	0.024
d:Bacteria(1.0000),p:Tenericutes(0.1875),c:Mollicutes(0.0345),o:Anaeroplasmatales(0.0136),f:Anaeroplasmataceae(0.0061),g:Asteroleplasma(0.0043);	0.171	0.018
d:Bacteria(1.0000),p:Actinobacteria(1.0000),c:Actinobacteria(1.0000),o:Bifidobacteriales(1.0000),f:Bifidobacteriaceae(1.0000),g:Bifidobacterium(0.9893);	0.112	0.179
d:Bacteria(1.0000),p:Bacteroidetes(1.0000),c:Bacteroidia(1.0000),o:Bacteroidales(1.0000),f:Bacteroidaceae(0.9871),g:Bacteroides(0.9871);	0.003	0.021
d:Bacteria(1.0000),p:Proteobacteria(1.0000),c:Gammaproteobacteria(0.9952),o:Enterobacteriales(0.9841),f:Enterobacteriaceae(0.9837),g:Escherichia/Shigella(0.8502);	0.026	0.010
d:Bacteria(1.0000),p:Firmicutes(0.9914),c:Clostridia(0.8562),o:Clostridiales(0.5540),f:Ruminococcaceae(0.3068),g:Pseudobacteroides(0.0295);	0.380	0.002
d:Bacteria(1.0000),p:Bacteroidetes(1.0000),c:Bacteroidia(1.0000),o:Bacteroidales(1.0000),f:Bacteroidaceae(0.9967),g:Bacteroides(0.9967);	0.143	0.020
d:Bacteria(1.0000),p:Firmicutes(0.9978),c:Clostridia(0.9391),o:Clostridiales(0.8477),f:Ruminococcaceae(0.6179),g:Oscillibacter(0.0751);	0.183	0.005
d:Bacteria(1.0000),p:Firmicutes(0.9962),c:Clostridia(0.9115),o:Clostridiales(0.8057),f:Ruminococcaceae(0.3872),g:Papillibacter(0.0751);	0.148	0.006
d:Bacteria(1.0000),p:Verrucomicrobia(0.9938),c:Subdivision5(0.9115),g:Subdivision5_genera_incertae_sedis(0.4282);	0.116	0.018
d:Bacteria(1.0000),p:Proteobacteria(0.4731),c:Deltaproteobacteria(0.2113),o:Bdellovibrionales(0.0945),f:Bdellovibrionaceae(0.0296),g:Vampirovibrio(0.0094);	0.203	0.037
d:Bacteria(1.0000),p:Firmicutes(0.9992),c:Erysipelotrichia(0.9595),o:Erysipelotrichales(0.9177),f:Erysipelotrichaceae(0.8861),g:Clostridium_XVIII(0.3760);	0.007	0.005
d:Bacteria(1.0000),p:Firmicutes(0.7674),c:Clostridia(0.3967),o:Clostridiales(0.1942),f:Clostridiaceae_1(0.0668),g:Clostridium_sensu_stricto(0.0143);	0.145	0.024
d:Bacteria(1.0000),p:Firmicutes(0.9802),c:Clostridia(0.8470),o:Clostridiales(0.5099),f:Ruminococcaceae(0.3068),g:Ethanoligenens(0.0215);	0.116	0.003
d:Bacteria(1.0000),p:Firmicutes(0.9914),c:Clostridia(0.8746),o:Clostridiales(0.6422),f:Ruminococcaceae(0.3068),g:Papillibacter(0.0295);	0.129	0.002
d:Bacteria(1.0000),p:Firmicutes(0.9329),c:Clostridia(0.7723),o:Clostridiales(0.3394),f:Ruminococcaceae(0.1311),g:Intestinimonas(0.0167);	0.170	0.003
d:Bacteria(1.0000),p:Firmicutes(0.9938),c:Clostridia(0.9023),o:Clostridiales(0.7305),f:Ruminococcaceae(0.2665),g:Papillibacter(0.0637);	0.124	0.003
d:Bacteria(1.0000),p:Firmicutes(0.9962),c:Clostridia(0.9023),o:Clostridiales(0.7746),f:Ruminococcaceae(0.4275),g:Sporobacter(0.0751);	0.138	0.033
d:Bacteria(1.0000),p:Verrucomicrobia(0.8857),c:Subdivision5(0.7097),g:Subdivision5_genera_incertae_sedis(0.0523);	0.100	0.009
d:Bacteria(1.0000),p:Firmicutes(0.9995),c:Clostridia(0.9926),o:Clostridiales(0.9820),f:Clostridiaceae_1(0.7676),g:Clostridium_sensu_stricto(0.6095);	0.005	0.003
d:Bacteria(1.0000),p:Firmicutes(0.5785),c:Clostridia(0.2725),o:Clostridiales(0.1218),f:Clostridiaceae_1(0.0218),g:Clostridium_sensu_stricto(0.0077);	0.228	0.005
d:Bacteria(1.0000),p:Firmicutes(1.0000),c:Clostridia(0.9970),o:Clostridiales(0.9970),f:Lachnospiraceae(0.9878),g:Blautia(0.9743);	0.011	0.013
d:Bacteria(1.0000),p:Firmicutes(0.6840),c:Clostridia(0.3342),o:Clostridiales(0.1579),f:Peptostreptococcaceae(0.0296),g:Acetoanaerobium(0.0077);	0.088	0.041
d:Bacteria(1.0000),p:Firmicutes(0.7911),c:Erysipelotrichia(0.4593),o:Erysipelotrichales(0.2305),f:Erysipelotrichaceae(0.1097),g:Erysipelothrix(0.0191);	0.094	0.031
d:Bacteria(1.0000),p:Firmicutes(0.6840),c:Clostridia(0.3342),o:Clostridiales(0.1579),f:Ruminococcaceae(0.0218),g:Ruminococcus(0.0077);	0.169	0.047
d:Bacteria(1.0000),p:Firmicutes(0.8384),c:Clostridia(0.4593),o:Clostridiales(0.1942),f:Ruminococcaceae(0.0454),g:Clostridium_III(0.0119);	0.065	0.004
d:Bacteria(1.0000),p:Elusimicrobia(0.9986),c:Elusimicrobia(0.9931),o:Elusimicrobiales(0.9862),f:Elusimicrobiaceae(0.9847),g:Elusimicrobium(0.9829);	0.049	0.182
d:Bacteria(1.0000),p:Proteobacteria(0.7674),c:Deltaproteobacteria(0.3967),o:Syntrophobacterales(0.0673),f:Syntrophaceae(0.0218),g:Desulfomonile(0.0077);	0.197	0.012
d:Bacteria(1.0000),p:Firmicutes(0.9093),c:Bacilli(0.5219),o:Bacillales(0.2668),f:Paenibacillaceae_1(0.1097),g:Cohnella(0.0191);	0.057	0.060
d:Bacteria(1.0000),p:Proteobacteria(0.4731),c:Alphaproteobacteria(0.2113),o:Rhodospirillales(0.0400),f:Rhodospirillaceae(0.0140),g:Insolitispirillum(0.0043);	0.103	0.019
d:Bacteria(1.0000),p:Firmicutes(0.9989),c:Clostridia(0.9968),o:Clostridiales(0.9968),f:Peptostreptococcaceae(0.9743),g:Clostridium_XI(0.9743);	0.106	0.008
d:Bacteria(1.0000),p:Firmicutes(0.9978),c:Clostridia(0.9023),o:Clostridiales(0.7746),f:Lachnospiraceae(0.3872),g:Pseudobutyrivibrio(0.0637);	0.078	0.011
d:Bacteria(1.0000),p:Proteobacteria(0.5785),c:Deltaproteobacteria(0.2725),o:Bdellovibrionales(0.0945),f:Bdellovibrionaceae(0.0296),g:Vampirovibrio(0.0094);	0.049	0.005

d:Bacteria(1.0000),p:Firmicutes(0.9914),c:Clostridia(0.8562),o:Clostridiales(0.5540),f:Ruminococcaceae(0.2263),g:Papillibacter(0.0295);	0.033	0.037
d:Bacteria(1.0000),p:Firmicutes(0.9990),c:Clostridia(0.9973),o:Clostridiales(0.9973),f:Ruminococcaceae(0.9939),g:Ruminococcus(0.9935);	0.011	0.066
d:Bacteria(1.0000),p:Firmicutes(0.9986),c:Clostridia(0.9947),o:Clostridiales(0.9947),f:Lachnospiraceae(0.9687),g:Pseudobutyrivibrio(0.8158);	0.140	0.118
d:Bacteria(1.0000),p:Firmicutes(1.0000),c:Clostridia(0.9960),o:Clostridiales(0.9960),f:Lachnospiraceae(0.9888),g:Roseburia(0.9786);	0.010	0.013
d:Bacteria(1.0000),p:Firmicutes(0.9329),c:Clostridia(0.5219),o:Clostridiales(0.2668),f:Gracilibacteraceae(0.1311),g:Gracilibacter(0.0215);	0.032	1.669
d:Bacteria(1.0000),p:Proteobacteria(0.4731),c:Deltaproteobacteria(0.2113),o:Bdellovibrionales(0.0945),f:Bdellovibrionaceae(0.0296),g:Vampirovibrio(0.0094);	0.020	0.005
d:Bacteria(1.0000),p:Firmicutes(0.9978),c:Clostridia(0.9299),o:Clostridiales(0.8337),f:Ruminococcaceae(0.5430),g:Sporobacter(0.0865);	0.031	0.019
d:Bacteria(1.0000),p:Firmicutes(0.9329),c:Clostridia(0.6471),o:Clostridiales(0.3394),f:Ruminococcaceae(0.0883),g:Clostridium_IV(0.0167);	0.037	0.017
d:Bacteria(1.0000),p:Firmicutes(0.9985),c:Clostridia(0.9922),o:Clostridiales(0.9778),f:Ruminococcaceae(0.9357),g:Ruminococcus(0.7814);	0.132	0.007
d:Bacteria(1.0000),p:Firmicutes(0.9983),c:Clostridia(0.9924),o:Clostridiales(0.9799),f:Lachnospiraceae(0.9191),g:Anaerostipes(0.8846);	0.004	0.008
d:Bacteria(1.0000),p:Firmicutes(0.9962),c:Clostridia(0.9207),o:Clostridiales(0.8197),f:Clostridiales_Incertae_Sedis_XIII(0.4275),g:Mogibacterium(0.1672);	0.058	0.075
d:Bacteria(1.0000),p:Firmicutes(0.9329),c:Clostridia(0.7723),o:Clostridiales(0.4216),f:Ruminococcaceae(0.1525),g:Clostridium_IV(0.0167);	0.061	0.067
d:Bacteria(1.0000),p:Proteobacteria(0.3677),c:Deltaproteobacteria(0.1500),o:Bdellovibrionales(0.0673),f:Bdellovibrionaceae(0.0218),g:Vampirovibrio(0.0077);	0.018	0.027
d:Bacteria(1.0000),p:Firmicutes(0.9987),c:Clostridia(0.9922),o:Clostridiales(0.9778),f:Lachnospiraceae(0.9191),g:Clostridium_XlVb(0.7126);	0.032	0.001
d:Bacteria(1.0000),p:Firmicutes(0.6840),c:Clostridia(0.3342),o:Clostridiales(0.1579),f:Lachnospiraceae(0.0296),g:Coprococcus(0.0077);	0.045	0.012
d:Bacteria(1.0000),p:Firmicutes(0.9996),c:Clostridia(0.9741),o:Clostridiales(0.9672),f:Lachnospiraceae(0.8778),g:Clostridium_XlVa(0.7126);	0.014	0.021
d:Bacteria(1.0000),p:Firmicutes(0.9994),c:Clostridia(0.9704),o:Clostridiales(0.9597),f:Lachnospiraceae(0.8051),g:Clostridium_XlVa(0.6095);	0.011	0.002
d:Bacteria(1.0000),p:Firmicutes(0.8384),c:Clostridia(0.5219),o:Clostridiales(0.2668),f:Ruminococcaceae(0.0296),g:Clostridium_III(0.0094);	0.040	0.004
d:Bacteria(1.0000),p:Firmicutes(0.9990),c:Clostridia(0.9964),o:Clostridiales(0.9964),f:Lachnospiraceae(0.9929),g:Coprococcus(0.9893);	0.033	0.009
d:Bacteria(1.0000),p:Firmicutes(0.9914),c:Clostridia(0.8746),o:Clostridiales(0.6422),f:Ruminococcaceae(0.3068),g:Ethanoligenens(0.0523);	0.023	0.010
d:Bacteria(1.0000),p:Firmicutes(0.8384),c:Clostridia(0.3342),o:Clostridiales(0.1579),f:Clostridiaceae_1(0.0296),g:Clostridium_sensu_stricto(0.0094);	0.059	0.010
d:Bacteria(1.0000),p:Firmicutes(0.9907),c:Clostridia(0.8470),o:Clostridiales(0.5099),f:Clostridiaceae_4(0.1311),g:Geosporobacter(0.0191);	0.028	0.022
d:Bacteria(1.0000),p:Firmicutes(0.9962),c:Clostridia(0.9023),o:Clostridiales(0.7746),f:Ruminococcaceae(0.3470),g:Clostridium_III(0.0751);	0.030	0.105
d:Bacteria(1.0000),p:Firmicutes(0.9938),c:Clostridia(0.8931),o:Clostridiales(0.7305),f:Clostridiales_Incertae_Sedis_XIII(0.2263),g:Anaerovorax(0.0523);	0.038	0.022
d:Bacteria(1.0000),p:Tenericutes(0.4731),c:Mollicutes(0.2113),o:Entomoplasmatales(0.0945),f:Entomoplasmataceae(0.0218),g:Mesoplasma(0.0077);	0.020	0.005
d:Bacteria(1.0000),p:Firmicutes(0.9995),c:Clostridia(0.9929),o:Clostridiales(0.9841),f:Clostridiaceae_1(0.8861),g:Clostridium_sensu_stricto(0.6095);	0.033	0.007
d:Bacteria(1.0000),p:Proteobacteria(1.0000),c:Gammaproteobacteria(0.9962),o:Pasteurellales(0.9962),f:Pasteurellaceae(0.9950),g:Haemophilus(0.8158);	0.010	0.001
d:Bacteria(1.0000),p:Firmicutes(1.0000),c:Bacilli(1.0000),o:Lactobacillales(0.9966),f:Streptococcaceae(0.9957),g:Streptococcus(0.9957);	0.008	0.002
d:Bacteria(1.0000),p:Firmicutes(0.8147),c:Negativicutes(0.3342),o:Selenomonadales(0.1579),f:Veillonellaceae(0.0668),g:Dendrosporobacter(0.0143);	0.035	0.014
d:Bacteria(1.0000),p:Tenericutes(0.2622),c:Mollicutes(0.0888),o:Entomoplasmatales(0.0400),f:Spiroplasmataceae(0.0140),g:Spiroplasma(0.0060);	0.004	0.150
d:Bacteria(1.0000),p:Bacteroidetes(0.9999),c:Bacteroidia(0.9992),o:Bacteroidales(0.9992),f:Prevotellaceae(0.9939),g:Prevotella(0.9871);	0.058	0.029
d:Bacteria(1.0000),p:Proteobacteria(0.7911),c:Alphaproteobacteria(0.3342),o:Kiloniellales(0.0400),f:Kiloniellaceae(0.0140),g:Kiloniella(0.0060);	0.044	0.016
d:Bacteria(1.0000),p:Firmicutes(0.9990),c:Clostridia(0.9937),o:Clostridiales(0.9926),f:Lachnospiraceae(0.9857),g:Coprococcus(0.9829);	0.016	0.003
d:Bacteria(1.0000),p:Firmicutes(1.0000),c:Clostridia(0.9968),o:Clostridiales(0.9968),f:Lachnospiraceae(0.9770),g:Blautia(0.9743);	0.018	0.005
d:Bacteria(1.0000),p:Proteobacteria(0.8384),c:Alphaproteobacteria(0.4593),o:Rhizobiales(0.0673),f:Rhodobiaceae(0.0218),g:Afifella(0.0077);	0.021	0.065
d:Bacteria(1.0000),p:Firmicutes(0.9990),c:Clostridia(0.9741),o:Clostridiales(0.9672),f:Clostridiales_Incertae_Sedis_XIII(0.8425),g:Mogibacterium(0.6782);	0.018	0.119
d:Bacteria(1.0000),p:Firmicutes(0.9993),c:Clostridia(0.9486),o:Clostridiales(0.8757),f:Ruminococcaceae(0.6553),g:Sporobacter(0.2194);	0.018	0.008
d:Bacteria(1.0000),p:Firmicutes(0.9970),c:Clostridia(0.9207),o:Clostridiales(0.8197),f:Ruminococcaceae(0.5055),g:Sporobacter(0.0751);	0.008	0.023
d:Bacteria(1.0000),p:Tenericutes(0.4731),c:Mollicutes(0.2113),o:Entomoplasmatales(0.0945),f:Spiroplasmataceae(0.0296),g:Spiroplasma(0.0094);	0.011	0.022
d:Bacteria(1.0000),p:Firmicutes(0.9802),c:Clostridia(0.8348),o:Clostridiales(0.4658),f:Clostridiaceae_2(0.1525),g:Alkaliphilus(0.0295);	0.031	0.004
d:Bacteria(1.0000),p:Firmicutes(0.9986),c:Clostridia(0.9741),o:Clostridiales(0.9672),f:Lachnospiraceae(0.8051),g:Clostridium_XlVa(0.6438);	0.017	0.003
d:Bacteria(1.0000),p:Firmicutes(0.9986),c:Clostridia(0.9926),o:Clostridiales(0.9778),f:Ruminococcaceae(0.9026),g:Intestinimonas(0.6095);	0.012	0.019
d:Bacteria(1.0000),p:Firmicutes(0.9985),c:Clostridia(0.9933),o:Clostridiales(0.9883),f:Eubacteriaceae(0.7302),g:Eubacterium(0.5751);	0.008	0.001
d:Bacteria(1.0000),p:Firmicutes(0.9993),c:Clostridia(0.9486),o:Clostridiales(0.8757),f:Ruminococcaceae(0.6553),g:Clostridium_IV(0.0865);	0.019	0.140
d:Bacteria(1.0000),p:Firmicutes(1.0000),c:Negativicutes(0.9970),o:Selenomonadales(0.9970),f:Veillonellaceae(0.9970),g:Veillonella(0.9871);	0.026	0.006
d:Bacteria(1.0000),p:Proteobacteria(0.3677),c:Deltaproteobacteria(0.1500),o:Bdellovibrionales(0.0673),f:Bdellovibrionaceae(0.0218),g:Vampirovibrio(0.0077);	0.013	0.032
d:Bacteria(0.9753),p:Bacteroidetes(0.5785),c:Bacteroidia(0.0285),o:Bacteroidales(0.0111),f:Prolixibacteraceae(0.0037),g:Prolixibacter(0.0031);	0.020	0.001
d:Bacteria(1.0000),p:Firmicutes(0.9998),c:Clostridia(0.9933),o:Clostridiales(0.9883),f:Lachnospiraceae(0.9109),g:Blautia(0.6438);	0.014	0.002
d:Bacteria(1.0000),p:Firmicutes(0.7911),c:Clostridia(0.3967),o:Clostridiales(0.1579),f:Clostridiales_Incertae_Sedis_XVIII(0.0668),g:Symbiobacterium(0.0143);	0.015	0.161
d:Bacteria(1.0000),p:Tenericutes(0.3677),c:Mollicutes(0.1500),o:Acholeplasmatales(0.0673),f:Acholeplasmataceae(0.0218),g:Acholeplasma(0.0077);	0.023	0.060

d:Bacteria(1.0000),p:Firmicutes(0.9984),c:Clostridia(0.9814),o:Clostridiales(0.9715),f:Lachnospiraceae(0.9357),g:Lachnospiracea_incertae_sedis(0.9189);	0.005	0.003
d:Bacteria(1.0000),p:Firmicutes(0.9985),c:Clostridia(0.9777),o:Clostridiales(0.9693),f:Lachnospiraceae(0.9274),g:Lachnospiracea_incertae_sedis(0.8158);	0.021	0.006
d:Bacteria(1.0000),p:Firmicutes(0.2622),c:Clostridia(0.0888),o:Clostridiales(0.0400),f:Lachnospiraceae(0.0140),g:Clostridium_XlVa(0.0060);	0.022	0.003
d:Bacteria(1.0000),p:Firmicutes(0.9991),c:Clostridia(0.9559),o:Clostridiales(0.9037),f:Lachnospiraceae(0.6928),g:Clostridium_XlVa(0.3760);	0.004	0.004
d:Bacteria(1.0000),p:Firmicutes(0.2622),c:Clostridia(0.0888),o:Clostridiales(0.0400),g:Natranaerovirga(0.0043);	0.005	0.045
d:Bacteria(1.0000),p:Proteobacteria(0.6840),c:Deltaproteobacteria(0.3342),o:Bdellovibrionales(0.1579),f:Bdellovibrionaceae(0.0668),g:Vampirovibrio(0.0143);	0.011	0.005
d:Bacteria(1.0000),p:Firmicutes(0.9992),c:Clostridia(0.9522),o:Clostridiales(0.8897),f:Ruminococcaceae(0.6553),g:Sporobacter(0.2194);	0.004	0.003
d:Bacteria(1.0000),p:Firmicutes(0.9093),c:Clostridia(0.7723),o:Clostridiales(0.4216),f:Lachnospiraceae(0.1311),g:Lachnospiracea_incertae_sedis(0.0215);	0.029	0.013
d:Bacteria(1.0000),p:Proteobacteria(0.7911),c:Deltaproteobacteria(0.4593),o:Bdellovibrionales(0.2305),f:Bdellovibrionaceae(0.1097),g:Vampirovibrio(0.0191);	0.009	0.007
d:Bacteria(1.0000),p:Firmicutes(0.9986),c:Clostridia(0.9449),o:Clostridiales(0.8617),f:Lachnospiraceae(0.5804),g:Blautia(0.0751);	0.009	0.004
d:Bacteria(1.0000),p:Firmicutes(0.9998),c:Clostridia(0.9559),o:Clostridiales(0.9037),f:Lachnospiraceae(0.8778),g:Lachnospiracea_incertae_sedis(0.7126);	0.008	0.011
d:Bacteria(1.0000),p:Verrucomicrobia(0.9566),c:Subdivision5(0.8470),g:Subdivision5_genera_incertae_sedis(0.0865);	0.010	0.029
d:Bacteria(1.0000),p:Firmicutes(0.9922),c:Clostridia(0.8838),o:Clostridiales(0.6864),f:Ruminococcaceae(0.3470),g:Papillibacter(0.0409);	0.009	0.003
d:Bacteria(1.0000),p:Firmicutes(0.9986),c:Clostridia(0.9777),o:Clostridiales(0.9693),f:Lachnospiraceae(0.8425),g:Lachnospiracea_incertae_sedis(0.5751);	0.006	0.001
d:Bacteria(1.0000),p:Firmicutes(0.9989),c:Clostridia(0.9668),o:Clostridiales(0.9457),f:Ruminococcaceae(0.9026),g:Clostridium_IV(0.7126);	0.013	0.003
d:Bacteria(1.0000),p:Firmicutes(0.9984),c:Clostridia(0.9935),o:Clostridiales(0.9904),f:Eubacteriaceae(0.9743),g:Eubacterium(0.9743);	0.017	0.003
d:Bacteria(1.0000),p:Firmicutes(0.4731),c:Clostridia(0.2113),o:Clostridiales(0.0945),f:Clostridiales_Incertae_Sedis_XII(0.0296),g:Guggenheimella(0.0094);	0.016	0.004
d:Bacteria(1.0000),p:Firmicutes(0.9329),c:Clostridia(0.5845),o:Clostridiales(0.3031),f:Clostridiaceae_2(0.1097),g:Alkaliphilus(0.0191);	0.006	0.031
d:Bacteria(1.0000),p:Firmicutes(0.9566),c:Clostridia(0.7097),o:Clostridiales(0.3775),f:Eubacteriaceae(0.1525),g:Alkalibacter(0.0295);	0.007	0.010
d:Bacteria(1.0000),p:Firmicutes(0.8384),c:Clostridia(0.3967),o:Clostridiales(0.1942),f:Ruminococcaceae(0.0454),g:Clostridium_III(0.0119);	0.015	0.009
d:Bacteria(1.0000),p:Firmicutes(0.9962),c:Clostridia(0.8931),o:Clostridiales(0.7305),f:Eubacteriaceae(0.3470),g:Eubacterium(0.0865);	0.012	0.005
d:Bacteria(1.0000),p:Firmicutes(0.9329),c:Clostridia(0.7097),o:Clostridiales(0.3775),f:Clostridiaceae_2(0.1311),g:Alkaliphilus(0.0215);	0.010	0.025
d:Bacteria(1.0000),p:Firmicutes(0.1651),c:Erysipelotrichia(0.0315),o:Erysipelotrichales(0.0123),f:Erysipelotrichaceae(0.0042),g:Erysipelothrix(0.0034);	0.005	0.011
d:Bacteria(1.0000),p:Firmicutes(0.3677),c:Clostridia(0.1500),o:Clostridiales(0.0673),f:Clostridiaceae_3(0.0218),g:Brassicibacter(0.0077);	0.010	0.009
d:Bacteria(1.0000),p:Firmicutes(0.8384),c:Clostridia(0.5219),o:Clostridiales(0.2668),f:Clostridiaceae_2(0.0668),g:Alkaliphilus(0.0143);	0.010	0.013
d:Bacteria(1.0000),p:Firmicutes(0.8384),c:Clostridia(0.3967),o:Clostridiales(0.1942),f:Clostridiaceae_1(0.0668),g:Clostridium_sensu_stricto(0.0143);	0.011	0.009
d:Bacteria(1.0000),p:Firmicutes(0.9930),c:Clostridia(0.6471),o:Clostridiales(0.3394),f:Lachnospiraceae(0.1525),g:Clostridium_XlVa(0.0215);	0.002	0.006
d:Bacteria(1.0000),p:Firmicutes(0.9990),c:Clostridia(0.9631),o:Clostridiales(0.9317),f:Clostridiales_Incertae_Sedis_XIII(0.6928),g:Mogibacterium(0.5327);	0.005	0.003
d:Bacteria(1.0000),p:Firmicutes(0.9992),c:Clostridia(0.9299),o:Clostridiales(0.8337),f:Ruminococcaceae(0.6928),g:Clostridium_IV(0.4282);	0.006	0.001
d:Bacteria(1.0000),p:Firmicutes(0.9987),c:Clostridia(0.9952),o:Clostridiales(0.9952),f:Clostridiaceae_1(0.9837),g:Clostridium_sensu_stricto(0.9722);	0.023	0.004
d:Bacteria(1.0000),p:Firmicutes(0.9986),c:Clostridia(0.9486),o:Clostridiales(0.8757),f:Ruminococcaceae(0.6179),g:Papillibacter(0.1672);	0.004	0.009
d:Bacteria(1.0000),p:Firmicutes(0.9989),c:Clostridia(0.9741),o:Clostridiales(0.9672),f:Lachnospiraceae(0.6928),g:Clostridium_XlVb(0.5327);	0.002	0.009
d:Bacteria(1.0000),p:Firmicutes(0.9329),c:Clostridia(0.7723),o:Clostridiales(0.4216),f:Clostridiaceae_2(0.1311),g:Alkaliphilus(0.0215);	0.005	0.003
d:Bacteria(1.0000),p:Tenericutes(0.6840),c:Mollicutes(0.3342),o:Acholeplasmatales(0.1579),f:Acholeplasmataceae(0.0668),g:Acholeplasma(0.0143);	0.010	0.115
d:Bacteria(1.0000),p:Firmicutes(0.9996),c:Clostridia(0.9741),o:Clostridiales(0.9672),f:Lachnospiraceae(0.8778),g:Clostridium_XlVa(0.7126);	0.005	0.002
d:Bacteria(1.0000),p:Firmicutes(0.9986),c:Clostridia(0.9937),o:Clostridiales(0.9926),f:Lachnospiraceae(0.9687),g:Roseburia(0.6095);	0.007	0.028
d:Bacteria(1.0000),p:Firmicutes(0.9995),c:Clostridia(0.9777),o:Clostridiales(0.9693),f:Lachnospiraceae(0.8425),g:Clostridium_XlVa(0.6782);	0.033	0.001
d:Bacteria(1.0000),p:Firmicutes(0.7674),c:Erysipelotrichia(0.3967),o:Erysipelotrichales(0.1942),f:Erysipelotrichaceae(0.0883),g:Clostridium_XVIII(0.0167);	0.368	0.029
d:Bacteria(1.0000),p:Firmicutes(1.0000),c:Bacilli(1.0000),o:Lactobacillales(0.9962),f:Streptococcaceae(0.9898),g:Streptococcus(0.9871);	0.010	0.010
d:Bacteria(1.0000),p:Firmicutes(0.2622),c:Erysipelotrichia(0.0345),o:Erysipelotrichales(0.0136),f:Erysipelotrichaceae(0.0061),g:Erysipelothrix(0.0043);	0.004	0.044
d:Bacteria(1.0000),p:Firmicutes(0.9989),c:Clostridia(0.9486),o:Clostridiales(0.8757),f:Lachnospiraceae(0.8051),g:Lachnospiracea_incertae_sedis(0.5751);	0.004	0.004
d:Bacteria(1.0000),p:Firmicutes(0.9991),c:Clostridia(0.9595),o:Clostridiales(0.8897),f:Ruminococcaceae(0.6553),g:Papillibacter(0.1672);	0.019	0.001
d:Bacteria(1.0000),p:Firmicutes(0.9999),c:Clostridia(0.9996),o:Clostridiales(0.9996),f:Clostridiaceae_1(0.9857),g:Clostridium_sensu_stricto(0.9786);	0.030	0.004
d:Bacteria(1.0000),p:Firmicutes(0.9986),c:Clostridia(0.8746),o:Clostridiales(0.6422),f:Lachnospiraceae(0.4677),g:Lachnospiracea_incertae_sedis(0.0865);	0.003	0.004
d:Bacteria(1.0000),p:Firmicutes(0.9998),c:Clostridia(0.9933),o:Clostridiales(0.9883),f:Lachnospiraceae(0.8943),g:Blautia(0.6782);	0.013	0.004
d:Bacteria(1.0000),p:Firmicutes(0.8857),c:Clostridia(0.6471),o:Clostridiales(0.3394),f:Clostridiaceae_2(0.0883),g:Alkaliphilus(0.0167);	0.003	0.050
d:Bacteria(1.0000),p:Bacteroidetes(0.9999),c:Bacteroidia(0.9995),o:Bacteroidales(0.9995),f:Prevotellaceae(0.9967),g:Prevotella(0.9967);	0.016	0.013
d:Bacteria(1.0000),p:Firmicutes(0.9999),c:Clostridia(0.9933),o:Clostridiales(0.9883),f:Lachnospiraceae(0.9191),g:Ruminococcus2(0.7814);	0.005	0.002
d:Bacteria(1.0000),p:Firmicutes(0.8620),c:Bacilli(0.3342),o:Bacillales(0.1579),f:Bacillaceae_1(0.0668),g:Bacillus(0.0143);	0.010	0.003
d:Bacteria(1.0000),p:Firmicutes(0.9978),c:Clostridia(0.9391),o:Clostridiales(0.8477),f:Ruminococcaceae(0.6553),g:Oscillibacter(0.0751);	0.002	0.041

d:Bacteria(1.0000),p:Firmicutes(0.9938),c:Clostridia(0.8931),o:Clostridiales(0.7305),f:Ruminococcaceae(0.3068),g:Sporobacter(0.0523);	0.018	0.002
d:Bacteria(1.0000),p:Firmicutes(0.7911),c:Clostridia(0.3342),o:Clostridiales(0.1579),f:Clostridiaceae_1(0.0454),g:Caloramator(0.0119);	0.003	0.003
d:Bacteria(1.0000),p:Proteobacteria(0.5785),c:Deltaproteobacteria(0.2725),o:Bdellovibrionales(0.1218),f:Bdellovibrionaceae(0.0454),g:Vampirovibrio(0.0119);	0.031	0.006
d:Bacteria(1.0000),p:Firmicutes(0.9930),c:Clostridia(0.8931),o:Clostridiales(0.7305),f:Ruminococcaceae(0.2665),g:Clostridium_III(0.0637);	0.004	0.032
d:Bacteria(1.0000),p:Firmicutes(0.9991),c:Clostridia(0.9391),o:Clostridiales(0.8477),f:Lachnospiraceae(0.4677),g:Clostridium_XlVa(0.1672);	0.010	0.004
d:Bacteria(1.0000),p:Firmicutes(0.9983),c:Clostridia(0.9931),o:Clostridiales(0.9862),f:Lachnospiraceae(0.9357),g:Pseudobutyrivibrio(0.7470);	0.006	0.007
d:Bacteria(1.0000),p:Firmicutes(0.9992),c:Clostridia(0.9486),o:Clostridiales(0.8757),f:Ruminococcaceae(0.6553),g:Clostridium_IV(0.0865);	0.007	0.003
d:Bacteria(1.0000),p:Firmicutes(0.9930),c:Clostridia(0.8470),o:Clostridiales(0.5099),f:Ruminococcaceae(0.1861),g:Sporobacter(0.0295);	0.006	0.004
d:Bacteria(1.0000),p:Firmicutes(0.9986),c:Clostridia(0.9299),o:Clostridiales(0.8337),f:Ruminococcaceae(0.5430),g:Ruminococcus(0.2194);	0.015	0.003
d:Bacteria(1.0000),p:Firmicutes(0.9930),c:Clostridia(0.9023),o:Clostridiales(0.7746),f:Ruminococcaceae(0.3872),g:Clostridium_IV(0.0295);	0.002	0.037
d:Bacteria(1.0000),p:Firmicutes(0.9329),c:Clostridia(0.5845),o:Clostridiales(0.3031),f:Clostridiaceae_1(0.1311),g:Clostridium_sensu_stricto(0.0215);	0.009	0.025
d:Bacteria(1.0000),p:Firmicutes(0.9993),c:Clostridia(0.9449),o:Clostridiales(0.8617),f:Ruminococcaceae(0.6179),g:Ruminococcus(0.4282);	0.008	0.003
d:Bacteria(1.0000),p:Firmicutes(0.7911),c:Erysipelotrichia(0.4593),o:Erysipelotrichales(0.2305),f:Erysipelotrichaceae(0.1097),g:Clostridium_XVIII(0.0191);	0.003	0.253
d:Bacteria(1.0000),p:Firmicutes(0.3677),c:Clostridia(0.1500),o:Clostridiales(0.0673),f:Lachnospiraceae(0.0218),g:Pseudobutyrivibrio(0.0077);	0.004	0.005

Table S10

Family level contributions to main cellulolytic enzymes in geladas and sheep.

Each column indicates the percentage of the indicated enzyme group that could be traced back to the bacterial families indicated on the left.

	β-1,4-endoglucanases		β-1,4-exoglucanases		β-glucosidases	
	Gelada	Sheep	Gelada	Sheep	Gelada	Sheep
Ruminococcaceae	44.70 %	26.60 %	82 %	84.40 %	41.60 %	35.60 %
Clostridiaceae	7.80 %	2.90 %	15.70 %	15.50 %	10.90 %	4.40 %
Lachnospiraceae	9.90 %	13.30 %	2.10 %	0 %	14.10 %	23.70 %
Prevotellaceae	0.30 %	10.30 %	0 %	0 %	0 %	0 %
Bacteroidaceae	1.60 %	2.60 %	0 %	0 %	0 %	0 %
Erysipelotrichaceae	0 %	0 %	0 %	0 %	5 %	8.10 %
